# The *ftsA* gene as a molecular marker for phylogenetic studies in *Bradyrhizobium* and identification of *Bradyrhizobium japonicum*

**DOI:** 10.1007/s13353-018-0479-9

**Published:** 2018-11-11

**Authors:** Michał Kalita, Wanda Małek

**Affiliations:** Department of Genetics and Microbiology, M. Curie-Sklodowska University, Akademicka 19, 20-033 Lublin, Poland

**Keywords:** *Bradyrhizobium*, Phylogenetic analysis, *ftsA*, Single-nucleotide polymorphism

## Abstract

The use of *ftsA* gene sequences for taxonomic studies of the genus *Bradyrhizobium* bacteria was assessed. The *ftsA* gene codes for an actin-like protein involved in prokaryotic cell division. Up to now, this gene has not been used as a phylogenetic marker for analysis of bacteria establishing root nodule symbiosis with Fabaceae plants. In this study, the *ftsA* gene sequences obtained for bradyrhizobia forming N_2_ fixing symbiosis with four Genisteae tribe plants growing in Poland and most of the type strains of the genus *Bradyrhizobium* species were analyzed and evaluated as molecular markers for phylogenetic studies of these bacteria for the first time. The *ftsA* gene sequences of all bradyrhizobial strains with completely or partially sequenced genomes, available in the GenBank database, were also included into the analysis. The phylogeny of the *ftsA* gene was compared to the phylogenies of other chromosomal genes commonly used in the studies of *Bradyrhizobium* bacteria. The results showed that the phylogenies of *ftsA* and the core genes *recA* and *glnII* were congruent, making the *ftsA* gene useful as a phylogenetic marker. Analysis of the *ftsA* gene sequences revealed a single-nucleotide polymorphism unique to *Bradyrhizobium japonicum* strains, and the potential use of this SNP for identification of this species was discussed.

**Electronic supplementary material:**

The online version of this article (10.1007/s13353-018-0479-9) contains supplementary material, which is available to authorized users.

## Introduction

Bacteria belonging to the genus *Bradyrhizobium* and able to fix N_2_ in symbiosis with leguminous plants form a monophyletic group within the α class of Proteobacteria together with oligotrophic soil and aquatic bacteria (Durán et al. 2012). The development and introduction of several molecular techniques to the taxonomic studies of bacteria helped to identify high genetic diversity among *Bradyrhizobium* strains. For the phylogenetic reconstructions of the genus *Bradyrhizobium* bacteria, the *atpD*, *dnaK*, *gyrB*, *glnII*, *recA*, and *rpoB* markers have been commonly used (Delamuta et al. 2012; Kalita and Małek 2017; Menna et al. 2009; Rivas et al. 2009; Stępkowski et al. 2003; Vinuesa et al. 2005). The *ftsA* gene sequences have not been used in the phylogenetic analysis of root nodule bacteria. The FtsA protein functions at the earliest stage of bacterial division, connecting FtsZ, the principal component of the division machinery, to the cell membrane, and forms a structure called the proto-ring at the division site (Fujita et al. 2014). FtsA belongs structurally to the actin/Hsp70/hexokinase superfamily and is widespread in bacteria (Busiek and Margolin 2015). The functional preservation and universal distribution among bacteria make the *ftsA* gene suitable for inferring phylogenetic relationships.

The aim of the present study was to estimate the degree of *ftsA* gene sequence conservation among the genus *Bradyrhizobium* strains and to determine whether the *ftsA* gene could be used as a new marker in the phylogenetic analysis of *Bradyrhizobium* species. The availability of several fully and partially genome-sequenced strains allowed us to address this issue using in silico analysis. The *ftsA* phylogeny of bradyrhizobia was discussed in comparison to the phylogenies of other chromosomal genes *glnII*, and *recA*.

## Materials and methods

### PCR and sequencing

The region of 1140 bp length of *ftsA* gene was amplified using primers ftsAF (5’-ATCGGYTACAGCCAGATCCAGT-3′) and ftsAR (5’-CCTCGCGTAGCCATCGTCCRA-3′). The PCR protocol was as follows: 3 min of initial denaturation carried out at 95 °C followed by 35 cycles of 1 min at 95 °C, 30 s at 58 °C, 1 min at 72 °C with the final elongation step of 7 min at 72 °C. The amplified products were sequenced in both directions with ftsAF/fstAR primers using BigDye Terminator Cycle sequencing kit and the 3500 Genetic Analyzer according to the manufacturer’s procedures (Thermo Fisher Scientific).

### Sequence data analysis

The *ftsA* gene sequences obtained in this study were deposited in the GenBank database under the accession numbers provided in Supplementary Table S1. The *ftsA*, *glnII*, and *recA* gene sequences of completely or partially sequenced genomes of *Bradyrhizobium* strains were obtained from NCBI Genome database. The complete list of strains and their accession numbers is available in Table S1. All the phylogenetic analyses were conducted in MEGA 7 (Kumar et al. 2016). Sequence identity values for single genes were calculated using BioEdit software (Hall 2011). The 2-D matrices generated in BioEdit were edited manually and converted into tables in Excel (Supplementary Tables S2-S5).

## Results and discussion

Phylogenetic analysis was carried out using 733 bp long fragments of the *ftsA* gene of 69 bradyrhizobial strains encompassing sequences of eight strains isolated from root nodules of four Genisteae tribe plants growing in Poland, and 61 sequences of bradyrhizobial strains affiliated to 28 species of the genus *Bradyrhizobium* of which 44 were retrieved from the NCBI genomic database and 17 sequences were generated during this study.

**Fig. 1:**
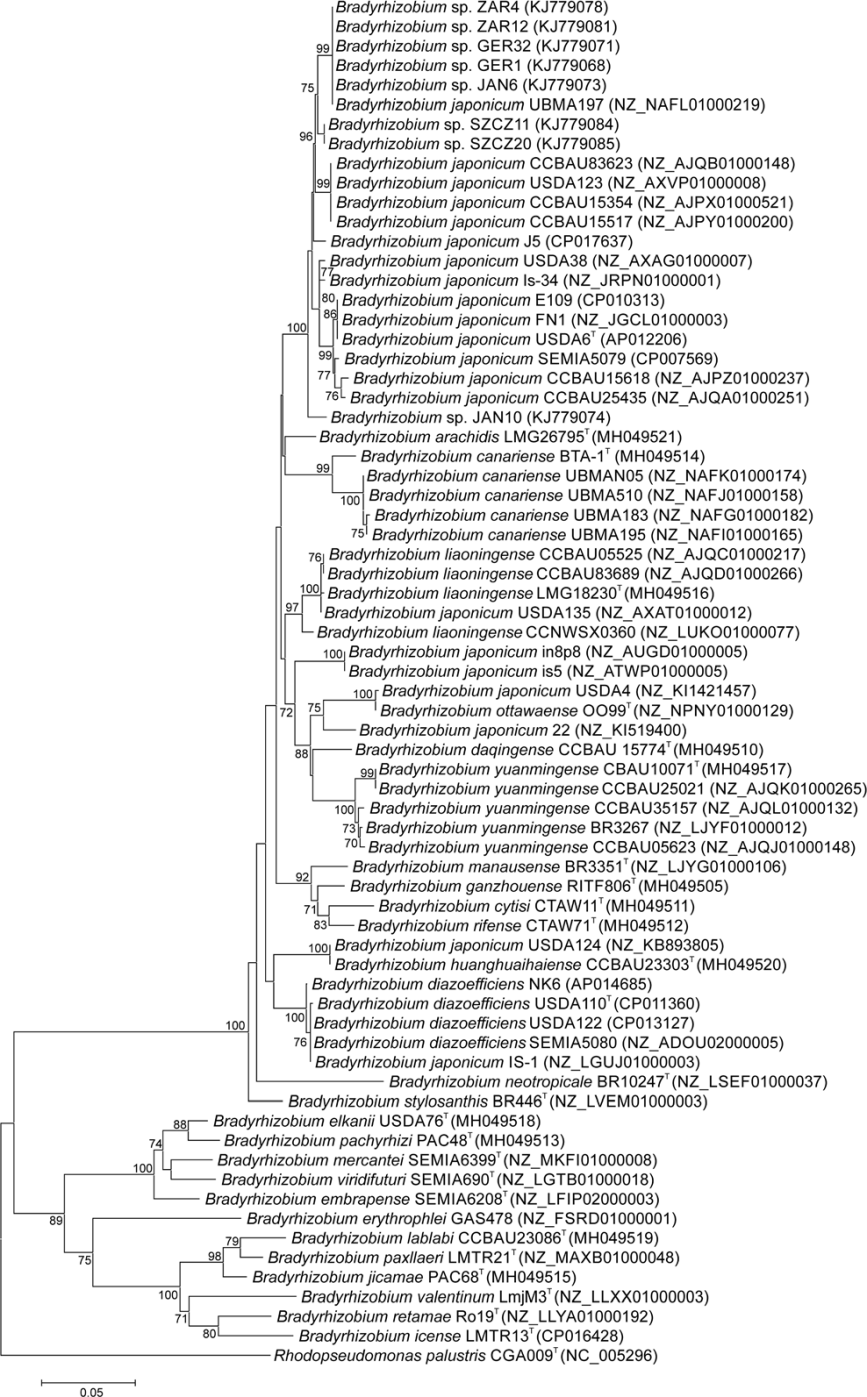
Maximum likelihood phylogenetic tree of *ftsA* gene sequences of *Bradyrhizobium* strains. Bootstrap values ≥ 70% are given at branching points. The scale bar indicates the number of substitution per site

The *ftsA* sequences divided the analyzed strains into two distinct groups, as shown on the phylogenetic tree (Fig. 1). One group consisted of 15 bradyrhizobial species, among others, *B. japonicum*, *B. diazoefficiens*, *B. canariense*, *B. yuanmingense*, and *B. liaoningense*. All root nodule isolates of the Genisteae plant species were positioned with strains representing *B. japonicum*. *B. elkanii*, *B. erythrophlei*, *B. valentinum*, and *B. lablabi* were assigned to the other group. In a similar way, all bradyrhizobia were grouped in the phylograms of the *glnII* and *recA* genes (Supplementary Figs. S1 and S2), which are commonly used as phylogenetic markers in the studies of *Bradyrhizobium* bacteria (Kalita and Małek 2017; Menna et al. 2009; Rivas et al. 2009).

To assess the resolving power of the *ftsA* marker at the species level, we analyzed several sequences retrieved from fully or partially sequenced genomes of strains belonging to different *Bradyrhizobium* species. As can be seen on the *ftsA* phylogenetic tree, strains representing *B. canariense*, *B. diazoefficiens*, *B. liaoningense*, and *B. yuanmingense* form very well-resolved clusters (Fig. 1). This clustering is supported by the *glnII* and *recA* phylogenies (Supplementary Figs. S1 and S2). The phylogenetic analysis of the *ftsA* sequences of 21 strains named *Bradyrhizobium japonicum* demonstrated that 14 of them were placed on the *ftsA* phylogram in a common cluster together with the type strain *B. japonicum* USDA 6^T^ (Fig. 1). The other seven strains were grouped with other species of the genus *Bradyrhizobium* or were placed on separate branches (Fig. 1). This scattered position of the *B. japonicum* strains on the *ftsA* tree is supported by the *glnII* and *recA* phylogenies (Supplementary Figs. S1 and S2). This led us to a conclusion that the nomenclature of the strains currently named *B. japonicum* and found outside the *B. japonicum* species cluster requires revision.

The level of *ftsA* gene sequence diversity was assessed by calculating the number and percentage of variable positions in the alignment. There were 286 variable characters in the *ftsA* alignment, which corresponds to 38.8% of all nucleotide positions included in the analysis. This value was higher than the number of variable positions estimated for the *glnII* (31.2%) and *recA* (31.4%) alignments, indicating that the *ftsA* gene sequence analysis yields more phylogenetic information. The interspecific level of *ftsA* sequence similarity ranged from 80 to 97.4% and was comparable with *glnII* (84.1–98.1%) and *recA* (88.5–97%) genes. The intraspecific *ftsA* sequence similarity ranged from 97.1 to 100%. A similar range of sequence variation at the species level was observed for *glnII* (96.6–100%) and *recA* (96–100%) genes (Supplementary Tables S2-S4). The highest values of interspecific and the lowest value of intraspecific sequence similarity overlap, which means that there are no gaps that allow distinguishing between different species.

Since the phylogenetic analysis of *ftsA* gene sequences has proven to be reliable in discrimination of closely related *Bradyrhizobium* strains, we decided to evaluate the usefulness of this marker in species identification. As the *ftsA* sequences of *B. japonicum* strains were most commonly represented in our analysis, we have carefully checked the *ftsA* sequence alignment to see whether there are any polymorphisms unique to this species. The single-nucleotide polymorphism (SNP) has been used earlier in identification of bacterial species. For example, the SNP analysis of 16S rRNA gene sequences was used for *Bacillus cereus* and *Bacillus anthracis* discrimination (Hakovirta et al. 2016). The SNP analysis of three genes was used for distinguishing closely related species of the genus *Brucella* (Scott et al. 2007). It was also demonstrated that a single-nucleotide polymorphism in the *rpoB* gene allows specific identification of *Salmonella enterica* serotype Typhimurium (Hernandez Guijarro et al. 2012).

**Fig. 2:**
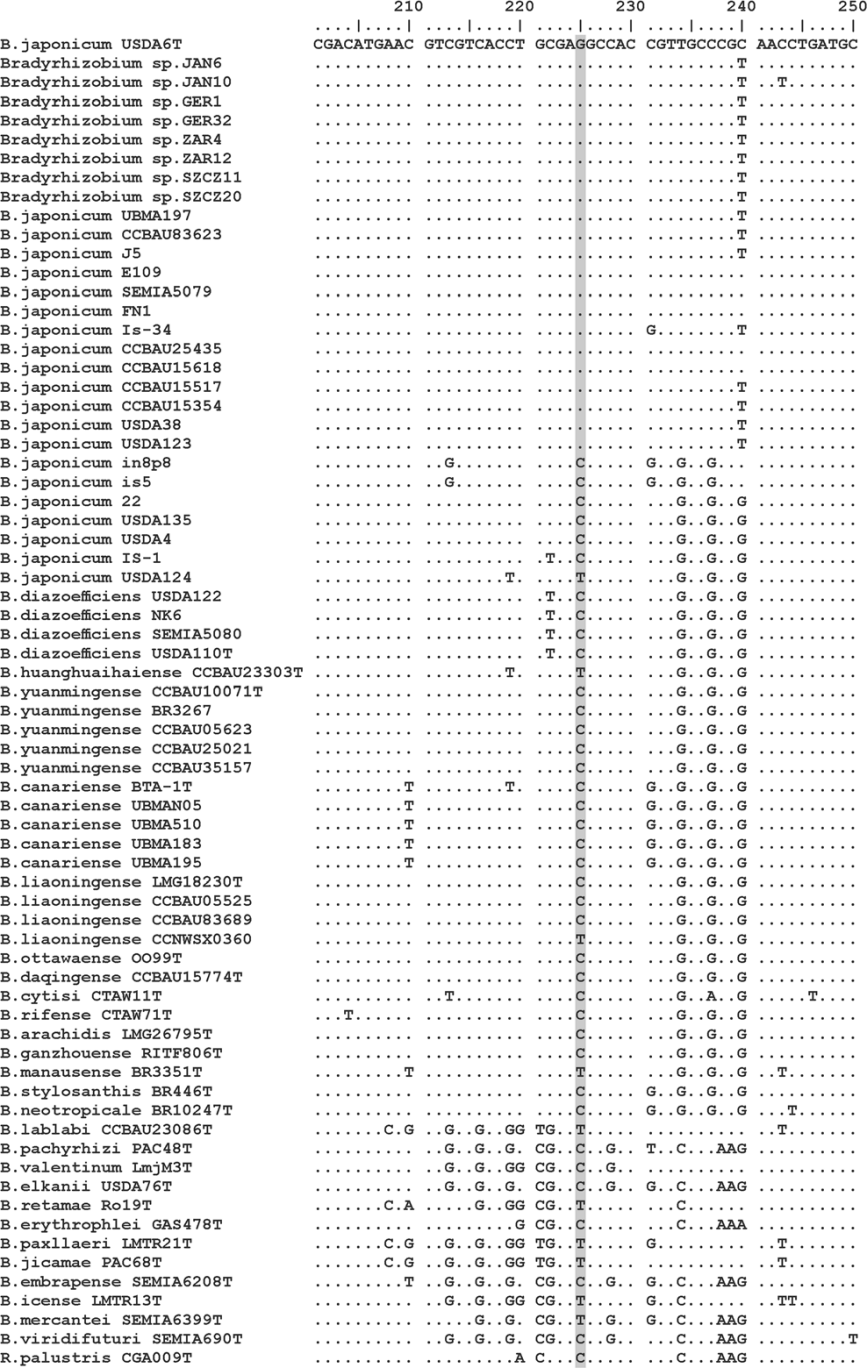
Nucleotide sequence alignment of the *ftsA* gene fragments. Only differences relative to the top sequence (*B. japonicum* USDA 6^T^) are shown. The shaded nucleotide position 225 corresponds to the single-nucleotide polymorphism (guanine) identified in *Bradyrhizobium japonicum* strains

All 22 *B. japonicum* strains including eight isolates from root nodules of the Genisteae plants have guanine at position 225 of the *ftsA* alignment, whereas other bradyrhizobia, including seven misnamed *B. japonicum* strains, have cytosine or thymine (Fig. 2). This single-nucleotide polymorphism corresponding to position 561 in the *ftsA* gene of *Bradyrhizobium japonicum* USDA 6^T^ occurs at the third position of the 187th codon and results in nonsynonymous substitution.

As the observed SNP appeared to be particularly promising for the identification of *B. japonicum*, we decided to broaden the analysis by comparison of the *ftsA* gene sequences retrieved from both complete and partially sequenced bradyrhizobial genomes available in the NCBI Genome database. As a result, 176 *ftsA* gene sequences obtained from 97 strains affiliated to 34 known bradyrhizobial species, 76 strains named *Bradyrhizobium* sp., and three *Bosea* reference sequences were aligned and checked for the SNP at position 225. Guanine at position 225 was observed in the *ftsA* sequence of *Bradyrhizobium* sp. G22 strain in addition to the 22 *B. japonicum* strains mentioned previously (Supplementary Fig. S3). The *ftsA* sequence of *Bradyrhizobium* sp. G22 was most similar to the *ftsA* gene sequences of *B. japonicum* (97–100%) (Supplementary Table S5). Jones et al. (2016) demonstrated that *Bradyrhizobium* sp. G22 is closely related to *B. japonicum* USDA 6^T^ and *B. japonicum* E109. *Bradyrhizobium* sp. G22 is also positioned within the *B. japonicum* cluster on the *ftsA* phylogram reconstructed with 176 sequences (Supplementary Fig. S4).

In conclusion, we have demonstrated that the *ftsA* gene may serve as a useful molecular marker in phylogenetic and taxonomic studies of genus *Bradyrhizobium* bacteria. It holds enough phylogenetic information to distinguish closely related species and, at the same time, it is sufficiently conserved at the intraspecific level allowing correct clustering of strains belonging to a single species. The results of the comparative *ftsA* sequence analysis suggest that the presence of guanine at nucleotide position 561 of the full length gene sequence could be considered as a unique feature of *Bradyrhizobium japonicum* strains. More studies with more bradyrhizobial isolates should provide the evidence whether this SNP could be used as an additional marker for identification of *B. japonicum* bacteria within genus *Bradyrhizobium* populations.

## Electronic supplementary material

ESM 1 (PDF 5017 kb)
