# Supplementary material for: The ftsA gene as a molecular marker for phylogenetic studies in Bradyrhizobium and identification of Bradyrhizobium japonicum
Source: J Appl Genet. 2018 Nov 11;60(1):123–6. doi: 10.1007/s13353-018-0479-9 (PMC6373400; doi:10.1007/s13353-018-0479-9)
Supplement: Supplementary file 1 — (PDF 5017 kb) [file 13353_2018_479_MOESM1_ESM.pdf]

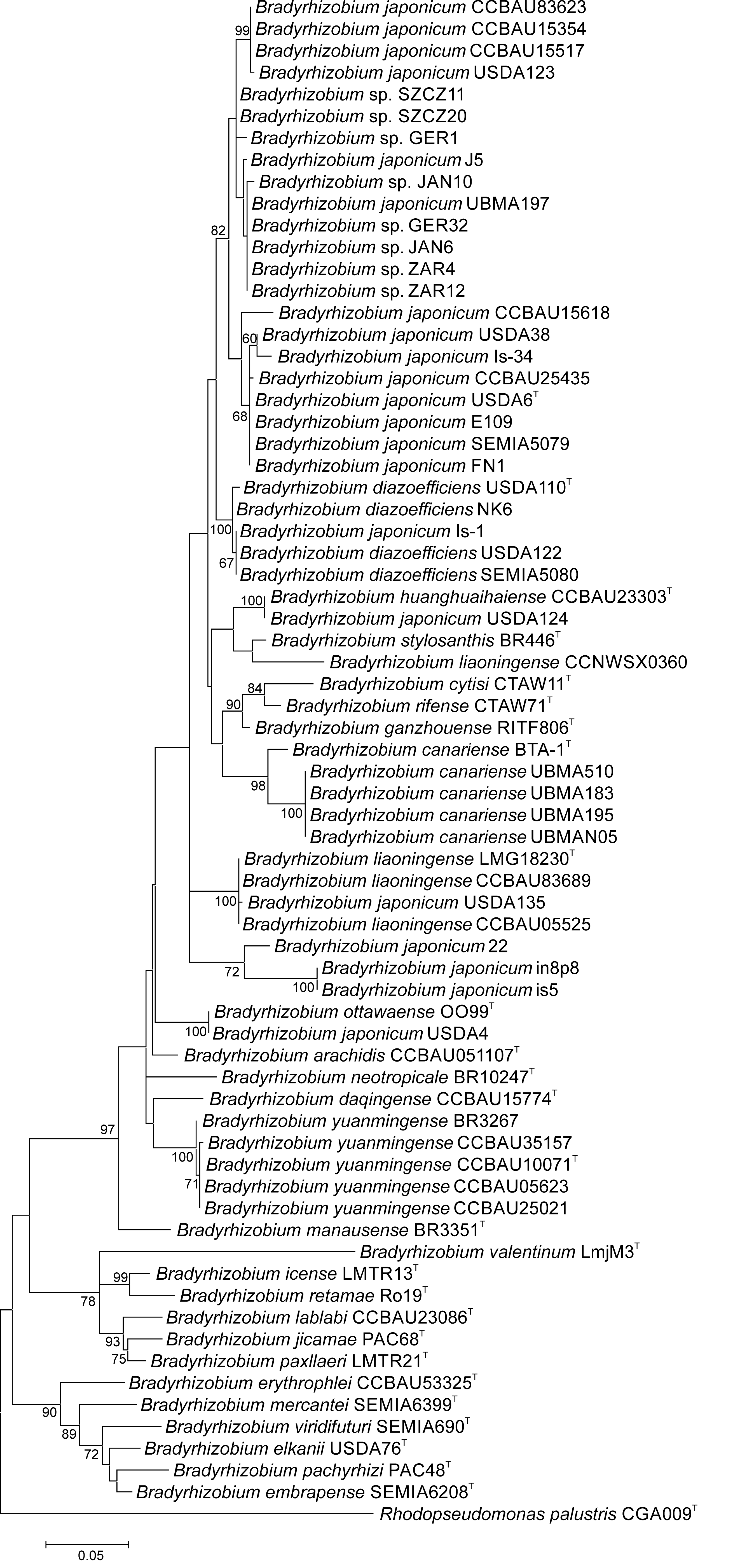

Supplementary Figure S1. Maximum Likelihood (ML) phylogenetic tree of *glnII* gene sequences of *Bradyrhizobium* strains. Bootstrap values ⩾70% are given at branching points. The scale bar indicates the number of substitution per site.

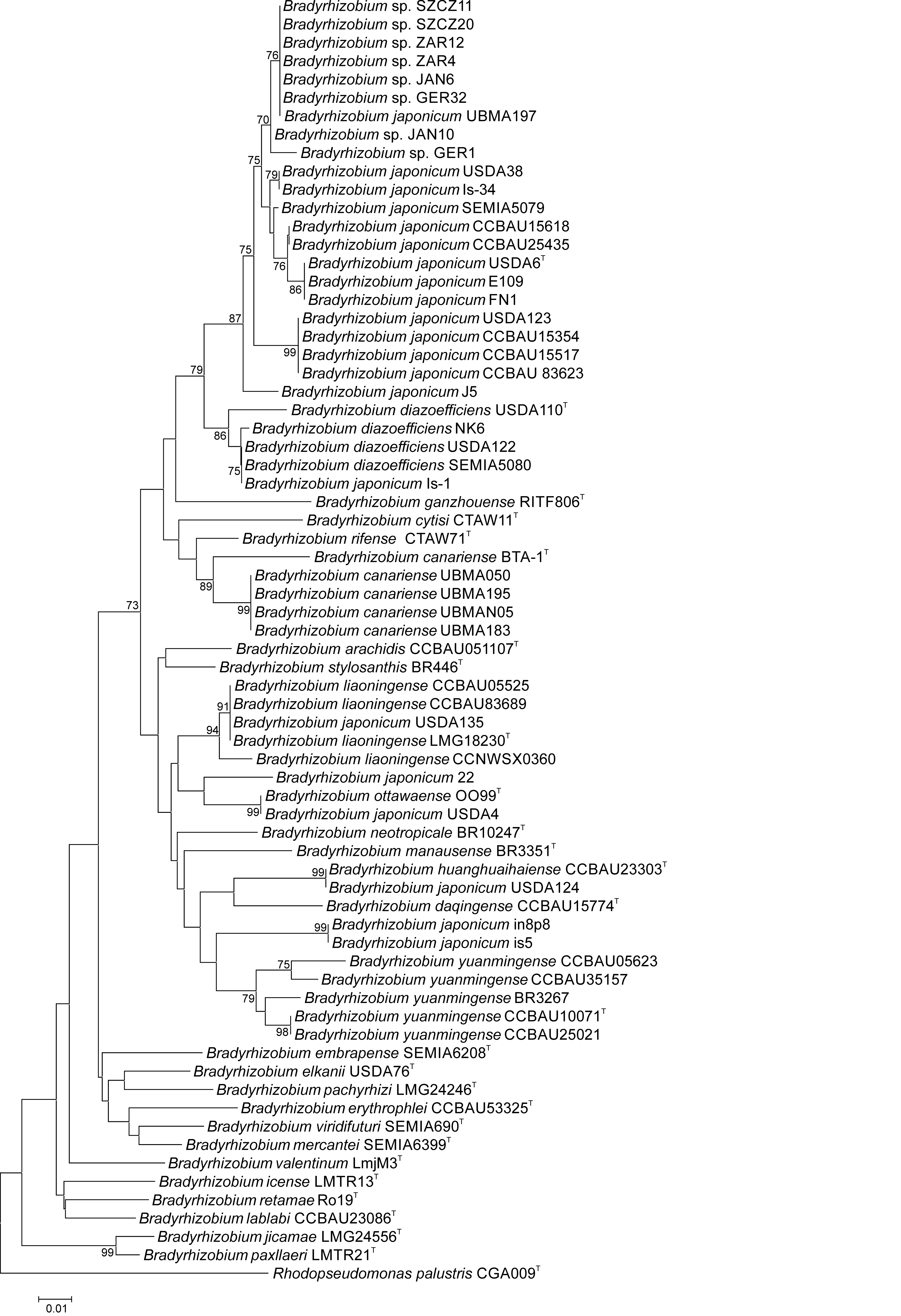

Supplementary Figure S2. Maximum Likelihood (ML) phylogenetic tree of *recA* gene sequences of *Bradyrhizobium* strains. Bootstrap values ⩾70% are given at branching points. The scale bar indicates substitution per site.

```
                                         210        220        230        240        250
                                ....|....| ....|....| ....|....| ....|....| ....|....|
B.japonicum USDA6T              CGACATGAAC GTCGTCACCT GCGAGGCCAC CGTTGCCCGC AACCTGATGC
Bradyrhizobium sp.JAN6          .......... .......... .......... .........T ..........
Bradyrhizobium sp.JAN10         .......... .......... .......... .........T ..T.......
Bradyrhizobium sp.GER1          .......... .......... .......... .........T ..........
Bradyrhizobium sp.GER32         .......... .......... .......... .........T ..........
Bradyrhizobium sp.ZAR4          .......... .......... .......... .........T ..........
Bradyrhizobium sp.ZAR12         .......... .......... .......... .........T ..........
Bradyrhizobium sp.SZCZ11        .......... .......... .......... .........T ..........
Bradyrhizobium sp.SZCZ20        .......... .......... .......... .........T ..........
B.japonicum E109                .......... .......... .......... .........  ..........
B.japonicum J5                  .......... .......... .......... .........T ..........
B.japonicum SEMIA5079           .......... .......... .......... .........  ..........
B.japonicum CCBAU83623          .......... .......... .......... .........T ..........
B.japonicum FN1                 .......... .......... .......... .........  ..........
B.japonicum UBMA197             .......... .......... .......... .........T ..........
B.japonicum USDA38              .......... .......... .......... .........T ..........
B.japonicum USDA123             .......... .......... .......... .........T ..........
B.japonicum CCBAU15354          .......... .......... .......... .........T ..........
B.japonicum CCBAU15517          .......... .......... .......... .........T ..........
B.japonicum CCBAU15618          .......... .......... .......... .........  ..........
B.japonicum CCBAU25435          .......... .......... .......... .........  ..........
B.japonicum Is-34               .......... .......... .......... G........T ..........
B.japonicum BlupMR-1            .......... .......... .......... .........T ..T.......
B.japonicum BGA-1               .......... .......... .......... .........T ..........
Bradyrhizobium sp.G22           .......... .......... .......... .........T ..T.......
Bradyrhizobium sp.CCH1-B1       .......C.T ......G..A ........T. G.CG..G... ...G.C....
Bosea robiniae DSM26672T        .......C.T ......G..A .T.....GG. G.CG..G... ...G.C....
Bradyrhizobium sp.CCH5-A9       .......C.T ......G..A ........T. G.CG..G... ...G.C....
Bosea thiooxidans DSM9653T      .......C.T ......G..A .......GG. G.CG..G... ...G......
Bosea lupini LMG26383T          .......C.T ......G.GG CG..A..GG. G.CG..G... ..TG.C....
Bradyrhizobium sp.CCGE-LA001    .......... .......... ....A..... T..G..G..G ..........
B.japonicum 22                  .......... .......... ....C..... ...G..G..G ..........
B.japonicum USDA135             .......... .......... ....C..... ...G..G..G ..........
B.japonicum USDA4               .......... .......... ....C..... ...G..G..G ..........
B.japonicum IS-1                .......... .......... .T..C..... ...G..G..G ..........
B.japonicum USDA124             .......... ........T. ....T..... ...G..G..G ..........
B.japonicum in8p8               .......... ..G....... ....C..... G..G..G... ..........
B.japonicum is5                 .......... ..G....... ....C..... G..G..G... ..........
Bradyrhizobium sp.WSM471        .......... .......... ....C..... ...G..G..G ..........
Bradyrhizobium sp.ORS285        .........T .........A CG..T..... ...C...AAG ..........
Bradyrhizobium sp.WSM4349       .........T .......... ....C..... G..G..G..G ...T......
Bradyrhizobium sp.WSM2793       .......... .......... ....C..... ...G..G..G ..........
Bradyrhizobium sp.URHA0002      .......... ..G..G..GG CG..C..... ...C...... ..........
Bradyrhizobium sp.WSM1253       .........T ........T. ....C..... G..G..G..G ..........
Bradyrhizobium sp.URHD0069      .......... ..G.....GC TA..T..G.. G..C...AAG ..T.......
Bradyrhizobium sp.URHA0013      .......... .......... ....C..... G..G..G..G ..........
Bradyrhizobium sp.WSM1417       .......... .......... ....C..... ...G..G..G ..........
Bradyrhizobium sp.WSM1743       .......... .......... .T..C..... ...G..G..G ..........
Bradyrhizobium sp.WSM3983       .......... .......... ....C..... ...G..G..G ..........
Bradyrhizobium sp.DOA9          .......... .......... .T..C..... ...G..G..A ..........
Bradyrhizobium sp.Ec3.3         .......... ..G..G.... ....C..... ...G..G..G ..........
Bradyrhizobium sp.USDA3384      .......... .......... ....C..... ...G..G..G ..........
Bradyrhizobium sp.WSM2254       .......... .......... .T..C..... ...G..G..G ..........
Bradyrhizobium sp.th.b2         .........T .....G..G. CG..T..G.. G..C...AAG ..T.......
Bradyrhizobium sp.Cp5.3         .......... .......... ....C..... G..G..G..G ..........
Bradyrhizobium sp.Ai1a-2        .......... ........GG C...C..... ...C...AAG ..........
Bradyrhizobium sp.STM3843       .......... .....G...A C...C..... G......A.G ..........
Bradyrhizobium sp.CCBAU15615    .......... .......... ....C..... ...G..G..G ..........
Bradyrhizobium sp.CCBAU15544    .......... .......... ....C..... ...G..G..G ..........
Bradyrhizobium sp.CCBAU43298    .......... .......... ....C..... ...G..G..G ..T.......
Bradyrhizobium sp.STM3809       .......... .........A CG..T..... ...G...AAG ..........
B.centrolobii BR10245T          .......... .......... ....C..... G..G..G..G ..........
Bradyrhizobium sp.AT1           .......... .......... ....C..... ...A..G..G ..........
Bradyrhizobium sp.DOA1          .......... .......... ....A..... T..G..G..G ..........
B.sacchari BR10280T             .......... .......... ....C..... ...G..G..G ..........
B.macuxiense BR10303T           .......... ..G..G..G. CG..C..... ...C...AAG ..........
B.shewense ERR11T               .......... .....T..T. ....C..... ...G..G..G ..........
Bradyrhizobium sp.Rc2d          .......... .......... ....C..... ...G..A..G ..........
Bradyrhizobium sp.cf659         .......... .......... .T..C..... ...G..A..G ..........
Bradyrhizobium sp.Rc3b          .......... .....T..T. ....C..... ...G..G..G ..........
Bradyrhizobium sp.Ghvi          .......... .......... .T..C..... ...G..G... ..........
Bradyrhizobium sp.R5            .......... ..G..G..G. CG..T..G.. G..C...AAG ..........
Bradyrhizobium sp.Gha           .......... .......... ....C..G.. ...G..A..A ..........
Bradyrhizobium sp.Leaf396       .......... ..G....... ....C..... G..G..G... ..........
Bradyrhizobium sp.LTSP849       .......... ..G....... ....C..... G..G..G..G ..........
Bradyrhizobium sp.LTSP885       .......... ..G.....A. CG..T..G.. G..C...AAG ..........
Bradyrhizobium sp.LTSP857       .......... ..G....... ....C..... G..G..G..G ..........
Bradyrhizobium sp.LTSPM299      .......... ..G.....A. CG..T..G.. G..G...AAG ..........
Bradyrhizobium sp.ORS375        .........T ........GA CG..T..... ...C...AAG ..........
Bradyrhizobium sp.NFR13         ...T.....T ........TG CG..C..G.. G..C..A..T ..........
Bradyrhizobium sp.OK095         .......... .......... ....C..... ...G..G..G ..........
Bradyrhizobium sp.C9            .........T ..G..G..G. CG..T..G.. G..C...AAG ..........
Bradyrhizobium sp.Y36           .......... ........T. ....C..... ...G..G..G ..........
Bradyrhizobium sp.UFLA03-84     .......... ..G..G..G. CG..C..G.. ...C...AAG ..........
Bradyrhizobium sp.85S1MB        .......... .......... ....C..... ...G..G..G ..........
Bradyrhizobium sp.39S1MB        .......... .......... ....C..... ...G..G..G ..........
Bradyrhizobium sp.PARBB1        .......... ..G..G..G. CG..T..G.. G..C...AAG ..........
Bradyrhizobium sp.LMTR3         .......C.A .....G..GG CG..T..... ...C.....T ..........
Bradyrhizobium sp.AS23.2        .......... .......... .T..C..... A..G..A..G ..........
Bradyrhizobium sp.NAS80.1       .........T .......... .T..C..... G..G..G..G ..........
Bradyrhizobium sp.NAS96.2       .......... ..G..G..G. CG..C..G.. G......AAG ..........
B.brasilense UFLA03-321T        .......... ..G..G..G. CG..C..G.. G......AAG .........T
Bradyrhizobium sp.ORS287        .........T .........A CG..T..... ...C...AAG ..........
Bradyrhizobium sp.CCBAU15635    .......... .......... ....C..... ...G..G..G ..........
Bradyrhizobium sp.CCBAU41267    .......... .......... .T..C..... ...G..G..G ..........
Bradyrhizobium sp.YR681_PMI42   .......... .......... ....C..... ...G..G..G ..........
Bradyrhizobium sp.ARR65         ...G.....T ..T..G...G CG..C..G.. ...G..G.A. ..........
Bradyrhizobium sp.CCH5-F6       .......... .......... ....C..... ...G..G..G ..........
Bradyrhizobium sp.CCH4-A6       .......... ..TA.T...A C...C..A.. G.CG..GAAG ..........
Bradyrhizobium sp.OHSUIII       .......... ..G..G.... CG..C..G.. G..C...AAG ...T......
Bradyrhizobium sp.Tv2a-2        T........T .....A...G CG..C..G.. G..G..G..G ..........
B.diazoefficiens USDA122        .......... .......... .T..C..... ...G..G..G ..........
B.diazoefficiens NK6            .......... .......... .T..C..... ...G..G..G ..........
B.diazoefficiens SEMIA5080      .......... .......... .T..C..... ...G..G..G ..........
B.diazoefficiens USDA110T       .......... .......... .T..C..... ...G..G..G ..........
B.yuanmingense CCBAU10071T      .......... .......... ....C..... ...G..G..G ..........
B.yuanmingense BR3267           .......... .......... ....C..... ...G..G..G ..........
B.yuanmingense CCBAU05623       .......... .......... ....C..... ...G..G..G ..........
B.yuanmingense CCBAU25021       .......... .......... ....C..... ...G..G..G ..........
B.yuanmingense CCBAU35157       .......... .......... ....C..... ...G..G..G ..........
B.liaoningense LMG18230T        .......... .......... ....C..... ...G..G..G ..........
B.liaoningense CCBAU05525       .......... .......... ....C..... ...G..G..G ..........
B.liaoningense CCBAU83689       .......... .......... ....C..... ...G..G..G ..........
B.liaoningense CCNWSX0360       .......... .......... ....T..... ...G..G..G ..........
B.canariense BTA-1T             .........T ........T. ....C..... G..G..G..G ..........
B.canariense UBMA050            .........T .......... ....C..... G..G..G..G ..........
B.canariense UBMAN05            .........T .......... ....C..... G..G..G..G ..........
B.canariense UBMA510            .........T .......... ....C..... G..G..G..G ..........
B.canariense UBMA181            .........T .......... ....C..... G..G..G..G ..........
B.canariense BC-C2              .......... .......... ....C..... ...G..G..G ..........
B.canariense BC-P5              .......... .......... ....C..... ...G..G..G ..........
B.canariense GAS369             .......... ........G. CG..C..G.. G..C...AAG ..T.......
B.canariense UBMA183            .........T .......... ....C..... G..G..G..G ..........
B.canariense UBMA192            .........T .......... ....C..... G..G..G..G ..........
B.canariense UBMA182            .........T .......... ....C..... G..G..G..G ..........
B.canariense UBMA171            .........T .......... ....C..... G..G..G..G ..........
B.canariense UBMA122            .........T .......... ....C..... G..G..G..G ..........
B.canariense UBMA195            .........T .......... ....C..... G..G..G..G ..........
B.canariense UBMA061            .........T .......... ....C..... G..G..G..G ..........
B.canariense UBMA052            .........T .......... ....C..... G..G..G..G ..........
B.canariense UBMA060            .........T .......... ....C..... G..G..G..G ..........
B.canariense UBMA051            .........T .......... ....C..... G..G..G..G ..........
B.daqingense CCBAU15774T        .......... .......... ....C..... ...G..G..G ..........
B.arachidis LMG26795T           .......... .......... ....C..... ...G..G..G ..........
B.stylosanthis BR446T           .......... .......... ....C..... G..G..G..G ..........
B.neotropicale BR10247T         .......... .......... ....C..... G..G..G..G ...T......
B.ottawaense OO99T              .......... .......... ....C..... ...G..G..G ..........
B.ottawaense GAS524             .......C.G ..G..G..GG CG..C..... ...C...... ..........
B.huanghuaihaiense CCBAU23303T  .......... ........T. ....T..... ...G..G..G ..........
B.cytisi CTAW11T                .......... ..T....... ....C..... ...G..A..G .....T....
B.rifense CTAW71T               ...T...... .......... ....C..... ...G..G..G ..........
B.manausense BR3351T            .........T .......... ....T..... ...G..G..G ..T.......
B.ganzhouense RITF806T          .......... .......... ....C..... ...G..G..G ..........
B.oligotrophicum S58T           .......... ..G......A C...C..... ...G...AAG ..........
B.pachyrhizi PAC48T             .......... ..G..G..G. CG..C..G.. T..C...AAG ..........
B.pachyrhizi BR3263             T......... ..G..G..G. CG..C..G.. G......AAG .........T
B.valentinum LmjM3T             .......... ..G..G..GG CG..C..G.. .......... ..........
B.valentinum LmjM6              .......... ..G..G..GG CG..C..G.. .......... ..........
B.retamae Ro19T                 .......C.A .....G..GG CG..T..... ...C...... ..........
B.paxllaeri LMTR21T             .......C.G ..G..G..GG TG..T..... G......... ..T.......
B.jicamae PAC68T                .......C.G ..G..G..GG TG..T..... .......... ..T.......
B.embrapense SEMIA6208T         .........T ..G..G..G. CG..C..G.. G..C...AAG ..........
B.icense LMTR13T                .......... ..G..G..GG CG..T..... G..C...... ..TT......
B.mercantei SEMIA6399T          .......... .....G..G. CG..T..G.. G..C...AAG ..........
B.viridifuturi SEMIA690T        .......... ..G..G..G. CG..C..G.. ...C...AAG .........T
B.elkanii USDA76T               .......... ..G..G..G. CG..C..G.. G..C...AAG ..........
B.elkanii WSM2783               .......... ..G.....GG C...C..... ...C...AAG ..........
B.elkanii USDA94                .........T ..G..G..G. CG..C..G.. G..C...AAG ..........
B.elkanii CCBAU05737            .........T ..G..G..G. CG..C..G.. G..C...AAG ..........
B.elkanii WSM1741               .......C.A .....G..GG CG..T..... ...C...... ..........
B.elkanii BLY6-1                .......... ..G..G..G. CG..C..G.. G..C...AAG ..........
B.elkanii TnphoA33              .......... ..G..G..G. CG..C..G.. G..C...AAG ..........
B.elkanii UASW1015              .......... ..G..G.... CG..C..G.. G..C...AAG ...T......
B.elkanii USDA3259              .......... ..G..G..G. CG..C..G.. T..C...AAG ..........
B.elkanii USDA3254              .......... ..G..G..G. CG..C..G.. T..C...AAG ..........
B.elkanii CCBAU43297            .......... ..G..G..G. CG..C..G.. G..C...AAG ..........
B.elkanii BLY3-8                .......... ..G..G..G. CG..C..G.. G..C...AAG ..........
B.erythrophlei GAS478           .......... .........G CG..C..... ...C...AAA ..........
B.erythrophlei GAS138           .......... .........G TG..T..G.. G..C...AAG ..........
B.erythrophlei GAS242           .......... ..G..G.... CG..C..... ...C...AAG ..........
B.erythrophlei GAS401           ...T...... ..G.....G. CG..TA.G.. ...G.T.AAA ..........
B.erythrophlei MT12             .........T ..G..G..G. CG..C..G.. G......AAG ..........
B.lablabi GAS522                .......C.G ..G..G..GG CG..C..... ...C...... ..........
B.lablabi CCBAU23086T           .......C.G ..G..G..GG TG..T..... .......... ..T.......
B.lablabi GAS499                .......... .......... CG..T..... G......AAG ..T.......
B.lablabi MT34                  .......C.G ..G..G..GG CG..C..... ...C...... ..........
```

Supplementary Figure S3. Nucleotide sequence alignment of the *ftsA* gene fragments of 176 bacterial strains used in the study. Only differences relative to the top sequence (*Bradyrhizobium japonicum* USDA6^{T}) are shown. The shaded nucleotide position 225 corresponds to the single nucleotide polymorphism (guanine) unique to *Bradyrhizobium japonicum*.

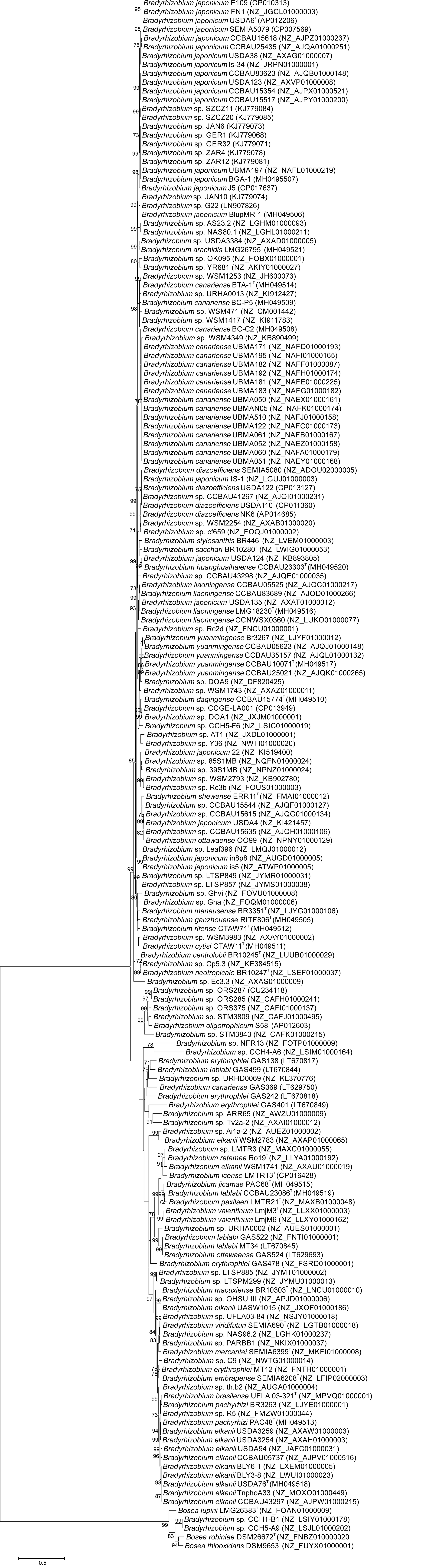

Supplementary Figure S4. Maximum Likelihood (ML) phylogenetic tree of *ftsA* gene sequences of 176 *Bradyrhizobium* and *Bosea* strains. Bootstrap values ⩾70% are given at branching points. The scale bar indicates the number of substitution per site.

Supplementary Table S1. Bacterial strains and GenBank or RefSeq accession numbers of nucleotide sequences used in the study. The GenBank accession numbers of the sequences generated during this study are given in boldface.

**Bacterial strain**	***ftsA***	***glnII***	***recA***
B. japonicum USDA 6^{T}	AP012206	AP012206	AM182158
Bradyrhizobium sp. JAN6	**KJ779073**	KJ778987	KJ779019
Bradyrhizobium sp. JAN10	**KJ779074**	KJ778988	KJ779020
Bradyrhizobium sp.GER1	**KJ779068**	KJ778982	KJ779014
Bradyrhizobium sp. GER32	**KJ779071**	KJ778985	KJ779017
Bradyrhizobium sp. ZAR4	**KJ779078**	KJ778990	KJ779022
Bradyrhizobium sp. ZAR12	**KJ779081**	KJ778993	KJ779025
Bradyrhizobium sp. SZCZ11	**KJ779084**	KJ778996	KJ779028
Bradyrhizobium sp. SZCZ20	**KJ779085**	KJ778997	KJ779029
B. japonicum E109	CP010313	CP010313	CP010313
B. japonicum J5	CP017637	CP017637	CP017637
B. japonicum SEMIA 5079	CP007569	CP007569	CP007569
B. japonicum CCBAU 83623	NZ_AJQB01000148	NZ_AJQB01000261	NZ_AJQB01000058
B. japonicum FN1	NZ_JGCL01000003	NZ_JGCL01000004	NZ_JGCL01000027
B. japonicum UBMA 197	NZ_NAFL01000219	NZ_NAFL01000199	NZ_NAFL01000280
B. japonicum USDA 38	NZ_AXAG01000007	NZ_AXAG01000013	NZ_AXAG01000016
B. japonicum USDA 123	NZ_AXVP01000008	NZ_AXVP01000009	NZ_AXVP01000114
B. japonicum CCBAU 15354	NZ_AJPX01000521	NZ_AJPX01000267	NZ_AJPX01000311
B. japonicum CCBAU 15517	NZ_AJPY01000200	NZ_AJPY01000305	NZ_AJPY01000121
B. japonicum CCBAU 15618	NZ_AJPZ01000237	NZ_AJPZ01000275	NZ_AJPZ01000151
B. japonicum CCBAU 25435	NZ_AJQA01000251	NZ_AJQA01000044	NZ_AJQA01000199
B. japonicum Is-34	NZ_JRPN01000001	NZ_JRPN01000020	NZ_JRPN01000005
B. japonicum Blup-MR1	**MH049506**	AY386774	AY591559
B. japonicum BGA-1	**MH049507**	AY386772	AY591558
Bradyrhizobium sp. G22	LN907826	LN907826	LN907826
Bradyrhizobium sp. CCH1-B1	NZ_LSIY01000178	-	-
Bosea robiniae DSM 26672^{T}	NZ_FNBZ01000002	-	-
Bradyrhizobium sp. CCH5-A9	NZ_LSJL01000202	-	-
Bosea thiooxidans DSM 9653^{T}	NZ_FUYX01000001	-	-
Bosea lupini LMG 26383^{T}	NZ_FOAN01000009	-	-
Bradyrhizobium sp. CCGE-LA001	CP013949	CP013949	CP013949
B. japonicum 22	NZ_KI519400	NZ_KI519399	NZ_KI519399
B. japonicum USDA 135	NZ_AXAT01000012	NZ_AXAT01000317	NZ_AXAT01000062
B. japonicum USDA 4	NZ_KI421457	NZ_KI421456	NZ_AXAF01000018
B. japonicum IS-1	NZ_LGUJ01000003	NZ_LGUJ01000004	NZ_LGUJ01000012
B. japonicum USDA 124	NZ_KB893805	NZ_KB893842	NZ_KB893818
B. japonicum in8p8	NZ_AUGD01000005	NZ_AUGD01000002	NZ_AUGD01000009
B. japonicum is5	NZ_ATWP01000005	NZ_ATWP01000002	NZ_ATWP01000009
Bradyrhizobium sp. WSM 471	NZ_CM001442	-	-
Bradyrhizobium sp. ORS 285	NZ_CAFH01000241	-	-
Bradyrhizobium sp. WSM 4349	NZ_KB890499	-	-
Bradyrhizobium sp. WSM 2793	NZ_KB902780	-	-
Bradyrhizobium sp. URHA0002	NZ_AUES01000001	-	-
Bradyrhizobium sp. WSM 1253	NZ_JH600073	-	-

Supplementary Table S1. Continued

Bradyrhizobium sp. URHD0069	NZ_KL370776	-	-
Bradyrhizobium sp. URHA0013	NZ_KI912427	-	-
Bradyrhizobium sp. WSM 1417	NZ_KI911783	-	-
Bradyrhizobium sp. WSM 1743	NZ_AXAZ01000011	-	-
Bradyrhizobium sp. WSM 3983	NZ_AXAY01000002	-	-
Bradyrhizobium sp. DOA9	NZ_DF820425	-	-
Bradyrhizobium sp. Ec3.3	NZ_AXAS01000009	-	-
Bradyrhizobium sp. USDA 3384	NZ_AXAD01000005	-	-
Bradyrhizobium sp. WSM 2254	NZ_AXAB01000020	-	-
Bradyrhizobium sp. th.b2	NZ_AUGA01000004	-	-
Bradyrhizobium sp. Cp5.3	NZ_KE384515	-	-
Bradyrhizobium sp. Ai1a-2	NZ_AUEZ01000002	-	-
Bradyrhizobium sp. STM3843	NZ_CAFK01000215	-	-
Bradyrhizobium sp. CCBAU 15615	NZ_AJQG01000134	-	-
Bradyrhizobium sp. CCBAU 15544	NZ_AJQF01000127	-	-
Bradyrhizobium sp. CCBAU 43298	NZ_AJQE01000035	-	-
Bradyrhizobium sp. STM3809	NZ_CAFJ01000495	-	-
Bradyrhizobium sp. BR 10245	NZ_LUUB01000029	-	-
Bradyrhizobium sp. AT1	NZ_JXDL01000001	-	-
Bradyrhizobium sp. DOA1	NZ_JXJM01000001	-	-
Bradyrhizobium sp. BR 10280	NZ_LWIG01000053	-	-
Bradyrhizobium sp. BR 10303	NZ_LNCU01000010	-	-
Bradyrhizobium sp. err11	NZ_FMAI01000012	-	-
Bradyrhizobium sp. Rc2d	NZ_FNCU01000001	-	-
Bradyrhizobium sp. cf659	NZ_FOQJ01000002	-	-
Bradyrhizobium sp. Rc3b	NZ_FOUS01000003	-	-
Bradyrhizobium sp. Ghvi	NZ_FOVU01000008	-	-
Bradyrhizobium sp. R5	NZ_FMZW01000044	-	-
Bradyrhizobium sp. Gha	NZ_FOQM01000006	-	-
Bradyrhizobium sp. Leaf396	NZ_LMQJ01000012	-	-
Bradyrhizobium sp. LTSP849	NZ_JYMR01000031	-	-
Bradyrhizobium sp. LTSP885	NZ_JYMT01000002	-	-
Bradyrhizobium sp. LTSP857	NZ_JYMS01000038	-	-
Bradyrhizobium sp. LTSPM299	NZ_JYMU01000013	-	-
Bradyrhizobium sp. ORS375	NZ_CAFI01000137	-	-
Bradyrhizobium sp. NFR13	NZ_FOTP01000009	-	-
Bradyrhizobium sp. OK095	NZ_FOBX01000001	-	-
Bradyrhizobium sp. C9	NZ_NWTG01000014	-	-
Bradyrhizobium sp. Y36	NZ_NWTI01000020	-	-
Bradyrhizobium sp. UFLA03-84	NZ_NSJY01000018	-	-
Bradyrhizobium sp. 85S1MB	NZ_NQFN01000024	-	-
Bradyrhizobium sp. 39S1MB	NZ_NPNZ01000024	-	-
Bradyrhizobium sp. PARBB1	NZ_NKIX01000037	-	-
Bradyrhizobium sp. LMTR3	NZ_MAXC01000055	-	-

Supplementary Table S1. Continued

Bradyrhizobium sp. AS23.2	NZ_LGHM01000093	-	-
Bradyrhizobium sp. NAS80.1	NZ_LGHL01000211	-	-
Bradyrhizobium sp. NAS96.2	NZ_LGHK01000237	-	-
Bradyrhizobium sp. UFLA03-321	NZ_MPVQ01000001	-	-
Bradyrhizobium sp. ORS 287	CU234118	-	-
Bradyrhizobium sp. CCBAU 15635	NZ_AJQH01000106	-	-
Bradyrhizobium sp. CCBAU 41267	NZ_AJQI01000231	-	-
Bradyrhizobium sp. YR681_PMI42	NZ_AKIY01000027	-	-
Bradyrhizobium sp. ARR65	NZ_AWZU01000009	-	-
Bradyrhizobium sp. CCH5-F6	NZ_LSIC01000019	-	-
Bradyrhizobium sp. CCH4-A6	NZ_LSIM01000164	-	-
Bradyrhizobium sp. OHSUIII	NZ_APJD01000006	-	-
Bradyrhizobium sp. Tv2a-2	NZ_AXAI01000012	-	-
B. diazoefficiens USDA 122	CP013127	CP013127	CP013127
B. diazoefficiens NK6	AP014685	AP014685	AP014685
B. diazoefficiens SEMIA 5080	NZ_ADOU02000005	FJ391037	FJ391157
B. diazoefficiens USDA 110^T	CP011360	CP011360	CP011360
B. yuanmingense CCBAU 10071^T	**MH049517**	AY386780	AM168343
B. yuanmingense BR 3267	NZ_LJYF01000012	NZ_LJYF01000031	NZ_LJYF01000050
B. yuanmingense CCBAU 05623	NZ_AJQJ01000148	NZ_AJQJ01000020	NZ_AJQJ01000383
B. yuanmingense CCBAU 25021	NZ_AJQK01000265	NZ_AJQK01000073	NZ_AJQK01000242
B. yuanmingense CCBAU 35157	NZ_AJQL01000132	NZ_AJQL01000076	NZ_AJQL01000345
B. liaoningense LMG 18230^T	**MH049516**	AY386775	AY591564
B. liaoningense CCBAU 05525	NZ_AJQC01000217	NZ_AJQC01000002	NZ_AJQC01000209
B. liaoningense CCBAU 83689	NZ_AJQD01000266	NZ_AJQD01000184	NZ_AJQD01000224
B. liaoningense CCNWSX0360	NZ_LUKO01000077	NZ_LUKO01000011	NZ_LUKO01000219
B. canariense BTA-1^T	**MH049514**	AY386765	FM253177
B. canariense UBMA050	NZ_NAEX01000161	NZ_NAEX01000187	NZ_NAEX01000165
B. canariense UBMAN05	NZ_NAFK01000174	NZ_NAFK01000166	NZ_NAFK01000156
B. canariense UBMA510	NZ_NAFJ01000158	NZ_NAFJ01000143	NZ_NAFJ01000142
B. canariense UBMA181	NZ_NAFE01000225	NZ_NAFE01000226	NZ_NAFE01000205
B. canariense BC-C2	**MH049508**	AY386762	AY591541
B. canariense BC-P5	**MH049509**	AY386763	AY591542
B. canariense GAS369	LT629750	LT629750	LT629750
B. canariense UBMA183	NZ_NAFG01000182	NZ_NAFG01000156	NZ_NAFG01000159
B. canariense UBMA192	NZ_NAFH01000174	NZ_NAFH01000179	NZ_NAFH01000159
B. canariense UBMA182	NZ_NAFF01000087	NZ_NAFF01000114	NZ_NAFF01000093
B. canariense UBMA171	NZ_NAFD01000193	NZ_NAFD01000194	NZ_NAFD01000173
B. canariense UBMA122	NZ_NAFC01000173	NZ_NAFC01000101	NZ_NAFC01000177
B. canariense UBMA195	NZ_NAFI01000165	NZ_NAFI01000160	NZ_NAFI01000168
B. canariense UBMA061	NZ_NAFB01000167	NZ_NAFB01000196	NZ_NAFB01000170

Supplementary Table S1. Continued

B. canariense UBMA052	NZ_NAEZ01000158	NZ_NAEZ01000182	NZ_NAEZ01000155
B. canariense UBMA060	NZ_NAFA01000179	NZ_NAFA01000204	NZ_NAFA01000183
B. canariense UBMA051	NZ_NAEY01000168	NZ_NAEY01000173	NZ_NAEY01000147
B. daqingense CCBAU 15774^T	**MH049510**	HQ231301	HQ231270
B. arachidis LMG 26795^T	**MH049521**	HM107251	HM107233
B. stylosanthis BR 446^T	NZ_LVEM01000003	KU724148	KU724163
B. neotropicale BR 10247^T	NZ_LSEF01000037	KJ661700	KJ661714
B. ottawaense OO99^T	NZ_NPNY01000129	HQ587750	HQ587287
B. ottawaense GAS524	LT629693	LT629693	LT629693
B. huanghuaihaiense CCBAU 23303^T	**MH049520**	HQ231639	HQ231595
B. cytisi CTAW11^T	**MH049511**	GU001594	GU001575
B. rifense CTAW71^T	**MH049512**	GU001604	GU001585
B. manausense BR 3351^T	NZ_LJYG01000106	KF785986	KF785992
B. ganzhouense RITF 806^T	**MH049505**	JX277110	JX277144
B. oligotrophicum S58^T	AP012603	JQ619233	JQ619231
B. pachyrhizi PAC48^T	**MH049513**	FJ428201	HM047130
B. pachyrhizi BR 3263	NZ_LJYE01000001	NZ_LJYE01000104	NZ_LJYE01000083
B. valentinum LmjM3^T	NZ_LLXX01000003	JX518575	JX518589
B. valentinum LmjM6	NZ_LLXY01000162	NZ_LLXY01000165	NZ_LLXY01000163
B. retamae Ro19^T	NZ_LLYA01000192	KC247108	KF962711
B. paxllaeri LMTR21^T	NZ_MAXB01000048	KF896169	JX943617
B. jicamae PAC68^T	**MH049515**	FJ428204	HM047133
B. embrapense SEMIA 6208^T	NZ_LFIP02000003	GQ160500	HQ634899
B. icense LMTR13^T	CP016428	KF896175	JX943615
B. mercantei SEMIA 6399^T	NZ_MKFI01000008	NZ_MKFI01000002	NZ_MKFI01000001
B. viridifuturi SEMIA 690^T	NZ_LGTB01000018	KR149131	KR149140
B. elkanii USDA 76^T	**MH049518**	AY599117	AY591568
B. elkanii WSM 2783	NZ_AXAP01000065	NZ_AXAP01000034	NZ_AXAP01000011
B. elkanii USDA 94	NZ_JAFC01000031	NZ_KI912603	NZ_JAFC01000011
B. elkanii CCBAU 05737	NZ_AJPV01000516	NZ_AJPV01000153	NZ_AJPV01000320
B. elkanii WSM 1741	NZ_AXAU01000019	NZ_AXAU01000001	NZ_AXAU01000016
B. elkanii BLY6-1	NZ_LXEM01000005	NZ_LXEM01000021	NZ_LXEM01000041
B. elkanii TnphoA33	NZ_MOXO01000449	NZ_MOXO01000222	NZ_MOXO01000196
B. elkanii UASW1015	NZ_JXOF01000186	NZ_JXOF01000183	NZ_JXOF01000179
B. elkanii USDA 3259	NZ_AXAW01000003	NZ_AXAW01000002	NZ_AXAW01000049
B. elkanii USDA 3254	NZ_AXAH01000003	NZ_KI421460	NZ_AXAH01000049
B. elkanii CCBAU 43297	NZ_AJPW01000215	NZ_AJPW01000363	NZ_AJPW01000502
B. elkanii BLY3-8	NZ_LWUI01000023	NZ_LWUI01000019	NZ_LWUI01000007
B. erythrophlei GAS478	NZ_FSRD01000001	NZ_FSRD01000001	NZ_FSRD01000001
B. erythrophlei GAS138	LT670817	LT670817	LT670817
B. erythrophlei GAS242	LT670818	LT670818	LT670818
B. erythrophlei GAS401	LT670849	LT670849	LT670849
B. erythrophlei MT12	NZ_FNTH01000001	NZ_FNTH01000001	NZ_FNTH01000001
B. lablabi GAS522	NZ_FNTI01000001	NZ_FNTI01000001	NZ_FNTI01000001
B. lablabi CCBAU 23086^T	**MH049519**	GU433498	GU433522
B. lablabi GAS499	LT670844	LT670844	LT670844
B. lablabi MT34	LT670845	LT670845	LT670845

Supplementary Table S2. *ftsA* gene sequence similarity between strains used in the study

	Bradyrhizobium sp. JAN6	*Bradyrhizobium* sp. JAN10	*Bradyrhizobium* sp. GER1	*Bradyrhizobium* sp. GER32	*Bradyrhizobium* sp. ZAR4	*Bradyrhizobium* sp. ZAR12	*Bradyrhizobium* sp. SZCZ11	*Bradyrhizobium* sp. SZCZ20	*B. japonicum* USDA6	*B. japonicum* UBMA197	*B. japonicum* CCBAU83623	*B. japonicum* J5	*B. japonicum* E109	*B. japonicum* SEMIA5079	*B. japonicum* FN1	*B. japonicum* Is-34	*B. japonicum* CCBAU25435	*B. japonicum* CCBAU15618	*B. japonicum* CCBAU15517	*B. japonicum* CCBAU15354	*B. japonicum* USDA38	*B. japonicum* USDA123	*B. diazoefficiens* USDA122	*B. diazoefficiens* NK6	*B. diazoefficiens* SEMIA5080	*B. diazoefficiens* USDA110	*B. japonicum* IS-1	*B. japonicum* USDA124	*B. huanghuaihaiense* CCBAU23303	*B. japonicum* in8p8	*B. japonicum* is5	*B. japonicum* 22	*B. yuanmingense* CCBAU10071	*B. yuanmingense* BR3267	*B. yuanmingense* CCBAU05623	*B. yuanmingense* CCBAU25021	*B. yuanmingense* CCBAU35157	*B. canariense* BTA-1	*B. canariense* UBMAN05	*B. canariense* UBMA510	*B. canariense* UBMA183	*B. canariense* UBMA195	*B. liaoningense* LMG18230	*B. liaoningense* CCBAU05525	*B. liaoningense* CCBAU83689	*B. liaoningense* CCNWSX0360	*B. japonicum* USDA135	*B. japonicum* USDA4	*B. ottawaense* OO99	*B. daqingense* CCBAU15774	*B. cytisi* CTAW11	*B. rifense* CTAW71	*B. arachidis* LMG26795	*B. ganzhouense* RITF806	*B. manausense* BR3351	*B. stylosanthis* BR446	*B. neotropicale* BR10247	*B. lablabi* CCBAU23086	*B. pachyrhizi* PAC48	*B. valentinum* LmjM3	*B. elkanii* USDA76	*B. retamae* Ro19	*B. erythrophlei* GAS478	*B. paxllaeri* LMTR21	*B. jicamae* PAC68	*B. embrapense* SEMIA6208	*B. icense* LMTR13	*B. mercantei* SEMIA6399	*B. viridifuturi* SEMIA690	*Rhodopseudomonas palustris* CGA009
Bradyrhizobium sp. JAN6	ID	97,9%	100,0%	100,0%	100,0%	100,0%	98,9%	98,9%	97,6%	100,0%	98,3%	98,4%	97,6%	97,8%	97,6%	98,3%	97,4%	97,4%	98,3%	98,3%	98,3%	98,3%	94,5%	94,6%	94,5%	94,4%	94,5%	93,9%	93,9%	94,5%	94,5%	93,9%	93,1%	93,5%	93,4%	93,1%	93,5%	94,6%	93,9%	93,9%	93,7%	93,8%	95,3%	95,3%	95,3%	95,7%	95,4%	92,9%	93,0%	94,2%	93,1%	93,9%	95,9%	94,1%	94,1%	93,9%	91,2%	80,7%	81,8%	80,3%	82,4%	80,9%	81,3%	81,4%	81,4%	82,6%	80,3%	82,5%	82,5%	80,4%
Bradyrhizobium sp. JAN10	97,9%	ID	97,9%	97,9%	97,9%	97,9%	98,2%	98,2%	97,5%	97,9%	97,6%	98,0%	97,5%	97,4%	97,5%	97,9%	97,2%	96,9%	97,6%	97,6%	97,9%	97,6%	95,3%	95,3%	95,3%	95,2%	95,3%	94,1%	94,1%	94,5%	94,5%	94,5%	93,5%	93,7%	93,5%	93,5%	93,8%	94,6%	94,2%	94,2%	93,9%	94,1%	95,7%	95,7%	95,7%	96,1%	95,9%	93,5%	93,7%	94,5%	93,1%	94,1%	96,3%	94,2%	94,2%	94,8%	91,2%	81,1%	81,8%	80,7%	82,2%	80,7%	81,3%	81,5%	81,9%	82,4%	79,9%	82,2%	82,2%	80,2%
Bradyrhizobium sp. GER1	100,0%	97,9%	ID	100,0%	100,0%	100,0%	98,9%	98,9%	97,6%	100,0%	98,3%	98,4%	97,6%	97,8%	97,6%	98,3%	97,4%	97,4%	98,3%	98,3%	98,3%	98,3%	94,5%	94,6%	94,5%	94,4%	94,5%	93,9%	93,9%	94,5%	94,5%	93,9%	93,1%	93,5%	93,4%	93,1%	93,5%	94,6%	93,9%	93,9%	93,7%	93,8%	95,3%	95,3%	95,3%	95,7%	95,4%	92,9%	93,0%	94,2%	93,1%	93,9%	95,9%	94,1%	94,1%	93,9%	91,2%	80,7%	81,8%	80,3%	82,4%	80,9%	81,3%	81,4%	81,4%	82,6%	80,3%	82,5%	82,5%	80,4%
Bradyrhizobium sp. GER32	100,0%	97,9%	100,0%	ID	100,0%	100,0%	98,9%	98,9%	97,6%	100,0%	98,3%	98,4%	97,6%	97,8%	97,6%	98,3%	97,4%	97,4%	98,3%	98,3%	98,3%	98,3%	94,5%	94,6%	94,5%	94,4%	94,5%	93,9%	93,9%	94,5%	94,5%	93,9%	93,1%	93,5%	93,4%	93,1%	93,5%	94,6%	93,9%	93,9%	93,7%	93,8%	95,3%	95,3%	95,3%	95,7%	95,4%	92,9%	93,0%	94,2%	93,1%	93,9%	95,9%	94,1%	94,1%	93,9%	91,2%	80,7%	81,8%	80,3%	82,4%	80,9%	81,3%	81,4%	81,4%	82,6%	80,3%	82,5%	82,5%	80,4%
Bradyrhizobium sp. ZAR4	100,0%	97,9%	100,0%	100,0%	ID	100,0%	98,9%	98,9%	97,6%	100,0%	98,3%	98,4%	97,6%	97,8%	97,6%	98,3%	97,4%	97,4%	98,3%	98,3%	98,3%	98,3%	94,5%	94,6%	94,5%	94,4%	94,5%	93,9%	93,9%	94,5%	94,5%	93,9%	93,1%	93,5%	93,4%	93,1%	93,5%	94,6%	93,9%	93,9%	93,7%	93,8%	95,3%	95,3%	95,3%	95,7%	95,4%	92,9%	93,0%	94,2%	93,1%	93,9%	95,9%	94,1%	94,1%	93,9%	91,2%	80,7%	81,8%	80,3%	82,4%	80,9%	81,3%	81,4%	81,4%	82,6%	80,3%	82,5%	82,5%	80,4%
Bradyrhizobium sp. ZAR12	100,0%	97,9%	100,0%	100,0%	100,0%	ID	98,9%	98,9%	97,6%	100,0%	98,3%	98,4%	97,6%	97,8%	97,6%	98,3%	97,4%	97,4%	98,3%	98,3%	98,3%	98,3%	94,5%	94,6%	94,5%	94,4%	94,5%	93,9%	93,9%	94,5%	94,5%	93,9%	93,1%	93,5%	93,4%	93,1%	93,5%	94,6%	93,9%	93,9%	93,7%	93,8%	95,3%	95,3%	95,3%	95,7%	95,4%	92,9%	93,0%	94,2%	93,1%	93,9%	95,9%	94,1%	94,1%	93,9%	91,2%	80,7%	81,8%	80,3%	82,4%	80,9%	81,3%	81,4%	81,4%	82,6%	80,3%	82,5%	82,5%	80,4%
Bradyrhizobium sp. SZCZ11	98,9%	98,2%	98,9%	98,9%	98,9%	98,9%	ID	100,0%	98,2%	98,9%	98,7%	98,7%	98,2%	97,8%	98,2%	98,6%	97,6%	97,4%	98,7%	98,7%	98,6%	98,7%	94,9%	94,9%	94,9%	94,8%	94,9%	94,2%	94,2%	94,8%	94,8%	94,4%	93,4%	93,8%	93,8%	93,4%	93,8%	94,8%	94,4%	94,4%	94,1%	94,2%	95,6%	95,6%	95,6%	96,0%	95,7%	93,3%	93,4%	94,5%	93,4%	94,5%	96,1%	94,8%	94,4%	94,5%	91,4%	80,9%	81,9%	80,4%	82,2%	81,0%	81,1%	81,5%	81,8%	83,0%	80,4%	82,6%	82,1%	81,0%
Bradyrhizobium sp. SZCZ20	98,9%	98,2%	98,9%	98,9%	98,9%	98,9%	100,0%	ID	98,2%	98,9%	98,7%	98,7%	98,2%	97,8%	98,2%	98,6%	97,6%	97,4%	98,7%	98,7%	98,6%	98,7%	94,9%	94,9%	94,9%	94,8%	94,9%	94,2%	94,2%	94,8%	94,8%	94,4%	93,4%	93,8%	93,8%	93,4%	93,8%	94,8%	94,4%	94,4%	94,1%	94,2%	95,6%	95,6%	95,6%	96,0%	95,7%	93,3%	93,4%	94,5%	93,4%	94,5%	96,1%	94,8%	94,4%	94,5%	91,4%	80,9%	81,9%	80,4%	82,2%	81,0%	81,1%	81,5%	81,8%	83,0%	80,4%	82,6%	82,1%	81,0%
B. japonicum USDA6	97,6%	97,5%	97,6%	97,6%	97,6%	97,6%	98,2%	98,2%	ID	97,6%	98,0%	98,0%	100,0%	99,5%	100,0%	98,7%	99,1%	98,9%	98,0%	98,0%	98,7%	98,0%	95,0%	94,9%	95,0%	94,9%	95,0%	93,9%	93,9%	94,6%	94,6%	94,2%	93,3%	93,8%	93,8%	93,3%	93,8%	94,1%	93,7%	93,7%	93,4%	93,5%	95,6%	95,6%	95,6%	96,0%	95,7%	93,3%	93,4%	94,4%	93,1%	94,1%	96,1%	94,4%	93,9%	94,6%	91,2%	80,9%	82,1%	80,7%	82,2%	81,3%	81,5%	81,3%	82,1%	82,8%	80,2%	82,4%	82,1%	81,1%
B. japonicum UBMA197	100,0%	97,9%	100,0%	100,0%	100,0%	100,0%	98,9%	98,9%	97,6%	ID	98,3%	98,4%	97,6%	97,8%	97,6%	98,3%	97,4%	97,4%	98,3%	98,3%	98,3%	98,3%	94,5%	94,6%	94,5%	94,4%	94,5%	93,9%	93,9%	94,5%	94,5%	93,9%	93,1%	93,5%	93,4%	93,1%	93,5%	94,6%	93,9%	93,9%	93,7%	93,8%	95,3%	95,3%	95,3%	95,7%	95,4%	92,9%	93,0%	94,2%	93,1%	93,9%	95,9%	94,1%	94,1%	93,9%	91,2%	80,7%	81,8%	80,3%	82,4%	80,9%	81,3%	81,4%	81,4%	82,6%	80,3%	82,5%	82,5%	80,4%
B. japonicum CCBAU83623	98,3%	97,6%	98,3%	98,3%	98,3%	98,3%	98,7%	98,7%	98,0%	98,3%	ID	98,4%	98,0%	97,8%	98,0%	98,6%	97,6%	97,4%	100,0%	100,0%	98,6%	100,0%	94,8%	94,9%	94,8%	94,6%	94,8%	94,2%	94,2%	94,9%	94,9%	94,6%	93,4%	93,8%	93,9%	93,4%	93,8%	94,4%	93,9%	93,9%	93,7%	93,8%	95,6%	95,6%	95,6%	96,0%	95,7%	93,7%	93,8%	94,6%	93,4%	94,2%	96,1%	94,6%	94,4%	94,5%	91,4%	81,0%	81,8%	80,7%	82,1%	81,1%	81,1%	81,5%	81,9%	82,8%	80,4%	82,2%	82,2%	80,9%
B. japonicum J5	98,4%	98,0%	98,4%	98,4%	98,4%	98,4%	98,7%	98,7%	98,0%	98,4%	98,4%	ID	98,0%	97,9%	98,0%	98,4%	97,8%	97,5%	98,4%	98,4%	98,4%	98,4%	95,2%	95,3%	95,2%	95,0%	95,2%	94,6%	94,6%	95,4%	95,4%	94,5%	93,5%	93,9%	93,8%	93,5%	93,9%	94,8%	94,4%	94,4%	94,1%	94,2%	96,0%	96,0%	96,0%	96,4%	96,1%	93,7%	93,8%	94,9%	93,4%	94,4%	96,3%	94,5%	94,5%	95,2%	91,9%	81,0%	82,6%	80,7%	82,6%	81,4%	81,5%	81,3%	81,8%	83,4%	80,4%	83,0%	82,5%	81,3%
B. japonicum E109	97,6%	97,5%	97,6%	97,6%	97,6%	97,6%	98,2%	98,2%	100,0%	97,6%	98,0%	98,0%	ID	99,5%	100,0%	98,7%	99,1%	98,9%	98,0%	98,0%	98,7%	98,0%	95,0%	94,9%	95,0%	94,9%	95,0%	93,9%	93,9%	94,6%	94,6%	94,2%	93,3%	93,8%	93,8%	93,3%	93,8%	94,1%	93,7%	93,7%	93,4%	93,5%	95,6%	95,6%	95,6%	96,0%	95,7%	93,3%	93,4%	94,4%	93,1%	94,1%	96,1%	94,4%	93,9%	94,6%	91,2%	80,9%	82,1%	80,7%	82,2%	81,3%	81,5%	81,3%	82,1%	82,8%	80,2%	82,4%	82,1%	81,1%
B. japonicum SEMIA5079	97,8%	97,4%	97,8%	97,8%	97,8%	97,8%	97,8%	97,8%	99,5%	97,8%	97,8%	97,9%	99,5%	ID	99,5%	98,6%	99,0%	99,3%	97,8%	97,8%	98,6%	97,8%	94,9%	94,8%	94,9%	94,8%	94,9%	93,8%	93,8%	94,5%	94,5%	93,9%	93,1%	93,7%	93,5%	93,1%	93,7%	93,9%	93,5%	93,5%	93,3%	93,4%	95,4%	95,4%	95,4%	96,1%	95,6%	93,0%	93,1%	94,2%	93,0%	93,9%	96,3%	94,1%	93,8%	94,4%	90,9%	80,9%	81,9%	80,4%	82,1%	81,3%	81,4%	81,3%	82,1%	82,4%	80,2%	82,2%	81,9%	80,7%
B. japonicum FN1	97,6%	97,5%	97,6%	97,6%	97,6%	97,6%	98,2%	98,2%	100,0%	97,6%	98,0%	98,0%	100,0%	99,5%	ID	98,7%	99,1%	98,9%	98,0%	98,0%	98,7%	98,0%	95,0%	94,9%	95,0%	94,9%	95,0%	93,9%	93,9%	94,6%	94,6%	94,2%	93,3%	93,8%	93,8%	93,3%	93,8%	94,1%	93,7%	93,7%	93,4%	93,5%	95,6%	95,6%	95,6%	96,0%	95,7%	93,3%	93,4%	94,4%	93,1%	94,1%	96,1%	94,4%	93,9%	94,6%	91,2%	80,9%	82,1%	80,7%	82,2%	81,3%	81,5%	81,3%	82,1%	82,8%	80,2%	82,4%	82,1%	81,1%
B. japonicum Is-34	98,3%	97,9%	98,3%	98,3%	98,3%	98,3%	98,6%	98,6%	98,7%	98,3%	98,6%	98,4%	98,7%	98,6%	98,7%	ID	98,4%	98,2%	98,6%	98,6%	99,4%	98,6%	95,0%	95,2%	95,0%	94,9%	95,0%	94,2%	94,2%	95,0%	95,0%	94,4%	93,8%	94,4%	94,2%	93,8%	94,4%	94,9%	94,5%	94,5%	94,2%	94,4%	95,9%	95,9%	95,9%	96,3%	96,0%	93,4%	93,5%	94,6%	93,5%	94,4%	96,4%	94,5%	94,5%	95,0%	91,6%	81,1%	82,4%	81,0%	82,8%	81,5%	81,8%	81,9%	82,4%	83,3%	80,9%	82,9%	82,4%	81,3%
B. japonicum CCBAU25435	97,4%	97,2%	97,4%	97,4%	97,4%	97,4%	97,6%	97,6%	99,1%	97,4%	97,6%	97,8%	99,1%	99,0%	99,1%	98,4%	ID	99,4%	97,6%	97,6%	98,4%	97,6%	94,5%	94,6%	94,5%	94,4%	94,5%	93,7%	93,7%	94,4%	94,4%	93,8%	93,0%	93,5%	93,4%	93,0%	93,5%	93,8%	93,4%	93,4%	93,1%	93,3%	95,3%	95,3%	95,3%	95,7%	95,4%	92,9%	93,0%	94,1%	92,9%	93,8%	95,9%	93,9%	93,7%	94,2%	90,9%	80,9%	81,7%	80,6%	81,9%	81,1%	81,8%	81,1%	81,8%	82,5%	80,0%	82,1%	81,8%	80,6%
B. japonicum CCBAU15618	97,4%	96,9%	97,4%	97,4%	97,4%	97,4%	97,4%	97,4%	98,9%	97,4%	97,4%	97,5%	98,9%	99,3%	98,9%	98,2%	99,4%	ID	97,4%	97,4%	98,2%	97,4%	94,2%	94,4%	94,2%	94,1%	94,2%	93,4%	93,4%	94,1%	94,1%	93,5%	92,7%	93,3%	93,1%	92,7%	93,3%	93,5%	93,1%	93,1%	92,9%	93,0%	95,0%	95,0%	95,0%	95,7%	95,2%	92,6%	92,7%	93,8%	92,6%	93,5%	95,9%	93,7%	93,4%	93,9%	90,7%	80,9%	81,4%	80,3%	81,7%	81,1%	81,5%	81,1%	81,8%	82,2%	80,0%	81,8%	81,5%	80,4%
B. japonicum CCBAU15517	98,3%	97,6%	98,3%	98,3%	98,3%	98,3%	98,7%	98,7%	98,0%	98,3%	100,0%	98,4%	98,0%	97,8%	98,0%	98,6%	97,6%	97,4%	ID	100,0%	98,6%	100,0%	94,8%	94,9%	94,8%	94,6%	94,8%	94,2%	94,2%	94,9%	94,9%	94,6%	93,4%	93,8%	93,9%	93,4%	93,8%	94,4%	93,9%	93,9%	93,7%	93,8%	95,6%	95,6%	95,6%	96,0%	95,7%	93,7%	93,8%	94,6%	93,4%	94,2%	96,1%	94,6%	94,4%	94,5%	91,4%	81,0%	81,8%	80,7%	82,1%	81,1%	81,1%	81,5%	81,9%	82,8%	80,4%	82,2%	82,2%	80,9%
B. japonicum CCBAU15354	98,3%	97,6%	98,3%	98,3%	98,3%	98,3%	98,7%	98,7%	98,0%	98,3%	100,0%	98,4%	98,0%	97,8%	98,0%	98,6%	97,6%	97,4%	100,0%	ID	98,6%	100,0%	94,8%	94,9%	94,8%	94,6%	94,8%	94,2%	94,2%	94,9%	94,9%	94,6%	93,4%	93,8%	93,9%	93,4%	93,8%	94,4%	93,9%	93,9%	93,7%	93,8%	95,6%	95,6%	95,6%	96,0%	95,7%	93,7%	93,8%	94,6%	93,4%	94,2%	96,1%	94,6%	94,4%	94,5%	91,4%	81,0%	81,8%	80,7%	82,1%	81,1%	81,1%	81,5%	81,9%	82,8%	80,4%	82,2%	82,2%	80,9%
B. japonicum USDA38	98,3%	97,9%	98,3%	98,3%	98,3%	98,3%	98,6%	98,6%	98,7%	98,3%	98,6%	98,4%	98,7%	98,6%	98,7%	99,4%	98,4%	98,2%	98,6%	98,6%	ID	98,6%	95,3%	95,4%	95,3%	95,2%	95,3%	94,5%	94,5%	94,8%	94,8%	94,4%	93,8%	94,4%	94,2%	93,8%	94,4%	94,6%	94,2%	94,2%	93,9%	94,1%	95,9%	95,9%	95,9%	96,3%	96,0%	93,4%	93,5%	94,6%	93,5%	94,4%	96,4%	94,5%	94,5%	94,8%	91,4%	81,1%	81,9%	80,7%	82,2%	81,5%	81,8%	81,7%	82,4%	82,8%	80,6%	82,4%	82,1%	81,4%
B. japonicum USDA123	98,3%	97,6%	98,3%	98,3%	98,3%	98,3%	98,7%	98,7%	98,0%	98,3%	100,0%	98,4%	98,0%	97,8%	98,0%	98,6%	97,6%	97,4%	100,0%	100,0%	98,6%	ID	94,8%	94,9%	94,8%	94,6%	94,8%	94,2%	94,2%	94,9%	94,9%	94,6%	93,4%	93,8%	93,9%	93,4%	93,8%	94,4%	93,9%	93,9%	93,7%	93,8%	95,6%	95,6%	95,6%	96,0%	95,7%	93,7%	93,8%	94,6%	93,4%	94,2%	96,1%	94,6%	94,4%	94,5%	91,4%	81,0%	81,8%	80,7%	82,1%	81,1%	81,1%	81,5%	81,9%	82,8%	80,4%	82,2%	82,2%	80,9%
B. diazoefficiens USDA122	94,5%	95,3%	94,5%	94,5%	94,5%	94,5%	94,9%	94,9%	95,0%	94,5%	94,8%	95,2%	95,0%	94,9%	95,0%	95,0%	94,5%	94,2%	94,8%	94,8%	95,3%	94,8%	ID	99,5%	100,0%	99,8%	100,0%	95,6%	95,6%	94,5%	94,5%	94,1%	93,3%	93,8%	93,5%	93,3%	93,4%	93,8%	93,4%	93,4%	93,1%	93,3%	95,6%	95,6%	95,6%	95,9%	95,7%	93,1%	93,3%	94,2%	92,2%	93,4%	95,6%	93,8%	93,5%	96,0%	91,9%	80,3%	82,6%	80,6%	82,6%	80,9%	81,9%	81,0%	81,7%	82,9%	80,4%	82,4%	82,9%	81,0%
B. diazoefficiens NK6	94,6%	95,3%	94,6%	94,6%	94,6%	94,6%	94,9%	94,9%	94,9%	94,6%	94,9%	95,3%	94,9%	94,8%	94,9%	95,2%	94,6%	94,4%	94,9%	94,9%	95,4%	94,9%	99,5%	ID	99,5%	99,7%	99,5%	95,7%	95,7%	94,6%	94,6%	94,4%	93,5%	94,1%	93,8%	93,5%	93,7%	93,8%	93,4%	93,4%	93,1%	93,3%	95,7%	95,7%	95,7%	96,0%	95,9%	93,4%	93,5%	94,5%	92,3%	93,5%	95,7%	93,9%	93,8%	96,0%	92,0%	80,4%	82,8%	80,9%	82,9%	81,1%	82,2%	81,1%	81,8%	83,2%	80,7%	82,6%	83,2%	81,1%
B. diazoefficiens SEMIA5080	94,5%	95,3%	94,5%	94,5%	94,5%	94,5%	94,9%	94,9%	95,0%	94,5%	94,8%	95,2%	95,0%	94,9%	95,0%	95,0%	94,5%	94,2%	94,8%	94,8%	95,3%	94,8%	100,0%	99,5%	ID	99,8%	100,0%	95,6%	95,6%	94,5%	94,5%	94,1%	93,3%	93,8%	93,5%	93,3%	93,4%	93,8%	93,4%	93,4%	93,1%	93,3%	95,6%	95,6%	95,6%	95,9%	95,7%	93,1%	93,3%	94,2%	92,2%	93,4%	95,6%	93,8%	93,5%	96,0%	91,9%	80,3%	82,6%	80,6%	82,6%	80,9%	81,9%	81,0%	81,7%	82,9%	80,4%	82,4%	82,9%	81,0%
B. diazoefficiens USDA110	94,4%	95,2%	94,4%	94,4%	94,4%	94,4%	94,8%	94,8%	94,9%	94,4%	94,6%	95,0%	94,9%	94,8%	94,9%	94,9%	94,4%	94,1%	94,6%	94,6%	95,2%	94,6%	99,8%	99,7%	99,8%	ID	99,8%	95,4%	95,4%	94,4%	94,4%	94,1%	93,3%	93,8%	93,5%	93,3%	93,4%	93,8%	93,4%	93,4%	93,1%	93,3%	95,4%	95,4%	95,4%	95,7%	95,6%	93,1%	93,3%	94,2%	92,0%	93,3%	95,4%	93,7%	93,5%	96,0%	91,8%	80,3%	82,6%	80,6%	82,6%	80,9%	81,9%	81,0%	81,7%	82,9%	80,4%	82,4%	82,9%	80,9%
B. japonicum IS-1	94,5%	95,3%	94,5%	94,5%	94,5%	94,5%	94,9%	94,9%	95,0%	94,5%	94,8%	95,2%	95,0%	94,9%	95,0%	95,0%	94,5%	94,2%	94,8%	94,8%	95,3%	94,8%	100,0%	99,5%	100,0%	99,8%	ID	95,6%	95,6%	94,5%	94,5%	94,1%	93,3%	93,8%	93,5%	93,3%	93,4%	93,8%	93,4%	93,4%	93,1%	93,3%	95,6%	95,6%	95,6%	95,9%	95,7%	93,1%	93,3%	94,2%	92,2%	93,4%	95,6%	93,8%	93,5%	96,0%	91,9%	80,3%	82,6%	80,6%	82,6%	80,9%	81,9%	81,0%	81,7%	82,9%	80,4%	82,4%	82,9%	81,0%
B. japonicum USDA124	93,9%	94,1%	93,9%	93,9%	93,9%	93,9%	94,2%	94,2%	93,9%	93,9%	94,2%	94,6%	93,9%	93,8%	93,9%	94,2%	93,7%	93,4%	94,2%	94,2%	94,5%	94,2%	95,6%	95,7%	95,6%	95,4%	95,6%	ID	100,0%	92,6%	92,6%	93,5%	92,4%	92,6%	92,4%	92,4%	92,6%	93,7%	92,7%	92,7%	92,4%	92,6%	95,0%	94,9%	94,9%	95,4%	95,0%	92,9%	93,0%	93,1%	91,5%	92,6%	94,6%	92,7%	92,7%	94,9%	91,5%	80,3%	82,4%	79,9%	81,9%	81,1%	81,0%	81,1%	81,5%	82,8%	80,3%	82,8%	82,5%	80,2%
B. huanghuaihaiense CCBAU23303	93,9%	94,1%	93,9%	93,9%	93,9%	93,9%	94,2%	94,2%	93,9%	93,9%	94,2%	94,6%	93,9%	93,8%	93,9%	94,2%	93,7%	93,4%	94,2%	94,2%	94,5%	94,2%	95,6%	95,7%	95,6%	95,4%	95,6%	100,0%	ID	92,6%	92,6%	93,5%	92,4%	92,6%	92,4%	92,4%	92,6%	93,7%	92,7%	92,7%	92,4%	92,6%	95,0%	94,9%	94,9%	95,4%	95,0%	92,9%	93,0%	93,1%	91,5%	92,6%	94,6%	92,7%	92,7%	94,9%	91,5%	80,3%	82,4%	79,9%	81,9%	81,1%	81,0%	81,1%	81,5%	82,8%	80,3%	82,8%	82,5%	80,2%
B. japonicum in8p8	94,5%	94,5%	94,5%	94,5%	94,5%	94,5%	94,8%	94,8%	94,6%	94,5%	94,9%	95,4%	94,6%	94,5%	94,6%	95,0%	94,4%	94,1%	94,9%	94,9%	94,8%	94,9%	94,5%	94,6%	94,5%	94,4%	94,5%	92,6%	92,6%	ID	100,0%	94,8%	94,2%	94,8%	94,5%	94,2%	94,4%	93,7%	93,0%	93,0%	92,7%	92,9%	95,0%	95,0%	95,0%	95,3%	95,2%	93,5%	93,7%	94,9%	92,9%	93,8%	94,9%	94,2%	93,8%	93,7%	91,1%	80,3%	81,8%	81,0%	82,6%	81,1%	81,3%	80,9%	81,0%	82,2%	81,0%	81,3%	81,9%	80,3%
B. japonicum is5	94,5%	94,5%	94,5%	94,5%	94,5%	94,5%	94,8%	94,8%	94,6%	94,5%	94,9%	95,4%	94,6%	94,5%	94,6%	95,0%	94,4%	94,1%	94,9%	94,9%	94,8%	94,9%	94,5%	94,6%	94,5%	94,4%	94,5%	92,6%	92,6%	100,0%	ID	94,8%	94,2%	94,8%	94,5%	94,2%	94,4%	93,7%	93,0%	93,0%	92,7%	92,9%	95,0%	95,0%	95,0%	95,3%	95,2%	93,5%	93,7%	94,9%	92,9%	93,8%	94,9%	94,2%	93,8%	93,7%	91,1%	80,3%	81,8%	81,0%	82,6%	81,1%	81,3%	80,9%	81,0%	82,2%	81,0%	81,3%	81,9%	80,3%
B. japonicum 22	93,9%	94,5%	93,9%	93,9%	93,9%	93,9%	94,4%	94,4%	94,2%	93,9%	94,6%	94,5%	94,2%	93,9%	94,2%	94,4%	93,8%	93,5%	94,6%	94,6%	94,4%	94,6%	94,1%	94,4%	94,1%	94,1%	94,1%	93,5%	93,5%	94,8%	94,8%	ID	94,5%	94,5%	94,9%	94,5%	94,6%	93,7%	93,7%	93,7%	93,4%	93,5%	95,3%	95,3%	95,3%	95,6%	95,4%	95,7%	95,9%	96,1%	92,6%	93,0%	94,6%	93,8%	93,1%	94,5%	91,1%	80,2%	81,0%	81,3%	81,4%	80,9%	81,3%	81,0%	81,3%	81,0%	80,4%	80,9%	81,3%	79,3%
B. yuanmingense CCBAU10071	93,1%	93,5%	93,1%	93,1%	93,1%	93,1%	93,4%	93,4%	93,3%	93,1%	93,4%	93,5%	93,3%	93,1%	93,3%	93,8%	93,0%	92,7%	93,4%	93,4%	93,8%	93,4%	93,3%	93,5%	93,3%	93,3%	93,3%	92,4%	92,4%	94,2%	94,2%	94,5%	ID	98,3%	98,4%	100,0%	98,6%	92,9%	93,3%	93,3%	93,3%	93,4%	94,6%	94,6%	94,6%	94,8%	94,8%	93,8%	93,9%	95,3%	91,6%	91,6%	94,1%	92,9%	93,0%	93,4%	91,1%	78,7%	80,7%	80,2%	81,0%	79,8%	81,1%	79,1%	79,5%	80,6%	79,5%	81,0%	81,1%	78,8%
B. yuanmingense BR3267	93,5%	93,7%	93,5%	93,5%	93,5%	93,5%	93,8%	93,8%	93,8%	93,5%	93,8%	93,9%	93,8%	93,7%	93,8%	94,4%	93,5%	93,3%	93,8%	93,8%	94,4%	93,8%	93,8%	94,1%	93,8%	93,8%	93,8%	92,6%	92,6%	94,8%	94,8%	94,5%	98,3%	ID	99,5%	98,3%	99,4%	93,1%	93,4%	93,4%	93,1%	93,3%	95,0%	95,0%	95,0%	95,2%	95,2%	94,4%	94,5%	95,4%	92,4%	92,9%	94,8%	93,8%	94,2%	94,2%	90,8%	79,5%	81,9%	80,2%	82,2%	80,3%	81,8%	79,6%	80,0%	81,5%	80,2%	81,7%	81,7%	79,3%
B. yuanmingense CCBAU05623	93,4%	93,5%	93,4%	93,4%	93,4%	93,4%	93,8%	93,8%	93,8%	93,4%	93,9%	93,8%	93,8%	93,5%	93,8%	94,2%	93,4%	93,1%	93,9%	93,9%	94,2%	93,9%	93,5%	93,8%	93,5%	93,5%	93,5%	92,4%	92,4%	94,5%	94,5%	94,9%	98,4%	99,5%	ID	98,4%	99,3%	93,0%	93,3%	93,3%	93,0%	93,1%	94,9%	94,9%	94,9%	95,0%	95,0%	94,4%	94,5%	95,6%	92,4%	92,6%	94,6%	94,1%	93,9%	94,4%	91,2%	79,5%	81,7%	80,4%	81,9%	80,3%	81,5%	79,6%	80,0%	81,4%	80,2%	81,4%	81,4%	79,2%
B. yuanmingense CCBAU25021	93,1%	93,5%	93,1%	93,1%	93,1%	93,1%	93,4%	93,4%	93,3%	93,1%	93,4%	93,5%	93,3%	93,1%	93,3%	93,8%	93,0%	92,7%	93,4%	93,4%	93,8%	93,4%	93,3%	93,5%	93,3%	93,3%	93,3%	92,4%	92,4%	94,2%	94,2%	94,5%	100,0%	98,3%	98,4%	ID	98,6%	92,9%	93,3%	93,3%	93,3%	93,4%	94,6%	94,6%	94,6%	94,8%	94,8%	93,8%	93,9%	95,3%	91,6%	91,6%	94,1%	92,9%	93,0%	93,4%	91,1%	78,7%	80,7%	80,2%	81,0%	79,8%	81,1%	79,1%	79,5%	80,6%	79,5%	81,0%	81,1%	78,8%
B. yuanmingense CCBAU35157	93,5%	93,8%	93,5%	93,5%	93,5%	93,5%	93,8%	93,8%	93,8%	93,5%	93,8%	93,9%	93,8%	93,7%	93,8%	94,4%	93,5%	93,3%	93,8%	93,8%	94,4%	93,8%	93,4%	93,7%	93,4%	93,4%	93,4%	92,6%	92,6%	94,4%	94,4%	94,6%	98,6%	99,4%	99,3%	98,6%	ID	93,3%	93,5%	93,5%	93,3%	93,4%	95,0%	95,0%	95,0%	95,2%	95,2%	94,5%	94,6%	95,4%	92,0%	92,4%	94,6%	93,4%	93,8%	94,1%	90,8%	79,3%	81,5%	80,0%	81,8%	79,9%	81,7%	79,5%	79,9%	81,1%	79,8%	81,5%	81,3%	78,9%
B. canariense BTA-1	94,6%	94,6%	94,6%	94,6%	94,6%	94,6%	94,8%	94,8%	94,1%	94,6%	94,4%	94,8%	94,1%	93,9%	94,1%	94,9%	93,8%	93,5%	94,4%	94,4%	94,6%	94,4%	93,8%	93,8%	93,8%	93,8%	93,8%	93,7%	93,7%	93,7%	93,7%	93,7%	92,9%	93,1%	93,0%	92,9%	93,3%	ID	97,2%	97,2%	96,9%	97,1%	94,5%	94,5%	94,5%	95,0%	94,6%	92,7%	92,9%	93,3%	92,2%	92,9%	95,0%	93,3%	93,4%	93,5%	90,4%	79,5%	81,3%	79,2%	81,7%	80,0%	79,8%	80,3%	80,4%	81,9%	79,3%	82,1%	81,5%	78,5%
B. canariense UBMAN05	93,9%	94,2%	93,9%	93,9%	93,9%	93,9%	94,4%	94,4%	93,7%	93,9%	93,9%	94,4%	93,7%	93,5%	93,7%	94,5%	93,4%	93,1%	93,9%	93,9%	94,2%	93,9%	93,4%	93,4%	93,4%	93,4%	93,4%	92,7%	92,7%	93,0%	93,0%	93,7%	93,3%	93,4%	93,3%	93,3%	93,5%	97,2%	ID	100,0%	99,7%	99,8%	94,6%	94,6%	94,6%	94,8%	94,8%	92,9%	93,0%	93,4%	92,3%	92,2%	95,0%	92,9%	93,4%	93,5%	89,9%	79,9%	81,3%	80,4%	81,7%	80,6%	80,7%	81,0%	81,3%	82,2%	80,2%	81,9%	81,5%	79,2%
B. canariense UBMA510	93,9%	94,2%	93,9%	93,9%	93,9%	93,9%	94,4%	94,4%	93,7%	93,9%	93,9%	94,4%	93,7%	93,5%	93,7%	94,5%	93,4%	93,1%	93,9%	93,9%	94,2%	93,9%	93,4%	93,4%	93,4%	93,4%	93,4%	92,7%	92,7%	93,0%	93,0%	93,7%	93,3%	93,4%	93,3%	93,3%	93,5%	97,2%	100,0%	ID	99,7%	99,8%	94,6%	94,6%	94,6%	94,8%	94,8%	92,9%	93,0%	93,4%	92,3%	92,2%	95,0%	92,9%	93,4%	93,5%	89,9%	79,9%	81,3%	80,4%	81,7%	80,6%	80,7%	81,0%	81,3%	82,2%	80,2%	81,9%	81,5%	79,2%
B. canariense UBMA183	93,7%	93,9%	93,7%	93,7%	93,7%	93,7%	94,1%	94,1%	93,4%	93,7%	93,7%	94,1%	93,4%	93,3%	93,4%	94,2%	93,1%	92,9%	93,7%	93,7%	93,9%	93,7%	93,1%	93,1%	93,1%	93,1%	93,1%	92,4%	92,4%	92,7%	92,7%	93,4%	93,3%	93,1%	93,0%	93,3%	93,3%	96,9%	99,7%	99,7%	ID	99,8%	94,4%	94,4%	94,4%	94,5%	94,5%	92,6%	92,7%	93,1%	92,0%	91,9%	94,8%	92,7%	93,1%	93,3%	89,9%	79,6%	81,0%	80,2%	81,4%	80,3%	80,4%	80,7%	81,0%	81,9%	79,9%	81,7%	81,3%	78,9%
B. canariense UBMA195	93,8%	94,1%	93,8%	93,8%	93,8%	93,8%	94,2%	94,2%	93,5%	93,8%	93,8%	94,2%	93,5%	93,4%	93,5%	94,4%	93,3%	93,0%	93,8%	93,8%	94,1%	93,8%	93,3%	93,3%	93,3%	93,3%	93,3%	92,6%	92,6%	92,9%	92,9%	93,5%	93,4%	93,3%	93,1%	93,4%	93,4%	97,1%	99,8%	99,8%	99,8%	ID	94,5%	94,5%	94,5%	94,6%	94,6%	92,7%	92,9%	93,3%	92,2%	92,0%	94,9%	92,7%	93,3%	93,4%	90,0%	79,8%	81,1%	80,3%	81,5%	80,4%	80,6%	80,9%	81,1%	82,1%	80,0%	81,8%	81,4%	79,1%
B. liaoningense LMG18230	95,3%	95,7%	95,3%	95,3%	95,3%	95,3%	95,6%	95,6%	95,6%	95,3%	95,6%	96,0%	95,6%	95,4%	95,6%	95,9%	95,3%	95,0%	95,6%	95,6%	95,9%	95,6%	95,6%	95,7%	95,6%	95,4%	95,6%	95,0%	95,0%	95,0%	95,0%	95,3%	94,6%	95,0%	94,9%	94,6%	95,0%	94,5%	94,6%	94,6%	94,4%	94,5%	ID	99,7%	99,7%	98,3%	99,8%	94,2%	94,4%	95,0%	93,5%	94,2%	96,5%	94,6%	94,5%	95,0%	91,4%	80,3%	82,1%	79,9%	82,1%	80,7%	81,4%	80,6%	81,0%	82,4%	80,0%	82,1%	81,8%	79,9%
B. liaoningense CCBAU05525	95,3%	95,7%	95,3%	95,3%	95,3%	95,3%	95,6%	95,6%	95,6%	95,3%	95,6%	96,0%	95,6%	95,4%	95,6%	95,9%	95,3%	95,0%	95,6%	95,6%	95,9%	95,6%	95,6%	95,7%	95,6%	95,4%	95,6%	94,9%	94,9%	95,0%	95,0%	95,3%	94,6%	95,0%	94,9%	94,6%	95,0%	94,5%	94,6%	94,6%	94,4%	94,5%	99,7%	ID	100,0%	98,3%	99,8%	94,2%	94,4%	95,0%	93,5%	94,2%	96,5%	94,6%	94,5%	95,0%	91,2%	80,3%	82,2%	79,9%	82,2%	80,7%	81,4%	80,6%	81,0%	82,5%	80,0%	82,2%	81,9%	79,9%
B. liaoningense CCBAU83689	95,3%	95,7%	95,3%	95,3%	95,3%	95,3%	95,6%	95,6%	95,6%	95,3%	95,6%	96,0%	95,6%	95,4%	95,6%	95,9%	95,3%	95,0%	95,6%	95,6%	95,9%	95,6%	95,6%	95,7%	95,6%	95,4%	95,6%	94,9%	94,9%	95,0%	95,0%	95,3%	94,6%	95,0%	94,9%	94,6%	95,0%	94,5%	94,6%	94,6%	94,4%	94,5%	99,7%	100,0%	ID	98,3%	99,8%	94,2%	94,4%	95,0%	93,5%	94,2%	96,5%	94,6%	94,5%	95,0%	91,2%	80,3%	82,2%	79,9%	82,2%	80,7%	81,4%	80,6%	81,0%	82,5%	80,0%	82,2%	81,9%	79,9%
B. liaoningense CCNWSX0360	95,7%	96,1%	95,7%	95,7%	95,7%	95,7%	96,0%	96,0%	96,0%	95,7%	96,0%	96,4%	96,0%	96,1%	96,0%	96,3%	95,7%	95,7%	96,0%	96,0%	96,3%	96,0%	95,9%	96,0%	95,9%	95,7%	95,9%	95,4%	95,4%	95,3%	95,3%	95,6%	94,8%	95,2%	95,0%	94,8%	95,2%	95,0%	94,8%	94,8%	94,5%	94,6%	98,3%	98,3%	98,3%	ID	98,4%	94,8%	94,9%	95,4%	93,0%	93,9%	96,9%	94,6%	94,6%	95,3%	91,6%	80,9%	82,1%	80,0%	82,1%	81,3%	81,3%	81,3%	81,7%	82,4%	80,6%	82,4%	81,8%	80,0%
B. japonicum USDA135	95,4%	95,9%	95,4%	95,4%	95,4%	95,4%	95,7%	95,7%	95,7%	95,4%	95,7%	96,1%	95,7%	95,6%	95,7%	96,0%	95,4%	95,2%	95,7%	95,7%	96,0%	95,7%	95,7%	95,9%	95,7%	95,6%	95,7%	95,0%	95,0%	95,2%	95,2%	95,4%	94,8%	95,2%	95,0%	94,8%	95,2%	94,6%	94,8%	94,8%	94,5%	94,6%	99,8%	99,8%	99,8%	98,4%	ID	94,4%	94,5%	95,2%	93,7%	94,4%	96,7%	94,8%	94,6%	95,2%	91,4%	80,4%	82,2%	80,0%	82,2%	80,9%	81,5%	80,7%	81,1%	82,5%	80,2%	82,2%	81,9%	80,0%
B. japonicum USDA4	92,9%	93,5%	92,9%	92,9%	92,9%	92,9%	93,3%	93,3%	93,3%	92,9%	93,7%	93,7%	93,3%	93,0%	93,3%	93,4%	92,9%	92,6%	93,7%	93,7%	93,4%	93,7%	93,1%	93,4%	93,1%	93,1%	93,1%	92,9%	92,9%	93,5%	93,5%	95,7%	93,8%	94,4%	94,4%	93,8%	94,5%	92,7%	92,9%	92,9%	92,6%	92,7%	94,2%	94,2%	94,2%	94,8%	94,4%	ID	99,8%	94,6%	90,9%	91,6%	94,1%	92,4%	91,9%	93,7%	89,4%	78,9%	79,9%	79,9%	80,2%	79,2%	81,0%	79,3%	79,8%	80,6%	78,9%	79,9%	80,6%	78,9%
B. ottawaense OO99	93,0%	93,7%	93,0%	93,0%	93,0%	93,0%	93,4%	93,4%	93,4%	93,0%	93,8%	93,8%	93,4%	93,1%	93,4%	93,5%	93,0%	92,7%	93,8%	93,8%	93,5%	93,8%	93,3%	93,5%	93,3%	93,3%	93,3%	93,0%	93,0%	93,7%	93,7%	95,9%	93,9%	94,5%	94,5%	93,9%	94,6%	92,9%	93,0%	93,0%	92,7%	92,9%	94,4%	94,4%	94,4%	94,9%	94,5%	99,8%	ID	94,8%	91,1%	91,8%	94,2%	92,6%	92,0%	93,8%	89,6%	78,9%	79,9%	79,9%	80,2%	79,2%	81,0%	79,3%	79,8%	80,6%	78,9%	79,9%	80,6%	79,1%
B. daqingense CCBAU15774	94,2%	94,5%	94,2%	94,2%	94,2%	94,2%	94,5%	94,5%	94,4%	94,2%	94,6%	94,9%	94,4%	94,2%	94,4%	94,6%	94,1%	93,8%	94,6%	94,6%	94,6%	94,6%	94,2%	94,5%	94,2%	94,2%	94,2%	93,1%	93,1%	94,9%	94,9%	96,1%	95,3%	95,4%	95,6%	95,3%	95,4%	93,3%	93,4%	93,4%	93,1%	93,3%	95,0%	95,0%	95,0%	95,4%	95,2%	94,6%	94,8%	ID	91,8%	92,3%	95,4%	93,1%	93,1%	94,2%	90,9%	79,8%	82,1%	80,3%	82,2%	80,0%	81,4%	79,9%	80,9%	81,7%	79,5%	81,7%	81,8%	79,6%
B. cytisi CTAW11	93,1%	93,1%	93,1%	93,1%	93,1%	93,1%	93,4%	93,4%	93,1%	93,1%	93,4%	93,4%	93,1%	93,0%	93,1%	93,5%	92,9%	92,6%	93,4%	93,4%	93,5%	93,4%	92,2%	92,3%	92,2%	92,0%	92,2%	91,5%	91,5%	92,9%	92,9%	92,6%	91,6%	92,4%	92,4%	91,6%	92,0%	92,2%	92,3%	92,3%	92,0%	92,2%	93,5%	93,5%	93,5%	93,0%	93,7%	90,9%	91,1%	91,8%	ID	96,5%	94,1%	96,0%	95,6%	91,9%	90,8%	80,6%	82,6%	80,7%	82,8%	81,5%	81,8%	81,0%	81,3%	82,4%	80,9%	82,1%	82,6%	79,9%
B. rifense CTAW71	93,9%	94,1%	93,9%	93,9%	93,9%	93,9%	94,5%	94,5%	94,1%	93,9%	94,2%	94,4%	94,1%	93,9%	94,1%	94,4%	93,8%	93,5%	94,2%	94,2%	94,4%	94,2%	93,4%	93,5%	93,4%	93,3%	93,4%	92,6%	92,6%	93,8%	93,8%	93,0%	91,6%	92,9%	92,6%	91,6%	92,4%	92,9%	92,2%	92,2%	91,9%	92,0%	94,2%	94,2%	94,2%	93,9%	94,4%	91,6%	91,8%	92,3%	96,5%	ID	94,2%	96,9%	96,4%	93,1%	90,9%	80,6%	82,8%	80,3%	82,9%	81,0%	82,4%	81,0%	81,1%	83,0%	80,2%	82,9%	83,0%	80,0%
B. arachidis LMG26795	95,9%	96,3%	95,9%	95,9%	95,9%	95,9%	96,1%	96,1%	96,1%	95,9%	96,1%	96,3%	96,1%	96,3%	96,1%	96,4%	95,9%	95,9%	96,1%	96,1%	96,4%	96,1%	95,6%	95,7%	95,6%	95,4%	95,6%	94,6%	94,6%	94,9%	94,9%	94,6%	94,1%	94,8%	94,5%	94,1%	94,6%	95,0%	95,0%	95,0%	94,8%	94,9%	96,5%	96,5%	96,5%	96,9%	96,7%	94,1%	94,2%	95,4%	94,1%	94,2%	ID	95,2%	94,5%	94,8%	91,2%	81,7%	82,6%	80,4%	82,9%	81,1%	81,8%	81,7%	82,4%	82,5%	80,2%	82,5%	82,1%	80,6%
B. ganzhouense RITF806	94,1%	94,2%	94,1%	94,1%	94,1%	94,1%	94,8%	94,8%	94,4%	94,1%	94,6%	94,5%	94,4%	94,1%	94,4%	94,5%	93,9%	93,7%	94,6%	94,6%	94,5%	94,6%	93,8%	93,9%	93,8%	93,7%	93,8%	92,7%	92,7%	94,2%	94,2%	93,8%	92,9%	93,8%	94,1%	92,9%	93,4%	93,3%	92,9%	92,9%	92,6%	92,7%	94,6%	94,6%	94,6%	94,6%	94,8%	92,4%	92,6%	93,1%	96,0%	96,9%	95,2%	ID	96,4%	93,3%	92,2%	80,6%	82,4%	80,2%	82,9%	81,4%	81,5%	81,3%	81,0%	83,0%	80,2%	83,3%	82,9%	79,9%
B. manausense BR3351	94,1%	94,2%	94,1%	94,1%	94,1%	94,1%	94,4%	94,4%	93,9%	94,1%	94,4%	94,5%	93,9%	93,8%	93,9%	94,5%	93,7%	93,4%	94,4%	94,4%	94,5%	94,4%	93,5%	93,8%	93,5%	93,5%	93,5%	92,7%	92,7%	93,8%	93,8%	93,1%	93,0%	94,2%	93,9%	93,0%	93,8%	93,4%	93,4%	93,4%	93,1%	93,3%	94,5%	94,5%	94,5%	94,5%	94,6%	91,9%	92,0%	93,1%	95,6%	96,4%	94,5%	96,4%	ID	93,4%	92,0%	81,7%	82,4%	80,4%	82,8%	81,8%	82,6%	82,4%	82,2%	83,2%	81,1%	83,3%	82,6%	80,2%
B. stylosanthis BR446	93,9%	94,8%	93,9%	93,9%	93,9%	93,9%	94,5%	94,5%	94,6%	93,9%	94,5%	95,2%	94,6%	94,4%	94,6%	95,0%	94,2%	93,9%	94,5%	94,5%	94,8%	94,5%	96,0%	96,0%	96,0%	96,0%	96,0%	94,9%	94,9%	93,7%	93,7%	94,5%	93,4%	94,2%	94,4%	93,4%	94,1%	93,5%	93,5%	93,5%	93,3%	93,4%	95,0%	95,0%	95,0%	95,3%	95,2%	93,7%	93,8%	94,2%	91,9%	93,1%	94,8%	93,3%	93,4%	ID	92,3%	81,4%	83,6%	81,3%	83,4%	81,4%	82,6%	81,8%	82,4%	84,5%	81,7%	83,7%	83,3%	81,3%
B. neotropicale BR10247	91,2%	91,2%	91,2%	91,2%	91,2%	91,2%	91,4%	91,4%	91,2%	91,2%	91,4%	91,9%	91,2%	90,9%	91,2%	91,6%	90,9%	90,7%	91,4%	91,4%	91,4%	91,4%	91,9%	92,0%	91,9%	91,8%	91,9%	91,5%	91,5%	91,1%	91,1%	91,1%	91,1%	90,8%	91,2%	91,1%	90,8%	90,4%	89,9%	89,9%	89,9%	90,0%	91,4%	91,2%	91,2%	91,6%	91,4%	89,4%	89,6%	90,9%	90,8%	90,9%	91,2%	92,2%	92,0%	92,3%	ID	80,6%	81,9%	80,6%	82,6%	80,3%	81,5%	81,7%	80,9%	81,9%	80,4%	81,7%	82,1%	79,1%
B. lablabi CCBAU23086	80,7%	81,1%	80,7%	80,7%	80,7%	80,7%	80,9%	80,9%	80,9%	80,7%	81,0%	81,0%	80,9%	80,9%	80,9%	81,1%	80,9%	80,9%	81,0%	81,0%	81,1%	81,0%	80,3%	80,4%	80,3%	80,3%	80,3%	80,3%	80,3%	80,3%	80,3%	80,2%	78,7%	79,5%	79,5%	78,7%	79,3%	79,5%	79,9%	79,9%	79,6%	79,8%	80,3%	80,3%	80,3%	80,9%	80,4%	78,9%	78,9%	79,8%	80,6%	80,6%	81,7%	80,6%	81,7%	81,4%	80,6%	ID	85,2%	90,1%	85,8%	92,4%	85,8%	96,5%	96,1%	85,5%	91,4%	85,5%	84,8%	81,1%
B. pachyrhizi PAC48	81,8%	81,8%	81,8%	81,8%	81,8%	81,8%	81,9%	81,9%	82,1%	81,8%	81,8%	82,6%	82,1%	81,9%	82,1%	82,4%	81,7%	81,4%	81,8%	81,8%	81,9%	81,8%	82,6%	82,8%	82,6%	82,6%	82,6%	82,4%	82,4%	81,8%	81,8%	81,0%	80,7%	81,9%	81,7%	80,7%	81,5%	81,3%	81,3%	81,3%	81,0%	81,1%	82,1%	82,2%	82,2%	82,1%	82,2%	79,9%	79,9%	82,1%	82,6%	82,8%	82,6%	82,4%	82,4%	83,6%	81,9%	85,2%	ID	85,8%	97,4%	85,8%	87,9%	85,9%	85,9%	95,0%	86,2%	95,0%	95,3%	82,5%
B. valentinum LmjM3	80,3%	80,7%	80,3%	80,3%	80,3%	80,3%	80,4%	80,4%	80,7%	80,3%	80,7%	80,7%	80,7%	80,4%	80,7%	81,0%	80,6%	80,3%	80,7%	80,7%	80,7%	80,7%	80,6%	80,9%	80,6%	80,6%	80,6%	79,9%	79,9%	81,0%	81,0%	81,3%	80,2%	80,2%	80,4%	80,2%	80,0%	79,2%	80,4%	80,4%	80,2%	80,3%	79,9%	79,9%	79,9%	80,0%	80,0%	79,9%	79,9%	80,3%	80,7%	80,3%	80,4%	80,2%	80,4%	81,3%	80,6%	90,1%	85,8%	ID	85,8%	92,0%	87,3%	90,7%	91,5%	85,5%	91,1%	85,6%	85,9%	81,9%
B. elkanii USDA76	82,4%	82,2%	82,4%	82,4%	82,4%	82,4%	82,2%	82,2%	82,2%	82,4%	82,1%	82,6%	82,2%	82,1%	82,2%	82,8%	81,9%	81,7%	82,1%	82,1%	82,2%	82,1%	82,6%	82,9%	82,6%	82,6%	82,6%	81,9%	81,9%	82,6%	82,6%	81,4%	81,0%	82,2%	81,9%	81,0%	81,8%	81,7%	81,7%	81,7%	81,4%	81,5%	82,1%	82,2%	82,2%	82,1%	82,2%	80,2%	80,2%	82,2%	82,8%	82,9%	82,9%	82,9%	82,8%	83,4%	82,6%	85,8%	97,4%	85,8%	ID	86,2%	88,2%	86,4%	85,9%	95,3%	86,6%	95,2%	95,0%	82,4%
B. retamae Ro19	80,9%	80,7%	80,9%	80,9%	80,9%	80,9%	81,0%	81,0%	81,3%	80,9%	81,1%	81,4%	81,3%	81,3%	81,3%	81,5%	81,1%	81,1%	81,1%	81,1%	81,5%	81,1%	80,9%	81,1%	80,9%	80,9%	80,9%	81,1%	81,1%	81,1%	81,1%	80,9%	79,8%	80,3%	80,3%	79,8%	79,9%	80,0%	80,6%	80,6%	80,3%	80,4%	80,7%	80,7%	80,7%	81,3%	80,9%	79,2%	79,2%	80,0%	81,5%	81,0%	81,1%	81,4%	81,8%	81,4%	80,3%	92,4%	85,8%	92,0%	86,2%	ID	87,0%	92,4%	93,3%	85,9%	94,2%	86,6%	86,0%	82,1%
B. erythrophlei GAS478	81,3%	81,3%	81,3%	81,3%	81,3%	81,3%	81,1%	81,1%	81,5%	81,3%	81,1%	81,5%	81,5%	81,4%	81,5%	81,8%	81,8%	81,5%	81,1%	81,1%	81,8%	81,1%	81,9%	82,2%	81,9%	81,9%	81,9%	81,0%	81,0%	81,3%	81,3%	81,3%	81,1%	81,8%	81,5%	81,1%	81,7%	79,8%	80,7%	80,7%	80,4%	80,6%	81,4%	81,4%	81,4%	81,3%	81,5%	81,0%	81,0%	81,4%	81,8%	82,4%	81,8%	81,5%	82,6%	82,6%	81,5%	85,8%	87,9%	87,3%	88,2%	87,0%	ID	87,1%	87,7%	88,1%	87,3%	87,9%	87,5%	83,0%
B. paxllaeri LMTR21	81,4%	81,5%	81,4%	81,4%	81,4%	81,4%	81,5%	81,5%	81,3%	81,4%	81,5%	81,3%	81,3%	81,3%	81,3%	81,9%	81,1%	81,1%	81,5%	81,5%	81,7%	81,5%	81,0%	81,1%	81,0%	81,0%	81,0%	81,1%	81,1%	80,9%	80,9%	81,0%	79,1%	79,6%	79,6%	79,1%	79,5%	80,3%	81,0%	81,0%	80,7%	80,9%	80,6%	80,6%	80,6%	81,3%	80,7%	79,3%	79,3%	79,9%	81,0%	81,0%	81,7%	81,3%	82,4%	81,8%	81,7%	96,5%	85,9%	90,7%	86,4%	92,4%	87,1%	ID	96,4%	85,9%	92,2%	86,2%	85,8%	81,3%
B. jicamae PAC68	81,4%	81,9%	81,4%	81,4%	81,4%	81,4%	81,8%	81,8%	82,1%	81,4%	81,9%	81,8%	82,1%	82,1%	82,1%	82,4%	81,8%	81,8%	81,9%	81,9%	82,4%	81,9%	81,7%	81,8%	81,7%	81,7%	81,7%	81,5%	81,5%	81,0%	81,0%	81,3%	79,5%	80,0%	80,0%	79,5%	79,9%	80,4%	81,3%	81,3%	81,0%	81,1%	81,0%	81,0%	81,0%	81,7%	81,1%	79,8%	79,8%	80,9%	81,3%	81,1%	82,4%	81,0%	82,2%	82,4%	80,9%	96,1%	85,9%	91,5%	85,9%	93,3%	87,7%	96,4%	ID	85,8%	92,4%	86,3%	85,6%	81,7%
B. embrapense SEMIA6208	82,6%	82,4%	82,6%	82,6%	82,6%	82,6%	83,0%	83,0%	82,8%	82,6%	82,8%	83,4%	82,8%	82,4%	82,8%	83,3%	82,5%	82,2%	82,8%	82,8%	82,8%	82,8%	82,9%	83,2%	82,9%	82,9%	82,9%	82,8%	82,8%	82,2%	82,2%	81,0%	80,6%	81,5%	81,4%	80,6%	81,1%	81,9%	82,2%	82,2%	81,9%	82,1%	82,4%	82,5%	82,5%	82,4%	82,5%	80,6%	80,6%	81,7%	82,4%	83,0%	82,5%	83,0%	83,2%	84,5%	81,9%	85,5%	95,0%	85,5%	95,3%	85,9%	88,1%	85,9%	85,8%	ID	86,4%	95,0%	94,8%	83,4%
B. icense LMTR13	80,3%	79,9%	80,3%	80,3%	80,3%	80,3%	80,4%	80,4%	80,2%	80,3%	80,4%	80,4%	80,2%	80,2%	80,2%	80,9%	80,0%	80,0%	80,4%	80,4%	80,6%	80,4%	80,4%	80,7%	80,4%	80,4%	80,4%	80,3%	80,3%	81,0%	81,0%	80,4%	79,5%	80,2%	80,2%	79,5%	79,8%	79,3%	80,2%	80,2%	79,9%	80,0%	80,0%	80,0%	80,0%	80,6%	80,2%	78,9%	78,9%	79,5%	80,9%	80,2%	80,2%	80,2%	81,1%	81,7%	80,4%	91,4%	86,2%	91,1%	86,6%	94,2%	87,3%	92,2%	92,4%	86,4%	ID	86,3%	86,2%	81,8%
B. mercantei SEMIA6399	82,5%	82,2%	82,5%	82,5%	82,5%	82,5%	82,6%	82,6%	82,4%	82,5%	82,2%	83,0%	82,4%	82,2%	82,4%	82,9%	82,1%	81,8%	82,2%	82,2%	82,4%	82,2%	82,4%	82,6%	82,4%	82,4%	82,4%	82,8%	82,8%	81,3%	81,3%	80,9%	81,0%	81,7%	81,4%	81,0%	81,5%	82,1%	81,9%	81,9%	81,7%	81,8%	82,1%	82,2%	82,2%	82,4%	82,2%	79,9%	79,9%	81,7%	82,1%	82,9%	82,5%	83,3%	83,3%	83,7%	81,7%	85,5%	95,0%	85,6%	95,2%	86,6%	87,9%	86,2%	86,3%	95,0%	86,3%	ID	95,9%	82,2%
B. viridifuturi SEMIA690	82,5%	82,2%	82,5%	82,5%	82,5%	82,5%	82,1%	82,1%	82,1%	82,5%	82,2%	82,5%	82,1%	81,9%	82,1%	82,4%	81,8%	81,5%	82,2%	82,2%	82,1%	82,2%	82,9%	83,2%	82,9%	82,9%	82,9%	82,5%	82,5%	81,9%	81,9%	81,3%	81,1%	81,7%	81,4%	81,1%	81,3%	81,5%	81,5%	81,5%	81,3%	81,4%	81,8%	81,9%	81,9%	81,8%	81,9%	80,6%	80,6%	81,8%	82,6%	83,0%	82,1%	82,9%	82,6%	83,3%	82,1%	84,8%	95,3%	85,9%	95,0%	86,0%	87,5%	85,8%	85,6%	94,8%	86,2%	95,9%	ID	82,2%
Rhodopseudomonas palustris CGA009	80,4%	80,2%	80,4%	80,4%	80,4%	80,4%	81,0%	81,0%	81,1%	80,4%	80,9%	81,3%	81,1%	80,7%	81,1%	81,3%	80,6%	80,4%	80,9%	80,9%	81,4%	80,9%	81,0%	81,1%	81,0%	80,9%	81,0%	80,2%	80,2%	80,3%	80,3%	79,3%	78,8%	79,3%	79,2%	78,8%	78,9%	78,5%	79,2%	79,2%	78,9%	79,1%	79,9%	79,9%	79,9%	80,0%	80,0%	78,9%	79,1%	79,6%	79,9%	80,0%	80,6%	79,9%	80,2%	81,3%	79,1%	81,1%	82,5%	81,9%	82,4%	82,1%	83,0%	81,3%	81,7%	83,4%	81,8%	82,2%	82,2%	ID

Supplementary Table S3. *glnII* gene sequence similarity between strains used in the study

	Bradyrhizobium sp. JAN6	*Bradyrhizobium* sp. JAN10	*Bradyrhizobium* sp. GER1	*Bradyrhizobium* sp. GER32	*Bradyrhizobium* sp. ZAR4	*Bradyrhizobium* sp. ZAR12	*Bradyrhizobium* sp. SZCZ11	*Bradyrhizobium* sp. SZCZ20	*B. japonicum* USDA6	*B. japonicum* E109	*B. japonicum* J5	*B. japonicum* SEMIA5079	*B. japonicum* USDA38	*B. japonicum* CCBAU15517	*B. japonicum* CCBAU15618	*B. japonicum* CCBAU25435	*B. japonicum* CCBAU83623	*B. japonicum* FN1	*B. japonicum* UBMA197	*B. japonicum* Is-34	*B. japonicum* USDA123	*B. japonicum* CCBAU15354	*B. diazoefficiens* NK6
Bradyrhizobium sp. JAN6	ID	99,5%	98,9%	100,0%	100,0%	100,0%	99,3%	99,3%	98,5%	98,5%	99,5%	98,5%	98,1%	98,5%	97,7%	98,3%	98,5%	98,5%	100,0%	98,1%	98,3%	98,5%	97,5%
Bradyrhizobium sp. JAN10	99,5%	ID	98,5%	99,5%	99,5%	99,5%	98,9%	98,9%	98,5%	98,5%	99,1%	98,5%	98,1%	98,1%	97,7%	98,3%	98,1%	98,5%	99,5%	97,7%	97,9%	98,1%	97,5%
Bradyrhizobium sp. GER1	98,9%	98,5%	ID	98,9%	98,9%	98,9%	99,3%	99,3%	98,7%	98,7%	98,7%	98,7%	98,3%	98,5%	97,5%	98,5%	98,5%	98,7%	98,9%	97,9%	98,3%	98,5%	97,5%
Bradyrhizobium sp. GER32	100,0%	99,5%	98,9%	ID	100,0%	100,0%	99,3%	99,3%	98,5%	98,5%	99,5%	98,5%	98,1%	98,5%	97,7%	98,3%	98,5%	98,5%	100,0%	98,1%	98,3%	98,5%	97,5%
Bradyrhizobium sp. ZAR4	100,0%	99,5%	98,9%	100,0%	ID	100,0%	99,3%	99,3%	98,5%	98,5%	99,5%	98,5%	98,1%	98,5%	97,7%	98,3%	98,5%	98,5%	100,0%	98,1%	98,3%	98,5%	97,5%
Bradyrhizobium sp. ZAR12	100,0%	99,5%	98,9%	100,0%	100,0%	ID	99,3%	99,3%	98,5%	98,5%	99,5%	98,5%	98,1%	98,5%	97,7%	98,3%	98,5%	98,5%	100,0%	98,1%	98,3%	98,5%	97,5%
Bradyrhizobium sp. SZCZ11	99,3%	98,9%	99,3%	99,3%	99,3%	99,3%	ID	100,0%	98,5%	98,5%	99,3%	98,5%	98,1%	99,1%	97,2%	98,3%	99,1%	98,5%	99,3%	98,1%	98,9%	99,1%	98,1%
Bradyrhizobium sp. SZCZ20	99,3%	98,9%	99,3%	99,3%	99,3%	99,3%	100,0%	ID	98,5%	98,5%	99,3%	98,5%	98,1%	99,1%	97,2%	98,3%	99,1%	98,5%	99,3%	98,1%	98,9%	99,1%	98,1%
B. japonicum USDA6	98,5%	98,5%	98,7%	98,5%	98,5%	98,5%	98,5%	98,5%	ID	100,0%	98,3%	100,0%	99,5%	97,7%	97,9%	99,7%	97,7%	100,0%	98,5%	98,7%	97,5%	97,7%	97,7%
B. japonicum E109	98,5%	98,5%	98,7%	98,5%	98,5%	98,5%	98,5%	98,5%	100,0%	ID	98,3%	100,0%	99,5%	97,7%	97,9%	99,7%	97,7%	100,0%	98,5%	98,7%	97,5%	97,7%	97,7%
B. japonicum J5	99,5%	99,1%	98,7%	99,5%	99,5%	99,5%	99,3%	99,3%	98,3%	98,3%	ID	98,3%	97,9%	98,5%	97,5%	98,1%	98,5%	98,3%	99,5%	98,1%	98,3%	98,5%	97,5%
B. japonicum SEMIA5079	98,5%	98,5%	98,7%	98,5%	98,5%	98,5%	98,5%	98,5%	100,0%	100,0%	98,3%	ID	99,5%	97,7%	97,9%	99,7%	97,7%	100,0%	98,5%	98,7%	97,5%	97,7%	97,7%
B. japonicum USDA38	98,1%	98,1%	98,3%	98,1%	98,1%	98,1%	98,1%	98,1%	99,5%	99,5%	97,9%	99,5%	ID	97,2%	97,5%	99,3%	97,2%	99,5%	98,1%	99,1%	97,0%	97,2%	97,7%
B. japonicum CCBAU15517	98,5%	98,1%	98,5%	98,5%	98,5%	98,5%	99,1%	99,1%	97,7%	97,7%	98,5%	97,7%	97,2%	ID	96,8%	97,5%	100,0%	97,7%	98,5%	97,7%	99,7%	100,0%	97,2%
B. japonicum CCBAU15618	97,7%	97,7%	97,5%	97,7%	97,7%	97,7%	97,2%	97,2%	97,9%	97,9%	97,5%	97,9%	97,5%	96,8%	ID	97,7%	96,8%	97,9%	97,7%	96,6%	96,6%	96,8%	96,2%
B. japonicum CCBAU25435	98,3%	98,3%	98,5%	98,3%	98,3%	98,3%	98,3%	98,3%	99,7%	99,7%	98,1%	99,7%	99,3%	97,5%	97,7%	ID	97,5%	99,7%	98,3%	98,5%	97,2%	97,5%	97,5%
B. japonicum CCBAU83623	98,5%	98,1%	98,5%	98,5%	98,5%	98,5%	99,1%	99,1%	97,7%	97,7%	98,5%	97,7%	97,2%	100,0%	96,8%	97,5%	ID	97,7%	98,5%	97,7%	99,7%	100,0%	97,2%
B. japonicum FN1	98,5%	98,5%	98,7%	98,5%	98,5%	98,5%	98,5%	98,5%	100,0%	100,0%	98,3%	100,0%	99,5%	97,7%	97,9%	99,7%	97,7%	ID	98,5%	98,7%	97,5%	97,7%	97,7%
B. japonicum UBMA197	100,0%	99,5%	98,9%	100,0%	100,0%	100,0%	99,3%	99,3%	98,5%	98,5%	99,5%	98,5%	98,1%	98,5%	97,7%	98,3%	98,5%	98,5%	ID	98,1%	98,3%	98,5%	97,5%
B. japonicum Is-34	98,1%	97,7%	97,9%	98,1%	98,1%	98,1%	98,1%	98,1%	98,7%	98,7%	98,1%	98,7%	99,1%	97,7%	96,6%	98,5%	97,7%	98,7%	98,1%	ID	97,5%	97,7%	97,2%
B. japonicum USDA123	98,3%	97,9%	98,3%	98,3%	98,3%	98,3%	98,9%	98,9%	97,5%	97,5%	98,3%	97,5%	97,0%	99,7%	96,6%	97,2%	99,7%	97,5%	98,3%	97,5%	ID	99,7%	97,0%
B. japonicum CCBAU15354	98,5%	98,1%	98,5%	98,5%	98,5%	98,5%	99,1%	99,1%	97,7%	97,7%	98,5%	97,7%	97,2%	100,0%	96,8%	97,5%	100,0%	97,7%	98,5%	97,7%	99,7%	ID	97,2%
B. diazoefficiens NK6	97,5%	97,5%	97,5%	97,5%	97,5%	97,5%	98,1%	98,1%	97,7%	97,7%	97,5%	97,7%	97,7%	97,2%	96,2%	97,5%	97,2%	97,7%	97,5%	97,2%	97,0%	97,2%	ID
B. diazoefficiens USDA122	97,2%	97,2%	97,2%	97,2%	97,2%	97,2%	97,9%	97,9%	97,5%	97,5%	97,2%	97,5%	97,5%	97,0%	96,0%	97,2%	97,0%	97,5%	97,2%	97,0%	96,8%	97,0%	99,7%
B. diazoefficiens SEMIA5080	97,2%	97,2%	97,2%	97,2%	97,2%	97,2%	97,9%	97,9%	97,5%	97,5%	97,2%	97,5%	97,5%	97,0%	96,0%	97,2%	97,0%	97,5%	97,2%	97,0%	96,8%	97,0%	99,7%
B. diazoefficiens USDA110	97,0%	97,0%	97,0%	97,0%	97,0%	97,0%	97,7%	97,7%	97,2%	97,2%	97,0%	97,2%	97,2%	96,8%	95,8%	97,0%	96,8%	97,2%	97,0%	96,8%	96,6%	96,8%	99,5%
B. japonicum Is-1	97,2%	97,2%	97,2%	97,2%	97,2%	97,2%	97,9%	97,9%	97,5%	97,5%	97,2%	97,5%	97,5%	97,0%	96,0%	97,2%	97,0%	97,5%	97,2%	97,0%	96,8%	97,0%	99,7%
B. japonicum USDA124	96,0%	95,8%	95,8%	96,0%	96,0%	96,0%	96,4%	96,4%	95,8%	95,8%	96,0%	95,8%	95,8%	96,0%	95,2%	95,6%	96,0%	95,8%	96,0%	95,6%	95,8%	96,0%	96,0%
B. huanghuaihaiense CCBAU23303	96,0%	95,8%	95,8%	96,0%	96,0%	96,0%	96,4%	96,4%	95,8%	95,8%	96,0%	95,8%	95,8%	96,0%	95,2%	95,6%	96,0%	95,8%	96,0%	95,6%	95,8%	96,0%	96,0%
B. japonicum in8p8	92,2%	91,8%	92,5%	92,2%	92,2%	92,2%	92,7%	92,7%	91,8%	91,8%	92,0%	91,8%	91,8%	91,8%	91,6%	91,6%	91,8%	91,8%	92,2%	91,8%	91,6%	91,8%	92,5%
B. japonicum is5	92,2%	91,8%	92,5%	92,2%	92,2%	92,2%	92,7%	92,7%	91,8%	91,8%	92,0%	91,8%	91,8%	91,8%	91,6%	91,6%	91,8%	91,8%	92,2%	91,8%	91,6%	91,8%	92,5%
B. japonicum 22	94,1%	93,7%	94,3%	94,1%	94,1%	94,1%	94,5%	94,5%	93,7%	93,7%	93,9%	93,7%	93,7%	93,7%	93,3%	93,5%	93,7%	93,7%	94,1%	93,7%	93,5%	93,7%	94,7%
B. yuanmingense CCBAU10071	93,5%	93,1%	93,7%	93,5%	93,5%	93,5%	93,9%	93,9%	93,1%	93,1%	93,3%	93,1%	93,1%	93,5%	93,3%	92,9%	93,5%	93,1%	93,5%	93,1%	93,3%	93,5%	93,7%
B. yuanmingense CCBAU05623	93,5%	93,1%	93,7%	93,5%	93,5%	93,5%	93,9%	93,9%	93,1%	93,1%	93,3%	93,1%	93,1%	93,5%	93,3%	92,9%	93,5%	93,1%	93,5%	93,1%	93,3%	93,5%	93,7%
B. yuanmingense BR3267	93,7%	93,3%	93,9%	93,7%	93,7%	93,7%	94,1%	94,1%	93,3%	93,3%	93,5%	93,3%	93,3%	93,7%	93,5%	93,1%	93,7%	93,3%	93,7%	93,3%	93,5%	93,7%	93,9%
B. yuanmingense CCBAU25021	93,5%	93,1%	93,7%	93,5%	93,5%	93,5%	93,9%	93,9%	93,1%	93,1%	93,3%	93,1%	93,1%	93,5%	93,3%	92,9%	93,5%	93,1%	93,5%	93,1%	93,3%	93,5%	93,7%
B. yuanmingense CCBAU35157	93,3%	92,9%	93,5%	93,3%	93,3%	93,3%	93,7%	93,7%	92,9%	92,9%	93,1%	92,9%	93,3%	93,3%	93,1%	92,7%	93,3%	92,9%	93,3%	93,3%	93,1%	93,3%	93,5%
B. canariense BTA-1	96,0%	95,8%	95,4%	96,0%	96,0%	96,0%	95,6%	95,6%	95,2%	95,2%	95,8%	95,2%	95,2%	95,2%	96,0%	95,0%	95,2%	95,2%	96,0%	95,0%	95,0%	95,2%	95,2%
B. canariense UBMA510	95,4%	95,0%	94,7%	95,4%	95,4%	95,4%	95,2%	95,2%	93,9%	93,9%	95,0%	93,9%	93,9%	94,7%	94,3%	93,7%	94,7%	93,9%	95,4%	93,9%	94,5%	94,7%	94,7%
B. canariense UBMA183	95,4%	95,0%	94,7%	95,4%	95,4%	95,4%	95,2%	95,2%	93,9%	93,9%	95,0%	93,9%	93,9%	94,7%	94,3%	93,7%	94,7%	93,9%	95,4%	93,9%	94,5%	94,7%	94,7%
B. canariense UBMA195	95,4%	95,0%	94,7%	95,4%	95,4%	95,4%	95,2%	95,2%	93,9%	93,9%	95,0%	93,9%	93,9%	94,7%	94,3%	93,7%	94,7%	93,9%	95,4%	93,9%	94,5%	94,7%	94,7%
B. canariense UBMAN05	95,4%	95,0%	94,7%	95,4%	95,4%	95,4%	95,2%	95,2%	93,9%	93,9%	95,0%	93,9%	93,9%	94,7%	94,3%	93,7%	94,7%	93,9%	95,4%	93,9%	94,5%	94,7%	94,7%
B. liaoningense LMG18230	95,6%	95,2%	96,2%	95,6%	95,6%	95,6%	96,0%	96,0%	95,6%	95,6%	95,4%	95,6%	95,6%	95,6%	95,2%	95,4%	95,6%	95,6%	95,6%	95,2%	95,4%	95,6%	96,0%
B. liaoningense CCNWSX0360	94,3%	93,9%	94,1%	94,3%	94,3%	94,3%	94,3%	94,3%	93,7%	93,7%	94,1%	93,7%	93,7%	93,5%	93,3%	93,5%	93,5%	93,7%	94,3%	93,7%	93,3%	93,5%	93,7%
B. liaoningense CCBAU83689	95,6%	95,2%	96,2%	95,6%	95,6%	95,6%	96,0%	96,0%	95,6%	95,6%	95,4%	95,6%	95,6%	95,6%	95,2%	95,4%	95,6%	95,6%	95,6%	95,2%	95,4%	95,6%	96,0%
B. liaoningense CCBAU05525	95,6%	95,2%	96,2%	95,6%	95,6%	95,6%	96,0%	96,0%	95,6%	95,6%	95,4%	95,6%	95,6%	95,6%	95,2%	95,4%	95,6%	95,6%	95,6%	95,2%	95,4%	95,6%	96,0%
B. japonicum USDA4	94,7%	94,3%	94,3%	94,7%	94,7%	94,7%	95,0%	95,0%	94,1%	94,1%	94,7%	94,1%	94,1%	94,3%	93,3%	93,9%	94,3%	94,1%	94,7%	94,1%	94,1%	94,3%	96,4%
B. japonicum USDA135	95,4%	95,0%	96,0%	95,4%	95,4%	95,4%	95,8%	95,8%	95,4%	95,4%	95,2%	95,4%	95,4%	95,4%	95,0%	95,2%	95,4%	95,4%	95,4%	95,0%	95,2%	95,4%	95,8%
B. ottawaense OO99	94,7%	94,3%	94,3%	94,7%	94,7%	94,7%	95,0%	95,0%	94,1%	94,1%	94,7%	94,1%	94,1%	94,3%	93,3%	93,9%	94,3%	94,1%	94,7%	94,1%	94,1%	94,3%	96,4%
B. daqingense CCBAU15774	93,9%	93,9%	93,5%	93,9%	93,9%	93,9%	93,7%	93,7%	93,9%	93,9%	93,7%	93,9%	93,9%	93,3%	94,1%	93,7%	93,3%	93,9%	93,9%	93,5%	93,1%	93,3%	94,7%
B. cytisi CTAW11	94,1%	94,1%	94,3%	94,1%	94,1%	94,1%	94,5%	94,5%	94,3%	94,3%	93,9%	94,3%	94,3%	93,7%	93,9%	94,1%	93,7%	94,3%	94,1%	93,9%	93,5%	93,7%	95,0%
B. rifense CTAW71	94,5%	94,5%	94,7%	94,5%	94,5%	94,5%	95,0%	95,0%	94,7%	94,7%	94,3%	94,7%	94,7%	94,1%	94,3%	94,5%	94,1%	94,7%	94,5%	94,3%	94,1%	94,1%	95,6%
B. ganzhouense RITF806	95,8%	95,8%	95,8%	95,8%	95,8%	95,8%	96,4%	96,4%	95,8%	95,8%	95,8%	95,8%	95,8%	95,6%	95,4%	95,6%	95,6%	95,8%	95,8%	95,4%	95,4%	95,6%	97,0%
B. arachidis CCBAU051107	95,8%	95,4%	96,0%	95,8%	95,8%	95,8%	96,2%	96,2%	95,4%	95,4%	95,6%	95,4%	95,4%	95,4%	94,7%	95,2%	95,4%	95,4%	95,8%	95,4%	95,2%	95,4%	96,0%
B. manausense BR3351	93,1%	92,7%	93,3%	93,1%	93,1%	93,1%	93,5%	93,5%	93,3%	93,3%	93,3%	93,3%	93,3%	93,1%	93,1%	93,1%	93,1%	93,3%	93,1%	93,9%	93,1%	93,1%	93,3%
B. neotropicale BR10247	93,9%	93,9%	93,7%	93,9%	93,9%	93,9%	93,9%	93,9%	93,9%	93,9%	93,7%	93,9%	93,5%	93,3%	93,9%	93,7%	93,3%	93,9%	93,9%	93,1%	93,1%	93,3%	93,9%
B. stylosanthis BR446	96,0%	96,0%	95,2%	96,0%	96,0%	96,0%	95,8%	95,8%	96,0%	96,0%	96,0%	96,0%	96,0%	95,0%	95,6%	95,8%	95,0%	96,0%	96,0%	95,6%	94,7%	95,0%	96,0%
B. pachyrhizi PAC48	87,9%	87,7%	87,9%	87,9%	87,9%	87,9%	88,5%	88,5%	87,9%	87,9%	87,9%	87,9%	87,9%	88,1%	87,2%	88,1%	88,1%	87,9%	87,9%	87,7%	87,9%	88,1%	89,3%
B. paxllaeri LMTR21	88,3%	88,1%	88,3%	88,3%	88,3%	88,3%	88,9%	88,9%	88,9%	88,9%	88,3%	88,9%	88,9%	88,3%	87,9%	89,1%	88,3%	88,9%	88,3%	88,7%	88,1%	88,3%	89,5%
B. icense LMTR13	88,7%	88,5%	88,5%	88,7%	88,7%	88,7%	89,1%	89,1%	89,3%	89,3%	88,7%	89,3%	89,3%	88,3%	88,5%	89,1%	88,3%	89,3%	88,7%	89,1%	88,1%	88,3%	90,2%
B. jicamae PAC68	87,9%	87,7%	87,9%	87,9%	87,9%	87,9%	88,5%	88,5%	88,5%	88,5%	87,9%	88,5%	88,5%	87,9%	87,0%	88,7%	87,9%	88,5%	87,9%	88,3%	87,7%	87,9%	89,5%
B. valentinum LmjM3	85,6%	85,8%	85,6%	85,6%	85,6%	85,6%	86,2%	86,2%	85,6%	85,6%	86,0%	85,6%	85,8%	85,6%	85,0%	85,4%	85,6%	85,6%	85,6%	85,8%	85,4%	85,6%	87,5%
B. retamae Ro19	87,9%	87,7%	87,7%	87,9%	87,9%	87,9%	88,3%	88,3%	88,3%	88,3%	87,9%	88,3%	88,3%	87,5%	86,8%	88,1%	87,5%	88,3%	87,9%	88,1%	87,2%	87,5%	89,1%
B. elkanii USDA76	88,3%	88,1%	88,5%	88,3%	88,3%	88,3%	88,7%	88,7%	88,5%	88,5%	88,5%	88,5%	88,5%	88,7%	88,3%	88,3%	88,7%	88,5%	88,3%	88,9%	88,5%	88,7%	89,5%
B. embrapense SEMIA6208	88,7%	88,7%	88,7%	88,7%	88,7%	88,7%	88,9%	88,9%	89,1%	89,1%	88,5%	89,1%	89,1%	88,5%	88,9%	88,9%	88,5%	89,1%	88,7%	88,7%	88,3%	88,5%	90,0%
B. erythrophlei CCBAU53325	88,7%	88,5%	88,9%	88,7%	88,7%	88,7%	89,1%	89,1%	89,1%	89,1%	88,9%	89,1%	89,1%	88,7%	88,3%	88,9%	88,7%	89,1%	88,7%	89,1%	88,5%	88,7%	90,0%
B. viridifuturi SEMIA690	88,1%	87,9%	87,7%	88,1%	88,1%	88,1%	87,9%	87,9%	87,7%	87,7%	88,3%	87,7%	87,7%	87,5%	87,9%	87,5%	87,5%	87,7%	88,1%	87,7%	87,2%	87,5%	88,3%
B. mercantei SEMIA6399	88,3%	88,1%	87,9%	88,3%	88,3%	88,3%	88,5%	88,5%	88,1%	88,1%	88,3%	88,1%	88,1%	87,7%	87,2%	87,9%	87,7%	88,1%	88,3%	87,9%	87,5%	87,7%	89,3%
B. lablabi CCBAU23086	88,3%	88,1%	88,3%	88,3%	88,3%	88,3%	88,9%	88,9%	88,9%	88,9%	88,7%	88,9%	88,9%	88,3%	88,3%	89,1%	88,3%	88,9%	88,3%	88,9%	88,1%	88,3%	89,1%
Rhodopseudomonas plaustris CGA009	83,1%	82,9%	83,3%	83,1%	83,1%	83,1%	83,5%	83,5%	83,5%	83,5%	82,9%	83,5%	83,1%	82,7%	83,3%	83,5%	82,7%	83,5%	83,1%	82,9%	82,5%	82,7%	83,5%

	B. diazoefficiens USDA122	*B. diazoefficiens* SEMIA5080	*B. diazoefficiens* USDA110	*B. japonicum* Is-1	*B. japonicum* USDA124	*B. huanghuaihaiense* CCBAU23303	*B. japonicum* in8p8	*B. japonicum* is5	*B. japonicum* 22	*B. yuanmingense* CCBAU10071	*B. yuanmingense* CCBAU05623	*B. yuanmingense* BR3267	*B. yuanmingense* CCBAU25021	*B. yuanmingense* CCBAU35157	*B. canariense* BTA-1	*B. canariense* UBMA510	*B. canariense* UBMA183	*B. canariense* UBMA195	*B. canariense* UBMAN05	*B. liaoningense* LMG18230	*B. liaoningense* CCNWSX0360	*B. liaoningense* CCBAU83689	*B. liaoningense* CCBAU05525
Bradyrhizobium sp. JAN6	97,2%	97,2%	97,0%	97,2%	96,0%	96,0%	92,2%	92,2%	94,1%	93,5%	93,5%	93,7%	93,5%	93,3%	96,0%	95,4%	95,4%	95,4%	95,4%	95,6%	94,3%	95,6%	95,6%
Bradyrhizobium sp. JAN10	97,2%	97,2%	97,0%	97,2%	95,8%	95,8%	91,8%	91,8%	93,7%	93,1%	93,1%	93,3%	93,1%	92,9%	95,8%	95,0%	95,0%	95,0%	95,0%	95,2%	93,9%	95,2%	95,2%
Bradyrhizobium sp. GER1	97,2%	97,2%	97,0%	97,2%	95,8%	95,8%	92,5%	92,5%	94,3%	93,7%	93,7%	93,9%	93,7%	93,5%	95,4%	94,7%	94,7%	94,7%	94,7%	96,2%	94,1%	96,2%	96,2%
Bradyrhizobium sp. GER32	97,2%	97,2%	97,0%	97,2%	96,0%	96,0%	92,2%	92,2%	94,1%	93,5%	93,5%	93,7%	93,5%	93,3%	96,0%	95,4%	95,4%	95,4%	95,4%	95,6%	94,3%	95,6%	95,6%
Bradyrhizobium sp. ZAR4	97,2%	97,2%	97,0%	97,2%	96,0%	96,0%	92,2%	92,2%	94,1%	93,5%	93,5%	93,7%	93,5%	93,3%	96,0%	95,4%	95,4%	95,4%	95,4%	95,6%	94,3%	95,6%	95,6%
Bradyrhizobium sp. ZAR12	97,2%	97,2%	97,0%	97,2%	96,0%	96,0%	92,2%	92,2%	94,1%	93,5%	93,5%	93,7%	93,5%	93,3%	96,0%	95,4%	95,4%	95,4%	95,4%	95,6%	94,3%	95,6%	95,6%
Bradyrhizobium sp. SZCZ11	97,9%	97,9%	97,7%	97,9%	96,4%	96,4%	92,7%	92,7%	94,5%	93,9%	93,9%	94,1%	93,9%	93,7%	95,6%	95,2%	95,2%	95,2%	95,2%	96,0%	94,3%	96,0%	96,0%
Bradyrhizobium sp. SZCZ20	97,9%	97,9%	97,7%	97,9%	96,4%	96,4%	92,7%	92,7%	94,5%	93,9%	93,9%	94,1%	93,9%	93,7%	95,6%	95,2%	95,2%	95,2%	95,2%	96,0%	94,3%	96,0%	96,0%
B. japonicum USDA6	97,5%	97,5%	97,2%	97,5%	95,8%	95,8%	91,8%	91,8%	93,7%	93,1%	93,1%	93,3%	93,1%	92,9%	95,2%	93,9%	93,9%	93,9%	93,9%	95,6%	93,7%	95,6%	95,6%
B. japonicum E109	97,5%	97,5%	97,2%	97,5%	95,8%	95,8%	91,8%	91,8%	93,7%	93,1%	93,1%	93,3%	93,1%	92,9%	95,2%	93,9%	93,9%	93,9%	93,9%	95,6%	93,7%	95,6%	95,6%
B. japonicum J5	97,2%	97,2%	97,0%	97,2%	96,0%	96,0%	92,0%	92,0%	93,9%	93,3%	93,3%	93,5%	93,3%	93,1%	95,8%	95,0%	95,0%	95,0%	95,0%	95,4%	94,1%	95,4%	95,4%
B. japonicum SEMIA5079	97,5%	97,5%	97,2%	97,5%	95,8%	95,8%	91,8%	91,8%	93,7%	93,1%	93,1%	93,3%	93,1%	92,9%	95,2%	93,9%	93,9%	93,9%	93,9%	95,6%	93,7%	95,6%	95,6%
B. japonicum USDA38	97,5%	97,5%	97,2%	97,5%	95,8%	95,8%	91,8%	91,8%	93,7%	93,1%	93,1%	93,3%	93,1%	93,3%	95,2%	93,9%	93,9%	93,9%	93,9%	95,6%	93,7%	95,6%	95,6%
B. japonicum CCBAU15517	97,0%	97,0%	96,8%	97,0%	96,0%	96,0%	91,8%	91,8%	93,7%	93,5%	93,5%	93,7%	93,5%	93,3%	95,2%	94,7%	94,7%	94,7%	94,7%	95,6%	93,5%	95,6%	95,6%
B. japonicum CCBAU15618	96,0%	96,0%	95,8%	96,0%	95,2%	95,2%	91,6%	91,6%	93,3%	93,3%	93,3%	93,5%	93,3%	93,1%	96,0%	94,3%	94,3%	94,3%	94,3%	95,2%	93,3%	95,2%	95,2%
B. japonicum CCBAU25435	97,2%	97,2%	97,0%	97,2%	95,6%	95,6%	91,6%	91,6%	93,5%	92,9%	92,9%	93,1%	92,9%	92,7%	95,0%	93,7%	93,7%	93,7%	93,7%	95,4%	93,5%	95,4%	95,4%
B. japonicum CCBAU83623	97,0%	97,0%	96,8%	97,0%	96,0%	96,0%	91,8%	91,8%	93,7%	93,5%	93,5%	93,7%	93,5%	93,3%	95,2%	94,7%	94,7%	94,7%	94,7%	95,6%	93,5%	95,6%	95,6%
B. japonicum FN1	97,5%	97,5%	97,2%	97,5%	95,8%	95,8%	91,8%	91,8%	93,7%	93,1%	93,1%	93,3%	93,1%	92,9%	95,2%	93,9%	93,9%	93,9%	93,9%	95,6%	93,7%	95,6%	95,6%
B. japonicum UBMA197	97,2%	97,2%	97,0%	97,2%	96,0%	96,0%	92,2%	92,2%	94,1%	93,5%	93,5%	93,7%	93,5%	93,3%	96,0%	95,4%	95,4%	95,4%	95,4%	95,6%	94,3%	95,6%	95,6%
B. japonicum Is-34	97,0%	97,0%	96,8%	97,0%	95,6%	95,6%	91,8%	91,8%	93,7%	93,1%	93,1%	93,3%	93,1%	93,3%	95,0%	93,9%	93,9%	93,9%	93,9%	95,2%	93,7%	95,2%	95,2%
B. japonicum USDA123	96,8%	96,8%	96,6%	96,8%	95,8%	95,8%	91,6%	91,6%	93,5%	93,3%	93,3%	93,5%	93,3%	93,1%	95,0%	94,5%	94,5%	94,5%	94,5%	95,4%	93,3%	95,4%	95,4%
B. japonicum CCBAU15354	97,0%	97,0%	96,8%	97,0%	96,0%	96,0%	91,8%	91,8%	93,7%	93,5%	93,5%	93,7%	93,5%	93,3%	95,2%	94,7%	94,7%	94,7%	94,7%	95,6%	93,5%	95,6%	95,6%
B. diazoefficiens NK6	99,7%	99,7%	99,5%	99,7%	96,0%	96,0%	92,5%	92,5%	94,7%	93,7%	93,7%	93,9%	93,7%	93,5%	95,2%	94,7%	94,7%	94,7%	94,7%	96,0%	93,7%	96,0%	96,0%
B. diazoefficiens USDA122	ID	100,0%	99,3%	100,0%	95,8%	95,8%	92,2%	92,2%	94,5%	93,5%	93,5%	93,7%	93,5%	93,3%	95,0%	94,5%	94,5%	94,5%	94,5%	95,8%	93,5%	95,8%	95,8%
B. diazoefficiens SEMIA5080	100,0%	ID	99,3%	100,0%	95,8%	95,8%	92,2%	92,2%	94,5%	93,5%	93,5%	93,7%	93,5%	93,3%	95,0%	94,5%	94,5%	94,5%	94,5%	95,8%	93,5%	95,8%	95,8%
B. diazoefficiens USDA110	99,3%	99,3%	ID	99,3%	95,6%	95,6%	92,0%	92,0%	94,3%	93,3%	93,3%	93,5%	93,3%	93,1%	94,7%	94,3%	94,3%	94,3%	94,3%	95,6%	93,3%	95,6%	95,6%
B. japonicum Is-1	100,0%	100,0%	99,3%	ID	95,8%	95,8%	92,2%	92,2%	94,5%	93,5%	93,5%	93,7%	93,5%	93,3%	95,0%	94,5%	94,5%	94,5%	94,5%	95,8%	93,5%	95,8%	95,8%
B. japonicum USDA124	95,8%	95,8%	95,6%	95,8%	ID	100,0%	92,9%	92,9%	94,5%	93,9%	93,9%	94,1%	93,9%	93,7%	95,0%	94,1%	94,1%	94,1%	94,1%	94,1%	94,5%	94,1%	94,1%
B. huanghuaihaiense CCBAU23303	95,8%	95,8%	95,6%	95,8%	100,0%	ID	92,9%	92,9%	94,5%	93,9%	93,9%	94,1%	93,9%	93,7%	95,0%	94,1%	94,1%	94,1%	94,1%	94,1%	94,5%	94,1%	94,1%
B. japonicum in8p8	92,2%	92,2%	92,0%	92,2%	92,9%	92,9%	ID	100,0%	95,2%	93,9%	93,9%	94,1%	93,9%	93,7%	92,9%	92,0%	92,0%	92,0%	92,0%	92,5%	93,3%	92,5%	92,5%
B. japonicum is5	92,2%	92,2%	92,0%	92,2%	92,9%	92,9%	100,0%	ID	95,2%	93,9%	93,9%	94,1%	93,9%	93,7%	92,9%	92,0%	92,0%	92,0%	92,0%	92,5%	93,3%	92,5%	92,5%
B. japonicum 22	94,5%	94,5%	94,3%	94,5%	94,5%	94,5%	95,2%	95,2%	ID	92,7%	92,7%	92,9%	92,7%	92,5%	93,5%	92,7%	92,7%	92,7%	92,7%	94,3%	93,9%	94,3%	94,3%
B. yuanmingense CCBAU10071	93,5%	93,5%	93,3%	93,5%	93,9%	93,9%	93,9%	93,9%	92,7%	ID	100,0%	99,7%	100,0%	99,7%	93,5%	93,3%	93,3%	93,3%	93,3%	94,5%	94,3%	94,5%	94,5%
B. yuanmingense CCBAU05623	93,5%	93,5%	93,3%	93,5%	93,9%	93,9%	93,9%	93,9%	92,7%	100,0%	ID	99,7%	100,0%	99,7%	93,5%	93,3%	93,3%	93,3%	93,3%	94,5%	94,3%	94,5%	94,5%
B. yuanmingense BR3267	93,7%	93,7%	93,5%	93,7%	94,1%	94,1%	94,1%	94,1%	92,9%	99,7%	99,7%	ID	99,7%	99,5%	93,7%	93,5%	93,5%	93,5%	93,5%	94,7%	94,5%	94,7%	94,7%
B. yuanmingense CCBAU25021	93,5%	93,5%	93,3%	93,5%	93,9%	93,9%	93,9%	93,9%	92,7%	100,0%	100,0%	99,7%	ID	99,7%	93,5%	93,3%	93,3%	93,3%	93,3%	94,5%	94,3%	94,5%	94,5%
B. yuanmingense CCBAU35157	93,3%	93,3%	93,1%	93,3%	93,7%	93,7%	93,7%	93,7%	92,5%	99,7%	99,7%	99,5%	99,7%	ID	93,3%	93,1%	93,1%	93,1%	93,1%	94,3%	94,1%	94,3%	94,3%
B. canariense BTA-1	95,0%	95,0%	94,7%	95,0%	95,0%	95,0%	92,9%	92,9%	93,5%	93,5%	93,5%	93,7%	93,5%	93,3%	ID	97,0%	97,0%	97,0%	97,0%	94,7%	93,9%	94,7%	94,7%
B. canariense UBMA510	94,5%	94,5%	94,3%	94,5%	94,1%	94,1%	92,0%	92,0%	92,7%	93,3%	93,3%	93,5%	93,3%	93,1%	97,0%	ID	100,0%	100,0%	100,0%	94,3%	93,7%	94,3%	94,3%
B. canariense UBMA183	94,5%	94,5%	94,3%	94,5%	94,1%	94,1%	92,0%	92,0%	92,7%	93,3%	93,3%	93,5%	93,3%	93,1%	97,0%	100,0%	ID	100,0%	100,0%	94,3%	93,7%	94,3%	94,3%
B. canariense UBMA195	94,5%	94,5%	94,3%	94,5%	94,1%	94,1%	92,0%	92,0%	92,7%	93,3%	93,3%	93,5%	93,3%	93,1%	97,0%	100,0%	100,0%	ID	100,0%	94,3%	93,7%	94,3%	94,3%
B. canariense UBMAN05	94,5%	94,5%	94,3%	94,5%	94,1%	94,1%	92,0%	92,0%	92,7%	93,3%	93,3%	93,5%	93,3%	93,1%	97,0%	100,0%	100,0%	100,0%	ID	94,3%	93,7%	94,3%	94,3%
B. liaoningense LMG18230	95,8%	95,8%	95,6%	95,8%	94,1%	94,1%	92,5%	92,5%	94,3%	94,5%	94,5%	94,7%	94,5%	94,3%	94,7%	94,3%	94,3%	94,3%	94,3%	ID	94,7%	100,0%	100,0%
B. liaoningense CCNWSX0360	93,5%	93,5%	93,3%	93,5%	94,5%	94,5%	93,3%	93,3%	93,9%	94,3%	94,3%	94,5%	94,3%	94,1%	93,9%	93,7%	93,7%	93,7%	93,7%	94,7%	ID	94,7%	94,7%
B. liaoningense CCBAU83689	95,8%	95,8%	95,6%	95,8%	94,1%	94,1%	92,5%	92,5%	94,3%	94,5%	94,5%	94,7%	94,5%	94,3%	94,7%	94,3%	94,3%	94,3%	94,3%	100,0%	94,7%	ID	100,0%
B. liaoningense CCBAU05525	95,8%	95,8%	95,6%	95,8%	94,1%	94,1%	92,5%	92,5%	94,3%	94,5%	94,5%	94,7%	94,5%	94,3%	94,7%	94,3%	94,3%	94,3%	94,3%	100,0%	94,7%	100,0%	ID
B. japonicum USDA4	96,2%	96,2%	96,0%	96,2%	93,1%	93,1%	92,2%	92,2%	92,5%	94,1%	94,1%	94,3%	94,1%	93,9%	92,5%	92,2%	92,2%	92,2%	92,2%	93,9%	92,0%	93,9%	93,9%
B. japonicum USDA135	95,6%	95,6%	95,4%	95,6%	93,9%	93,9%	92,2%	92,2%	94,1%	94,3%	94,3%	94,5%	94,3%	94,1%	94,5%	94,1%	94,1%	94,1%	94,1%	99,7%	94,5%	99,7%	99,7%
B. ottawaense OO99	96,2%	96,2%	96,0%	96,2%	93,1%	93,1%	92,2%	92,2%	92,5%	94,1%	94,1%	94,3%	94,1%	93,9%	92,5%	92,2%	92,2%	92,2%	92,2%	93,9%	92,0%	93,9%	93,9%
B. daqingense CCBAU15774	94,5%	94,5%	94,3%	94,5%	93,3%	93,3%	93,7%	93,7%	92,2%	95,2%	95,2%	95,4%	95,2%	95,0%	93,7%	92,5%	92,5%	92,5%	92,5%	94,5%	92,5%	94,5%	94,5%
B. cytisi CTAW11	94,7%	94,7%	94,7%	94,7%	92,9%	92,9%	91,2%	91,2%	92,0%	92,5%	92,5%	92,7%	92,5%	92,2%	93,9%	92,7%	92,7%	92,7%	92,7%	93,9%	92,7%	93,9%	93,9%
B. rifense CTAW71	95,4%	95,4%	95,4%	95,4%	94,1%	94,1%	92,5%	92,5%	92,7%	92,9%	92,9%	93,1%	92,9%	92,7%	95,4%	94,5%	94,5%	94,5%	94,5%	94,7%	93,3%	94,7%	94,7%
B. ganzhouense RITF806	96,8%	96,8%	96,8%	96,8%	96,0%	96,0%	93,1%	93,1%	93,3%	94,1%	94,1%	94,3%	94,1%	93,9%	95,6%	95,2%	95,2%	95,2%	95,2%	95,0%	93,7%	95,0%	95,0%
B. arachidis CCBAU051107	95,8%	95,8%	95,6%	95,8%	93,9%	93,9%	93,9%	93,9%	94,1%	96,0%	96,0%	96,2%	96,0%	95,8%	94,3%	93,3%	93,3%	93,3%	93,3%	95,6%	93,7%	95,6%	95,6%
B. manausense BR3351	93,1%	93,1%	92,9%	93,1%	93,9%	93,9%	92,7%	92,7%	92,9%	94,5%	94,5%	94,7%	94,5%	94,3%	92,2%	91,4%	91,4%	91,4%	91,4%	93,1%	94,1%	93,1%	93,1%
B. neotropicale BR10247	93,7%	93,7%	93,7%	93,7%	91,8%	91,8%	91,6%	91,6%	91,6%	94,1%	94,1%	94,3%	94,1%	93,9%	93,3%	92,0%	92,0%	92,0%	92,0%	92,9%	92,5%	92,9%	92,9%
B. stylosanthis BR446	95,8%	95,8%	95,6%	95,8%	96,8%	96,8%	92,5%	92,5%	93,9%	93,1%	93,1%	93,3%	93,1%	92,9%	95,0%	93,9%	93,9%	93,9%	93,9%	94,5%	96,0%	94,5%	94,5%
B. pachyrhizi PAC48	89,1%	89,1%	89,3%	89,1%	87,5%	87,5%	87,7%	87,7%	88,7%	87,7%	87,7%	87,9%	87,7%	87,5%	86,4%	86,2%	86,2%	86,2%	86,2%	88,9%	87,0%	88,9%	88,9%
B. paxllaeri LMTR21	89,3%	89,3%	89,1%	89,3%	88,5%	88,5%	88,7%	88,7%	88,5%	89,5%	89,5%	89,7%	89,5%	89,3%	87,9%	86,4%	86,4%	86,4%	86,4%	88,7%	87,9%	88,7%	88,7%
B. icense LMTR13	90,0%	90,0%	89,7%	90,0%	89,7%	89,7%	90,0%	90,0%	89,1%	90,4%	90,4%	90,6%	90,4%	90,2%	87,9%	87,0%	87,0%	87,0%	87,0%	89,5%	88,9%	89,5%	89,5%
B. jicamae PAC68	89,3%	89,3%	89,1%	89,3%	88,1%	88,1%	89,3%	89,3%	88,3%	89,3%	89,3%	89,5%	89,3%	89,1%	87,2%	86,6%	86,6%	86,6%	86,6%	88,1%	87,7%	88,1%	88,1%
B. valentinum LmjM3	87,2%	87,2%	87,2%	87,2%	85,4%	85,4%	84,5%	84,5%	86,0%	84,7%	84,7%	84,5%	84,7%	84,7%	84,5%	84,1%	84,1%	84,1%	84,1%	86,0%	84,7%	86,0%	86,0%
B. retamae Ro19	88,9%	88,9%	88,7%	88,9%	89,1%	89,1%	89,1%	89,1%	88,5%	89,3%	89,3%	89,5%	89,3%	89,1%	87,2%	86,8%	86,8%	86,8%	86,8%	88,7%	88,5%	88,7%	88,7%
B. elkanii USDA76	89,3%	89,3%	89,5%	89,3%	88,1%	88,1%	88,9%	88,9%	89,7%	89,3%	89,3%	89,5%	89,3%	89,1%	87,7%	87,5%	87,5%	87,5%	87,5%	90,4%	88,7%	90,4%	90,4%
B. embrapense SEMIA6208	89,7%	89,7%	90,0%	89,7%	88,5%	88,5%	88,7%	88,7%	89,5%	89,1%	89,1%	89,3%	89,1%	88,9%	87,9%	87,2%	87,2%	87,2%	87,2%	90,2%	88,9%	90,2%	90,2%
B. erythrophlei CCBAU53325	89,7%	89,7%	90,0%	89,7%	89,1%	89,1%	88,9%	88,9%	89,3%	91,0%	91,0%	91,0%	91,0%	90,8%	88,3%	88,5%	88,5%	88,5%	88,5%	90,4%	89,5%	90,4%	90,4%
B. viridifuturi SEMIA690	88,1%	88,1%	88,3%	88,1%	88,7%	88,7%	87,5%	87,5%	88,1%	88,5%	88,5%	88,7%	88,5%	88,3%	86,8%	86,6%	86,6%	86,6%	86,6%	88,5%	87,5%	88,5%	88,5%
B. mercantei SEMIA6399	89,1%	89,1%	89,3%	89,1%	88,1%	88,1%	86,8%	86,8%	88,1%	89,1%	89,1%	88,9%	89,1%	88,9%	86,8%	86,6%	86,6%	86,6%	86,6%	89,7%	88,9%	89,7%	89,7%
B. lablabi CCBAU23086	88,9%	88,9%	88,7%	88,9%	88,7%	88,7%	88,1%	88,1%	87,5%	90,2%	90,2%	90,4%	90,2%	90,0%	87,5%	86,4%	86,4%	86,4%	86,4%	89,1%	88,3%	89,1%	89,1%
Rhodopseudomonas plaustris CGA009	83,3%	83,3%	83,1%	83,3%	83,5%	83,5%	85,4%	85,4%	84,1%	85,4%	85,4%	85,2%	85,4%	85,2%	82,7%	81,6%	81,6%	81,6%	81,6%	83,9%	85,4%	83,9%	83,9%

	B. japonicum USDA4	*B. japonicum* USDA135	*B. ottawaense* OO99	*B. daqingense* CCBAU15774	*B. cytisi* CTAW11	*B. rifense* CTAW71	*B. ganzhouense* RITF806	*B. arachidis* CCBAU051107	*B. manausense* BR3351	*B. neotropicale* BR10247	*B. stylosanthis* BR446	*B. pachyrhizi* PAC48	*B. paxllaeri* LMTR21	*B. icense* LMTR13	*B. jicamae* PAC68	*B. valentinum* LmjM3	*B. retamae* Ro19	*B. elkanii* USDA76	*B. embrapense* SEMIA6208	*B. erythrophlei* CCBAU53325	*B. viridifuturi* SEMIA690	*B. mercantei* SEMIA6399	*B. lablabi* CCBAU23086	*Rhodopseudomonas plaustris* CGA009
Bradyrhizobium sp. JAN6	94,7%	95,4%	94,7%	93,9%	94,1%	94,5%	95,8%	95,8%	93,1%	93,9%	96,0%	87,9%	88,3%	88,7%	87,9%	85,6%	87,9%	88,3%	88,7%	88,7%	88,1%	88,3%	88,3%	83,1%
Bradyrhizobium sp. JAN10	94,3%	95,0%	94,3%	93,9%	94,1%	94,5%	95,8%	95,4%	92,7%	93,9%	96,0%	87,7%	88,1%	88,5%	87,7%	85,8%	87,7%	88,1%	88,7%	88,5%	87,9%	88,1%	88,1%	82,9%
Bradyrhizobium sp. GER1	94,3%	96,0%	94,3%	93,5%	94,3%	94,7%	95,8%	96,0%	93,3%	93,7%	95,2%	87,9%	88,3%	88,5%	87,9%	85,6%	87,7%	88,5%	88,7%	88,9%	87,7%	87,9%	88,3%	83,3%
Bradyrhizobium sp. GER32	94,7%	95,4%	94,7%	93,9%	94,1%	94,5%	95,8%	95,8%	93,1%	93,9%	96,0%	87,9%	88,3%	88,7%	87,9%	85,6%	87,9%	88,3%	88,7%	88,7%	88,1%	88,3%	88,3%	83,1%
Bradyrhizobium sp. ZAR4	94,7%	95,4%	94,7%	93,9%	94,1%	94,5%	95,8%	95,8%	93,1%	93,9%	96,0%	87,9%	88,3%	88,7%	87,9%	85,6%	87,9%	88,3%	88,7%	88,7%	88,1%	88,3%	88,3%	83,1%
Bradyrhizobium sp. ZAR12	94,7%	95,4%	94,7%	93,9%	94,1%	94,5%	95,8%	95,8%	93,1%	93,9%	96,0%	87,9%	88,3%	88,7%	87,9%	85,6%	87,9%	88,3%	88,7%	88,7%	88,1%	88,3%	88,3%	83,1%
Bradyrhizobium sp. SZCZ11	95,0%	95,8%	95,0%	93,7%	94,5%	95,0%	96,4%	96,2%	93,5%	93,9%	95,8%	88,5%	88,9%	89,1%	88,5%	86,2%	88,3%	88,7%	88,9%	89,1%	87,9%	88,5%	88,9%	83,5%
Bradyrhizobium sp. SZCZ20	95,0%	95,8%	95,0%	93,7%	94,5%	95,0%	96,4%	96,2%	93,5%	93,9%	95,8%	88,5%	88,9%	89,1%	88,5%	86,2%	88,3%	88,7%	88,9%	89,1%	87,9%	88,5%	88,9%	83,5%
B. japonicum USDA6	94,1%	95,4%	94,1%	93,9%	94,3%	94,7%	95,8%	95,4%	93,3%	93,9%	96,0%	87,9%	88,9%	89,3%	88,5%	85,6%	88,3%	88,5%	89,1%	89,1%	87,7%	88,1%	88,9%	83,5%
B. japonicum E109	94,1%	95,4%	94,1%	93,9%	94,3%	94,7%	95,8%	95,4%	93,3%	93,9%	96,0%	87,9%	88,9%	89,3%	88,5%	85,6%	88,3%	88,5%	89,1%	89,1%	87,7%	88,1%	88,9%	83,5%
B. japonicum J5	94,7%	95,2%	94,7%	93,7%	93,9%	94,3%	95,8%	95,6%	93,3%	93,7%	96,0%	87,9%	88,3%	88,7%	87,9%	86,0%	87,9%	88,5%	88,5%	88,9%	88,3%	88,3%	88,7%	82,9%
B. japonicum SEMIA5079	94,1%	95,4%	94,1%	93,9%	94,3%	94,7%	95,8%	95,4%	93,3%	93,9%	96,0%	87,9%	88,9%	89,3%	88,5%	85,6%	88,3%	88,5%	89,1%	89,1%	87,7%	88,1%	88,9%	83,5%
B. japonicum USDA38	94,1%	95,4%	94,1%	93,9%	94,3%	94,7%	95,8%	95,4%	93,3%	93,5%	96,0%	87,9%	88,9%	89,3%	88,5%	85,8%	88,3%	88,5%	89,1%	89,1%	87,7%	88,1%	88,9%	83,1%
B. japonicum CCBAU15517	94,3%	95,4%	94,3%	93,3%	93,7%	94,1%	95,6%	95,4%	93,1%	93,3%	95,0%	88,1%	88,3%	88,3%	87,9%	85,6%	87,5%	88,7%	88,5%	88,7%	87,5%	87,7%	88,3%	82,7%
B. japonicum CCBAU15618	93,3%	95,0%	93,3%	94,1%	93,9%	94,3%	95,4%	94,7%	93,1%	93,9%	95,6%	87,2%	87,9%	88,5%	87,0%	85,0%	86,8%	88,3%	88,9%	88,3%	87,9%	87,2%	88,3%	83,3%
B. japonicum CCBAU25435	93,9%	95,2%	93,9%	93,7%	94,1%	94,5%	95,6%	95,2%	93,1%	93,7%	95,8%	88,1%	89,1%	89,1%	88,7%	85,4%	88,1%	88,3%	88,9%	88,9%	87,5%	87,9%	89,1%	83,5%
B. japonicum CCBAU83623	94,3%	95,4%	94,3%	93,3%	93,7%	94,1%	95,6%	95,4%	93,1%	93,3%	95,0%	88,1%	88,3%	88,3%	87,9%	85,6%	87,5%	88,7%	88,5%	88,7%	87,5%	87,7%	88,3%	82,7%
B. japonicum FN1	94,1%	95,4%	94,1%	93,9%	94,3%	94,7%	95,8%	95,4%	93,3%	93,9%	96,0%	87,9%	88,9%	89,3%	88,5%	85,6%	88,3%	88,5%	89,1%	89,1%	87,7%	88,1%	88,9%	83,5%
B. japonicum UBMA197	94,7%	95,4%	94,7%	93,9%	94,1%	94,5%	95,8%	95,8%	93,1%	93,9%	96,0%	87,9%	88,3%	88,7%	87,9%	85,6%	87,9%	88,3%	88,7%	88,7%	88,1%	88,3%	88,3%	83,1%
B. japonicum Is-34	94,1%	95,0%	94,1%	93,5%	93,9%	94,3%	95,4%	95,4%	93,9%	93,1%	95,6%	87,7%	88,7%	89,1%	88,3%	85,8%	88,1%	88,9%	88,7%	89,1%	87,7%	87,9%	88,9%	82,9%
B. japonicum USDA123	94,1%	95,2%	94,1%	93,1%	93,5%	94,1%	95,4%	95,2%	93,1%	93,1%	94,7%	87,9%	88,1%	88,1%	87,7%	85,4%	87,2%	88,5%	88,3%	88,5%	87,2%	87,5%	88,1%	82,5%
B. japonicum CCBAU15354	94,3%	95,4%	94,3%	93,3%	93,7%	94,1%	95,6%	95,4%	93,1%	93,3%	95,0%	88,1%	88,3%	88,3%	87,9%	85,6%	87,5%	88,7%	88,5%	88,7%	87,5%	87,7%	88,3%	82,7%
B. diazoefficiens NK6	96,4%	95,8%	96,4%	94,7%	95,0%	95,6%	97,0%	96,0%	93,3%	93,9%	96,0%	89,3%	89,5%	90,2%	89,5%	87,5%	89,1%	89,5%	90,0%	90,0%	88,3%	89,3%	89,1%	83,5%
B. diazoefficiens USDA122	96,2%	95,6%	96,2%	94,5%	94,7%	95,4%	96,8%	95,8%	93,1%	93,7%	95,8%	89,1%	89,3%	90,0%	89,3%	87,2%	88,9%	89,3%	89,7%	89,7%	88,1%	89,1%	88,9%	83,3%
B. diazoefficiens SEMIA5080	96,2%	95,6%	96,2%	94,5%	94,7%	95,4%	96,8%	95,8%	93,1%	93,7%	95,8%	89,1%	89,3%	90,0%	89,3%	87,2%	88,9%	89,3%	89,7%	89,7%	88,1%	89,1%	88,9%	83,3%
B. diazoefficiens USDA110	96,0%	95,4%	96,0%	94,3%	94,7%	95,4%	96,8%	95,6%	92,9%	93,7%	95,8%	89,3%	89,1%	89,7%	89,1%	87,2%	88,7%	89,5%	90,0%	90,0%	88,3%	89,3%	88,7%	83,1%
B. japonicum Is-1	96,2%	95,6%	96,2%	94,5%	94,7%	95,4%	96,8%	95,8%	93,1%	93,7%	95,8%	89,1%	89,3%	90,0%	89,3%	87,2%	88,9%	89,3%	89,7%	89,7%	88,1%	89,1%	88,9%	83,3%
B. japonicum USDA124	93,1%	93,9%	93,1%	93,3%	92,9%	94,1%	96,0%	93,9%	93,9%	91,8%	96,8%	87,5%	88,5%	89,7%	88,1%	85,4%	89,1%	88,1%	88,5%	89,1%	88,7%	88,1%	88,7%	83,5%
B. huanghuaihaiense CCBAU23303	93,1%	93,9%	93,1%	93,3%	92,9%	94,1%	96,0%	93,9%	93,9%	91,8%	96,8%	87,5%	88,5%	89,7%	88,1%	85,4%	89,1%	88,1%	88,5%	89,1%	88,7%	88,1%	88,7%	83,5%
B. japonicum in8p8	92,2%	92,2%	92,2%	93,7%	91,2%	92,5%	93,1%	93,9%	92,7%	91,6%	92,5%	87,7%	88,7%	90,0%	89,3%	84,5%	89,1%	88,9%	88,7%	88,9%	87,5%	86,8%	88,1%	85,4%
B. japonicum is5	92,2%	92,2%	92,2%	93,7%	91,2%	92,5%	93,1%	93,9%	92,7%	91,6%	92,5%	87,7%	88,7%	90,0%	89,3%	84,5%	89,1%	88,9%	88,7%	88,9%	87,5%	86,8%	88,1%	85,4%
B. japonicum 22	92,5%	94,1%	92,5%	92,2%	92,0%	92,7%	93,3%	94,1%	92,9%	91,6%	93,9%	88,7%	88,5%	89,1%	88,3%	86,0%	88,5%	89,7%	89,5%	89,3%	88,1%	88,1%	87,5%	84,1%
B. yuanmingense CCBAU10071	94,1%	94,3%	94,1%	95,2%	92,5%	92,9%	94,1%	96,0%	94,5%	94,1%	93,1%	87,7%	89,5%	90,4%	89,3%	84,7%	89,3%	89,3%	89,1%	91,0%	88,5%	89,1%	90,2%	85,4%
B. yuanmingense CCBAU05623	94,1%	94,3%	94,1%	95,2%	92,5%	92,9%	94,1%	96,0%	94,5%	94,1%	93,1%	87,7%	89,5%	90,4%	89,3%	84,7%	89,3%	89,3%	89,1%	91,0%	88,5%	89,1%	90,2%	85,4%
B. yuanmingense BR3267	94,3%	94,5%	94,3%	95,4%	92,7%	93,1%	94,3%	96,2%	94,7%	94,3%	93,3%	87,9%	89,7%	90,6%	89,5%	84,5%	89,5%	89,5%	89,3%	91,0%	88,7%	88,9%	90,4%	85,2%
B. yuanmingense CCBAU25021	94,1%	94,3%	94,1%	95,2%	92,5%	92,9%	94,1%	96,0%	94,5%	94,1%	93,1%	87,7%	89,5%	90,4%	89,3%	84,7%	89,3%	89,3%	89,1%	91,0%	88,5%	89,1%	90,2%	85,4%
B. yuanmingense CCBAU35157	93,9%	94,1%	93,9%	95,0%	92,2%	92,7%	93,9%	95,8%	94,3%	93,9%	92,9%	87,5%	89,3%	90,2%	89,1%	84,7%	89,1%	89,1%	88,9%	90,8%	88,3%	88,9%	90,0%	85,2%
B. canariense BTA-1	92,5%	94,5%	92,5%	93,7%	93,9%	95,4%	95,6%	94,3%	92,2%	93,3%	95,0%	86,4%	87,9%	87,9%	87,2%	84,5%	87,2%	87,7%	87,9%	88,3%	86,8%	86,8%	87,5%	82,7%
B. canariense UBMA510	92,2%	94,1%	92,2%	92,5%	92,7%	94,5%	95,2%	93,3%	91,4%	92,0%	93,9%	86,2%	86,4%	87,0%	86,6%	84,1%	86,8%	87,5%	87,2%	88,5%	86,6%	86,6%	86,4%	81,6%
B. canariense UBMA183	92,2%	94,1%	92,2%	92,5%	92,7%	94,5%	95,2%	93,3%	91,4%	92,0%	93,9%	86,2%	86,4%	87,0%	86,6%	84,1%	86,8%	87,5%	87,2%	88,5%	86,6%	86,6%	86,4%	81,6%
B. canariense UBMA195	92,2%	94,1%	92,2%	92,5%	92,7%	94,5%	95,2%	93,3%	91,4%	92,0%	93,9%	86,2%	86,4%	87,0%	86,6%	84,1%	86,8%	87,5%	87,2%	88,5%	86,6%	86,6%	86,4%	81,6%
B. canariense UBMAN05	92,2%	94,1%	92,2%	92,5%	92,7%	94,5%	95,2%	93,3%	91,4%	92,0%	93,9%	86,2%	86,4%	87,0%	86,6%	84,1%	86,8%	87,5%	87,2%	88,5%	86,6%	86,6%	86,4%	81,6%
B. liaoningense LMG18230	93,9%	99,7%	93,9%	94,5%	93,9%	94,7%	95,0%	95,6%	93,1%	92,9%	94,5%	88,9%	88,7%	89,5%	88,1%	86,0%	88,7%	90,4%	90,2%	90,4%	88,5%	89,7%	89,1%	83,9%
B. liaoningense CCNWSX0360	92,0%	94,5%	92,0%	92,5%	92,7%	93,3%	93,7%	93,7%	94,1%	92,5%	96,0%	87,0%	87,9%	88,9%	87,7%	84,7%	88,5%	88,7%	88,9%	89,5%	87,5%	88,9%	88,3%	85,4%
B. liaoningense CCBAU83689	93,9%	99,7%	93,9%	94,5%	93,9%	94,7%	95,0%	95,6%	93,1%	92,9%	94,5%	88,9%	88,7%	89,5%	88,1%	86,0%	88,7%	90,4%	90,2%	90,4%	88,5%	89,7%	89,1%	83,9%
B. liaoningense CCBAU05525	93,9%	99,7%	93,9%	94,5%	93,9%	94,7%	95,0%	95,6%	93,1%	92,9%	94,5%	88,9%	88,7%	89,5%	88,1%	86,0%	88,7%	90,4%	90,2%	90,4%	88,5%	89,7%	89,1%	83,9%
B. japonicum USDA4	ID	93,7%	100,0%	94,7%	92,2%	92,5%	93,9%	95,8%	92,7%	94,3%	93,7%	88,7%	89,7%	89,7%	90,2%	85,2%	88,7%	88,9%	89,5%	89,1%	88,7%	89,7%	89,5%	83,9%
B. japonicum USDA135	93,7%	ID	93,7%	94,3%	93,7%	94,5%	94,7%	95,4%	92,9%	92,7%	94,3%	88,7%	88,5%	89,3%	87,9%	85,8%	88,5%	90,2%	90,0%	90,2%	88,3%	89,5%	88,9%	83,7%
B. ottawaense OO99	100,0%	93,7%	ID	94,7%	92,2%	92,5%	93,9%	95,8%	92,7%	94,3%	93,7%	88,7%	89,7%	89,7%	90,2%	85,2%	88,7%	88,9%	89,5%	89,1%	88,7%	89,7%	89,5%	83,9%
B. daqingense CCBAU15774	94,7%	94,3%	94,7%	ID	91,4%	92,7%	93,9%	95,8%	93,9%	94,7%	93,9%	87,2%	89,3%	90,4%	89,3%	84,7%	89,1%	89,1%	89,7%	89,3%	88,5%	89,3%	89,5%	83,7%
B. cytisi CTAW11	92,2%	93,7%	92,2%	91,4%	ID	96,6%	96,2%	92,5%	91,8%	92,7%	93,5%	87,5%	87,5%	88,3%	87,7%	85,4%	87,2%	87,9%	88,1%	88,1%	87,2%	87,5%	87,5%	83,5%
B. rifense CTAW71	92,5%	94,5%	92,5%	92,7%	96,6%	ID	97,7%	93,3%	92,9%	92,9%	95,4%	87,9%	87,5%	88,3%	87,2%	86,0%	87,9%	88,9%	89,5%	89,1%	87,9%	88,1%	87,5%	83,5%
B. ganzhouense RITF806	93,9%	94,7%	93,9%	93,9%	96,2%	97,7%	ID	93,9%	93,3%	93,7%	96,0%	87,7%	88,5%	89,1%	88,3%	86,0%	88,3%	88,5%	88,9%	89,1%	87,7%	88,3%	88,7%	83,1%
B. arachidis CCBAU051107	95,8%	95,4%	95,8%	95,8%	92,5%	93,3%	93,9%	ID	94,3%	95,4%	94,1%	88,9%	89,7%	90,2%	89,3%	84,7%	89,3%	90,0%	89,7%	90,8%	88,9%	89,7%	89,5%	84,3%
B. manausense BR3351	92,7%	92,9%	92,7%	93,9%	91,8%	92,9%	93,3%	94,3%	ID	93,7%	94,3%	87,5%	88,9%	90,2%	88,3%	84,5%	89,5%	90,0%	89,3%	91,0%	88,7%	88,7%	90,4%	86,4%
B. neotropicale BR10247	94,3%	92,7%	94,3%	94,7%	92,7%	92,9%	93,7%	95,4%	93,7%	ID	93,1%	87,5%	88,5%	88,7%	87,9%	84,1%	87,5%	88,5%	88,9%	90,0%	87,7%	88,1%	88,9%	84,1%
B. stylosanthis BR446	93,7%	94,3%	93,7%	93,9%	93,5%	95,4%	96,0%	94,1%	94,3%	93,1%	ID	88,3%	89,1%	90,0%	88,7%	85,8%	89,3%	89,1%	90,2%	88,9%	88,5%	90,0%	89,5%	84,5%
B. pachyrhizi PAC48	88,7%	88,7%	88,7%	87,2%	87,5%	87,9%	87,7%	88,9%	87,5%	87,5%	88,3%	ID	91,2%	89,5%	90,8%	86,8%	89,7%	95,8%	96,6%	92,9%	94,1%	95,0%	90,8%	84,3%
B. paxllaeri LMTR21	89,7%	88,5%	89,7%	89,3%	87,5%	87,5%	88,5%	89,7%	88,9%	88,5%	89,1%	91,2%	ID	95,0%	97,2%	89,3%	93,9%	91,2%	90,8%	89,3%	89,7%	91,0%	96,8%	85,2%
B. icense LMTR13	89,7%	89,3%	89,7%	90,4%	88,3%	88,3%	89,1%	90,2%	90,2%	88,7%	90,0%	89,5%	95,0%	ID	94,7%	88,3%	96,6%	90,4%	90,0%	89,7%	89,3%	90,0%	94,3%	85,4%
B. jicamae PAC68	90,2%	87,9%	90,2%	89,3%	87,7%	87,2%	88,3%	89,3%	88,3%	87,9%	88,7%	90,8%	97,2%	94,7%	ID	89,7%	94,3%	90,6%	90,0%	89,1%	89,5%	90,6%	95,8%	85,2%
B. valentinum LmjM3	85,2%	85,8%	85,2%	84,7%	85,4%	86,0%	86,0%	84,7%	84,5%	84,1%	85,8%	86,8%	89,3%	88,3%	89,7%	ID	88,7%	87,0%	86,0%	85,6%	86,2%	86,6%	88,5%	80,8%
B. retamae Ro19	88,7%	88,5%	88,7%	89,1%	87,2%	87,9%	88,3%	89,3%	89,5%	87,5%	89,3%	89,7%	93,9%	96,6%	94,3%	88,7%	ID	90,4%	89,7%	89,5%	89,5%	90,0%	93,1%	85,0%
B. elkanii USDA76	88,9%	90,2%	88,9%	89,1%	87,9%	88,9%	88,5%	90,0%	90,0%	88,5%	89,1%	95,8%	91,2%	90,4%	90,6%	87,0%	90,4%	ID	97,5%	94,7%	95,4%	95,0%	91,2%	85,6%
B. embrapense SEMIA6208	89,5%	90,0%	89,5%	89,7%	88,1%	89,5%	88,9%	89,7%	89,3%	88,9%	90,2%	96,6%	90,8%	90,0%	90,0%	86,0%	89,7%	97,5%	ID	93,9%	96,2%	95,8%	90,8%	85,6%
B. erythrophlei CCBAU53325	89,1%	90,2%	89,1%	89,3%	88,1%	89,1%	89,1%	90,8%	91,0%	90,0%	88,9%	92,9%	89,3%	89,7%	89,1%	85,6%	89,5%	94,7%	93,9%	ID	93,7%	93,7%	90,0%	86,0%
B. viridifuturi SEMIA690	88,7%	88,3%	88,7%	88,5%	87,2%	87,9%	87,7%	88,9%	88,7%	87,7%	88,5%	94,1%	89,7%	89,3%	89,5%	86,2%	89,5%	95,4%	96,2%	93,7%	ID	94,1%	90,2%	84,7%
B. mercantei SEMIA6399	89,7%	89,5%	89,7%	89,3%	87,5%	88,1%	88,3%	89,7%	88,7%	88,1%	90,0%	95,0%	91,0%	90,0%	90,6%	86,6%	90,0%	95,0%	95,8%	93,7%	94,1%	ID	91,8%	84,7%
B. lablabi CCBAU23086	89,5%	88,9%	89,5%	89,5%	87,5%	87,5%	88,7%	89,5%	90,4%	88,9%	89,5%	90,8%	96,8%	94,3%	95,8%	88,5%	93,1%	91,2%	90,8%	90,0%	90,2%	91,8%	ID	84,1%
Rhodopseudomonas plaustris CGA009	83,9%	83,7%	83,9%	83,7%	83,5%	83,5%	83,1%	84,3%	86,4%	84,1%	84,5%	84,3%	85,2%	85,4%	85,2%	80,8%	85,0%	85,6%	85,6%	86,0%	84,7%	84,7%	84,1%	ID

Supplementary Table S4. *recA* gene sequence similarity between strains used in the study

	Bradyrhizobium sp. JAN6	*Bradyrhizobium* sp. JAN10	*Bradyrhizobium* sp. GER1	*Bradyrhizobium* sp. GER32	*Bradyrhizobium* sp. ZAR4	*Bradyrhizobium* sp. ZAR12	*Bradyrhizobium* sp. SZCZ11	*Bradyrhizobium* sp. SZCZ20	*B. japonicum* USDA6	*B. japonicum* E109	*B. japonicum* J5	*B. japonicum* SEMIA5079	*B. japonicum* USDA38	*B. japonicum* USDA123	*B. japonicum* CCBAU15354	*B. japonicum* CCBAU15517	*B. japonicum* CCBAU15618	*B. japonicum* CCBAU25435	*B. japonicum* UBMA197	*B. japonicum* Is-34	*B. japonicum* FN1	*B. diazoefficiens* USDA110	*B. diazoefficiens* NK6	*B. diazoefficiens* USDA122	*B. diazoefficiens* SEMIA5080	*B. japonicum* USDA124	*B. japonicum* Is-1	*B. huanghuaihaiense* CCBAU23303	*B. japonicum* in8p8	*B. japonicum* is5	*B. japonicum* 22	*B. yuanmingense* CCBAU10071	*B. yuanmingense* BR3267	*B. yuanmingense* CCBAU05623	*B. yuanmingense* CCBAU25021	*B. yuanmingense* CCBAU35157	*B. canariense* BTA-1	*B. canariense* UBMA050	*B. canariense* UBMA195	*B. canariense* UBMAN05	*B. canariense* UBMA183	*B. liaoningense* LMG18230	*B. liaoningense* CCBAU05525	*B. liaoningense* CCBAU83689	*B. liaoningense* CCNWSX0360	*B. japonicum* USDA4	*B. japonicum* USDA135	*B. ottawaense* OO99	*B. daqingense* CCBAU15774	*B. cytisi* CTAW11	*B. rifense* CTAW71	*B. arachidis* CCBAU051107	*B. ganzhouense* RITF806	*B. manausense* BR3351	*B. stylosanthis* BR446	*B. neotropicale* BR10247	*B. embrapense* SEMIA6208	*B. valentinum* LmjM3	*B. elkanii* USDA76	*B. erythrophlei* CCBAU53325	*B. retamae* Ro19	*B. viridifuturi* SEMIA690	*B. lablabi* CCBAU23086	*B. pachyrhizi* LMG24246	*B. jicamae* LMG24556	*B. icense* LMTR13	*B. paxllaeri* LMTR21	*B. mercantei* SEMIA6399	*Rhodopseudomonas palustris* CGA009
Bradyrhizobium sp. JAN6	ID	98,9%	98,9%	99,2%	98,9%	98,9%	98,9%	98,9%	97,6%	97,6%	97,3%	98,4%	98,4%	97,3%	97,3%	97,3%	98,1%	98,1%	98,9%	98,4%	97,6%	94,6%	96,2%	96,5%	96,5%	90,1%	96,5%	90,1%	91,4%	91,4%	92,2%	92,0%	91,7%	90,4%	92,0%	90,9%	93,8%	93,3%	93,3%	93,3%	93,3%	93,0%	93,0%	93,0%	92,2%	93,0%	93,0%	93,0%	91,7%	92,5%	91,7%	93,3%	92,5%	91,2%	93,6%	92,8%	92,2%	90,9%	92,5%	90,4%	91,7%	91,2%	90,9%	91,4%	88,5%	90,6%	89,0%	92,2%	84,8%
Bradyrhizobium sp. JAN10	98,9%	ID	100,0%	99,7%	100,0%	100,0%	100,0%	100,0%	98,1%	98,1%	97,8%	98,9%	98,9%	97,8%	97,8%	97,8%	98,6%	98,6%	100,0%	98,9%	98,1%	95,2%	96,2%	96,5%	96,5%	90,4%	96,5%	90,4%	90,9%	90,9%	91,7%	91,4%	91,2%	89,8%	91,4%	90,4%	94,6%	94,1%	94,1%	94,1%	94,1%	93,3%	93,3%	93,3%	92,5%	92,5%	93,3%	92,5%	91,2%	93,0%	92,2%	93,3%	93,0%	91,2%	93,6%	92,8%	92,0%	91,2%	92,2%	90,6%	91,7%	91,4%	91,2%	91,2%	88,8%	90,9%	89,3%	92,5%	84,5%
Bradyrhizobium sp. GER1	98,9%	100,0%	ID	99,7%	100,0%	100,0%	100,0%	100,0%	98,1%	98,1%	97,8%	98,9%	98,9%	97,8%	97,8%	97,8%	98,6%	98,6%	100,0%	98,9%	98,1%	95,2%	96,2%	96,5%	96,5%	90,4%	96,5%	90,4%	90,9%	90,9%	91,7%	91,4%	91,2%	89,8%	91,4%	90,4%	94,6%	94,1%	94,1%	94,1%	94,1%	93,3%	93,3%	93,3%	92,5%	92,5%	93,3%	92,5%	91,2%	93,0%	92,2%	93,3%	93,0%	91,2%	93,6%	92,8%	92,0%	91,2%	92,2%	90,6%	91,7%	91,4%	91,2%	91,2%	88,8%	90,9%	89,3%	92,5%	84,5%
Bradyrhizobium sp. GER32	99,2%	99,7%	99,7%	ID	99,7%	99,7%	99,7%	99,7%	98,4%	98,4%	98,1%	99,2%	99,2%	98,1%	98,1%	98,1%	98,9%	98,9%	99,7%	99,2%	98,4%	95,4%	96,5%	96,8%	96,8%	90,6%	96,8%	90,6%	90,9%	90,9%	92,0%	91,4%	91,2%	89,8%	91,4%	90,4%	94,6%	94,1%	94,1%	94,1%	94,1%	93,3%	93,3%	93,3%	92,5%	92,8%	93,3%	92,8%	91,4%	93,3%	92,5%	93,6%	93,3%	91,4%	93,8%	93,0%	92,0%	91,2%	92,2%	90,6%	91,7%	91,4%	91,2%	91,2%	88,8%	90,9%	89,3%	92,5%	84,5%
Bradyrhizobium sp. ZAR4	98,9%	100,0%	100,0%	99,7%	ID	100,0%	100,0%	100,0%	98,1%	98,1%	97,8%	98,9%	98,9%	97,8%	97,8%	97,8%	98,6%	98,6%	100,0%	98,9%	98,1%	95,2%	96,2%	96,5%	96,5%	90,4%	96,5%	90,4%	90,9%	90,9%	91,7%	91,4%	91,2%	89,8%	91,4%	90,4%	94,6%	94,1%	94,1%	94,1%	94,1%	93,3%	93,3%	93,3%	92,5%	92,5%	93,3%	92,5%	91,2%	93,0%	92,2%	93,3%	93,0%	91,2%	93,6%	92,8%	92,0%	91,2%	92,2%	90,6%	91,7%	91,4%	91,2%	91,2%	88,8%	90,9%	89,3%	92,5%	84,5%
Bradyrhizobium sp. ZAR12	98,9%	100,0%	100,0%	99,7%	100,0%	ID	100,0%	100,0%	98,1%	98,1%	97,8%	98,9%	98,9%	97,8%	97,8%	97,8%	98,6%	98,6%	100,0%	98,9%	98,1%	95,2%	96,2%	96,5%	96,5%	90,4%	96,5%	90,4%	90,9%	90,9%	91,7%	91,4%	91,2%	89,8%	91,4%	90,4%	94,6%	94,1%	94,1%	94,1%	94,1%	93,3%	93,3%	93,3%	92,5%	92,5%	93,3%	92,5%	91,2%	93,0%	92,2%	93,3%	93,0%	91,2%	93,6%	92,8%	92,0%	91,2%	92,2%	90,6%	91,7%	91,4%	91,2%	91,2%	88,8%	90,9%	89,3%	92,5%	84,5%
Bradyrhizobium sp. SZCZ11	98,9%	100,0%	100,0%	99,7%	100,0%	100,0%	ID	100,0%	98,1%	98,1%	97,8%	98,9%	98,9%	97,8%	97,8%	97,8%	98,6%	98,6%	100,0%	98,9%	98,1%	95,2%	96,2%	96,5%	96,5%	90,4%	96,5%	90,4%	90,9%	90,9%	91,7%	91,4%	91,2%	89,8%	91,4%	90,4%	94,6%	94,1%	94,1%	94,1%	94,1%	93,3%	93,3%	93,3%	92,5%	92,5%	93,3%	92,5%	91,2%	93,0%	92,2%	93,3%	93,0%	91,2%	93,6%	92,8%	92,0%	91,2%	92,2%	90,6%	91,7%	91,4%	91,2%	91,2%	88,8%	90,9%	89,3%	92,5%	84,5%
Bradyrhizobium sp. SZCZ20	98,9%	100,0%	100,0%	99,7%	100,0%	100,0%	100,0%	ID	98,1%	98,1%	97,8%	98,9%	98,9%	97,8%	97,8%	97,8%	98,6%	98,6%	100,0%	98,9%	98,1%	95,2%	96,2%	96,5%	96,5%	90,4%	96,5%	90,4%	90,9%	90,9%	91,7%	91,4%	91,2%	89,8%	91,4%	90,4%	94,6%	94,1%	94,1%	94,1%	94,1%	93,3%	93,3%	93,3%	92,5%	92,5%	93,3%	92,5%	91,2%	93,0%	92,2%	93,3%	93,0%	91,2%	93,6%	92,8%	92,0%	91,2%	92,2%	90,6%	91,7%	91,4%	91,2%	91,2%	88,8%	90,9%	89,3%	92,5%	84,5%
B. japonicum USDA6	97,6%	98,1%	98,1%	98,4%	98,1%	98,1%	98,1%	98,1%	ID	100,0%	97,0%	99,2%	98,6%	97,0%	97,0%	97,0%	99,4%	99,4%	98,1%	98,6%	100,0%	94,9%	96,0%	96,2%	96,2%	91,2%	96,2%	91,2%	90,4%	90,4%	92,0%	92,0%	91,2%	89,8%	92,0%	90,9%	94,4%	94,6%	94,6%	94,6%	94,6%	93,3%	93,3%	93,3%	93,0%	92,8%	93,3%	92,8%	90,6%	92,2%	92,5%	93,6%	92,8%	91,4%	93,8%	92,5%	91,4%	90,9%	92,2%	90,1%	90,9%	92,0%	91,2%	91,4%	89,0%	90,4%	89,3%	92,5%	84,0%
B. japonicum E109	97,6%	98,1%	98,1%	98,4%	98,1%	98,1%	98,1%	98,1%	100,0%	ID	97,0%	99,2%	98,6%	97,0%	97,0%	97,0%	99,4%	99,4%	98,1%	98,6%	100,0%	94,9%	96,0%	96,2%	96,2%	91,2%	96,2%	91,2%	90,4%	90,4%	92,0%	92,0%	91,2%	89,8%	92,0%	90,9%	94,4%	94,6%	94,6%	94,6%	94,6%	93,3%	93,3%	93,3%	93,0%	92,8%	93,3%	92,8%	90,6%	92,2%	92,5%	93,6%	92,8%	91,4%	93,8%	92,5%	91,4%	90,9%	92,2%	90,1%	90,9%	92,0%	91,2%	91,4%	89,0%	90,4%	89,3%	92,5%	84,0%
B. japonicum J5	97,3%	97,8%	97,8%	98,1%	97,8%	97,8%	97,8%	97,8%	97,0%	97,0%	ID	97,8%	97,8%	97,3%	97,3%	97,3%	97,6%	97,6%	97,8%	97,8%	97,0%	95,7%	96,8%	97,0%	97,0%	90,4%	97,0%	90,4%	90,1%	90,1%	91,2%	90,6%	90,4%	89,0%	90,6%	89,6%	94,9%	93,3%	93,3%	93,3%	93,3%	92,5%	92,5%	92,5%	91,7%	92,0%	92,5%	92,0%	90,1%	93,0%	91,7%	92,8%	92,5%	90,6%	93,6%	91,7%	91,7%	91,4%	92,0%	89,3%	91,4%	91,2%	91,4%	90,4%	89,0%	91,7%	89,6%	91,2%	85,6%
B. japonicum SEMIA5079	98,4%	98,9%	98,9%	99,2%	98,9%	98,9%	98,9%	98,9%	99,2%	99,2%	97,8%	ID	99,4%	97,8%	97,8%	97,8%	99,2%	99,2%	98,9%	99,4%	99,2%	95,7%	96,8%	97,0%	97,0%	90,9%	97,0%	90,9%	90,6%	90,6%	92,2%	92,2%	91,4%	90,1%	92,2%	91,2%	94,6%	94,4%	94,4%	94,4%	94,4%	93,6%	93,6%	93,6%	93,3%	93,0%	93,6%	93,0%	90,9%	93,0%	92,8%	93,8%	93,6%	91,7%	94,1%	92,8%	92,2%	91,4%	92,5%	90,4%	91,4%	92,2%	91,2%	91,7%	89,0%	90,9%	89,3%	92,8%	84,2%
B. japonicum USDA38	98,4%	98,9%	98,9%	99,2%	98,9%	98,9%	98,9%	98,9%	98,6%	98,6%	97,8%	99,4%	ID	97,8%	97,8%	97,8%	99,2%	99,2%	98,9%	100,0%	98,6%	95,2%	96,2%	96,5%	96,5%	90,9%	96,5%	90,9%	90,6%	90,6%	92,2%	92,2%	91,4%	90,1%	92,2%	91,2%	94,4%	93,8%	93,8%	93,8%	93,8%	93,6%	93,6%	93,6%	93,3%	93,0%	93,6%	93,0%	91,2%	92,5%	92,2%	93,3%	93,0%	91,2%	93,6%	92,8%	91,7%	90,9%	92,0%	90,4%	91,4%	91,7%	90,9%	91,2%	89,0%	90,6%	89,6%	92,2%	83,7%
B. japonicum USDA123	97,3%	97,8%	97,8%	98,1%	97,8%	97,8%	97,8%	97,8%	97,0%	97,0%	97,3%	97,8%	97,8%	ID	100,0%	100,0%	97,6%	97,6%	97,8%	97,8%	97,0%	94,9%	96,0%	96,2%	96,2%	90,1%	96,2%	90,1%	89,3%	89,3%	91,2%	90,6%	90,4%	89,0%	90,6%	90,1%	93,8%	93,3%	93,3%	93,3%	93,3%	92,5%	92,5%	92,5%	91,7%	91,7%	92,5%	91,7%	90,1%	92,2%	91,7%	93,3%	92,2%	90,6%	93,0%	92,0%	91,4%	90,4%	92,0%	89,3%	91,4%	90,4%	90,4%	90,4%	88,0%	90,6%	88,5%	91,2%	84,0%
B. japonicum CCBAU15354	97,3%	97,8%	97,8%	98,1%	97,8%	97,8%	97,8%	97,8%	97,0%	97,0%	97,3%	97,8%	97,8%	100,0%	ID	100,0%	97,6%	97,6%	97,8%	97,8%	97,0%	94,9%	96,0%	96,2%	96,2%	90,1%	96,2%	90,1%	89,3%	89,3%	91,2%	90,6%	90,4%	89,0%	90,6%	90,1%	93,8%	93,3%	93,3%	93,3%	93,3%	92,5%	92,5%	92,5%	91,7%	91,7%	92,5%	91,7%	90,1%	92,2%	91,7%	93,3%	92,2%	90,6%	93,0%	92,0%	91,4%	90,4%	92,0%	89,3%	91,4%	90,4%	90,4%	90,4%	88,0%	90,6%	88,5%	91,2%	84,0%
B. japonicum CCBAU15517	97,3%	97,8%	97,8%	98,1%	97,8%	97,8%	97,8%	97,8%	97,0%	97,0%	97,3%	97,8%	97,8%	100,0%	100,0%	ID	97,6%	97,6%	97,8%	97,8%	97,0%	94,9%	96,0%	96,2%	96,2%	90,1%	96,2%	90,1%	89,3%	89,3%	91,2%	90,6%	90,4%	89,0%	90,6%	90,1%	93,8%	93,3%	93,3%	93,3%	93,3%	92,5%	92,5%	92,5%	91,7%	91,7%	92,5%	91,7%	90,1%	92,2%	91,7%	93,3%	92,2%	90,6%	93,0%	92,0%	91,4%	90,4%	92,0%	89,3%	91,4%	90,4%	90,4%	90,4%	88,0%	90,6%	88,5%	91,2%	84,0%
B. japonicum CCBAU15618	98,1%	98,6%	98,6%	98,9%	98,6%	98,6%	98,6%	98,6%	99,4%	99,4%	97,6%	99,2%	99,2%	97,6%	97,6%	97,6%	ID	100,0%	98,6%	99,2%	99,4%	94,9%	96,0%	96,2%	96,2%	90,6%	96,2%	90,6%	90,4%	90,4%	92,0%	92,0%	91,2%	89,8%	92,0%	90,9%	94,6%	94,6%	94,6%	94,6%	94,6%	93,3%	93,3%	93,3%	93,0%	92,8%	93,3%	92,8%	90,9%	92,2%	92,5%	93,6%	92,8%	91,4%	93,8%	92,5%	91,4%	90,9%	92,2%	90,1%	91,4%	92,0%	91,4%	91,4%	89,0%	90,6%	89,6%	92,5%	84,0%
B. japonicum CCBAU25435	98,1%	98,6%	98,6%	98,9%	98,6%	98,6%	98,6%	98,6%	99,4%	99,4%	97,6%	99,2%	99,2%	97,6%	97,6%	97,6%	100,0%	ID	98,6%	99,2%	99,4%	94,9%	96,0%	96,2%	96,2%	90,6%	96,2%	90,6%	90,4%	90,4%	92,0%	92,0%	91,2%	89,8%	92,0%	90,9%	94,6%	94,6%	94,6%	94,6%	94,6%	93,3%	93,3%	93,3%	93,0%	92,8%	93,3%	92,8%	90,9%	92,2%	92,5%	93,6%	92,8%	91,4%	93,8%	92,5%	91,4%	90,9%	92,2%	90,1%	91,4%	92,0%	91,4%	91,4%	89,0%	90,6%	89,6%	92,5%	84,0%
B. japonicum UBMA197	98,9%	100,0%	100,0%	99,7%	100,0%	100,0%	100,0%	100,0%	98,1%	98,1%	97,8%	98,9%	98,9%	97,8%	97,8%	97,8%	98,6%	98,6%	ID	98,9%	98,1%	95,2%	96,2%	96,5%	96,5%	90,4%	96,5%	90,4%	90,9%	90,9%	91,7%	91,4%	91,2%	89,8%	91,4%	90,4%	94,6%	94,1%	94,1%	94,1%	94,1%	93,3%	93,3%	93,3%	92,5%	92,5%	93,3%	92,5%	91,2%	93,0%	92,2%	93,3%	93,0%	91,2%	93,6%	92,8%	92,0%	91,2%	92,2%	90,6%	91,7%	91,4%	91,2%	91,2%	88,8%	90,9%	89,3%	92,5%	84,5%
B. japonicum Is-34	98,4%	98,9%	98,9%	99,2%	98,9%	98,9%	98,9%	98,9%	98,6%	98,6%	97,8%	99,4%	100,0%	97,8%	97,8%	97,8%	99,2%	99,2%	98,9%	ID	98,6%	95,2%	96,2%	96,5%	96,5%	90,9%	96,5%	90,9%	90,6%	90,6%	92,2%	92,2%	91,4%	90,1%	92,2%	91,2%	94,4%	93,8%	93,8%	93,8%	93,8%	93,6%	93,6%	93,6%	93,3%	93,0%	93,6%	93,0%	91,2%	92,5%	92,2%	93,3%	93,0%	91,2%	93,6%	92,8%	91,7%	90,9%	92,0%	90,4%	91,4%	91,7%	90,9%	91,2%	89,0%	90,6%	89,6%	92,2%	83,7%
B. japonicum FN1	97,6%	98,1%	98,1%	98,4%	98,1%	98,1%	98,1%	98,1%	100,0%	100,0%	97,0%	99,2%	98,6%	97,0%	97,0%	97,0%	99,4%	99,4%	98,1%	98,6%	ID	94,9%	96,0%	96,2%	96,2%	91,2%	96,2%	91,2%	90,4%	90,4%	92,0%	92,0%	91,2%	89,8%	92,0%	90,9%	94,4%	94,6%	94,6%	94,6%	94,6%	93,3%	93,3%	93,3%	93,0%	92,8%	93,3%	92,8%	90,6%	92,2%	92,5%	93,6%	92,8%	91,4%	93,8%	92,5%	91,4%	90,9%	92,2%	90,1%	90,9%	92,0%	91,2%	91,4%	89,0%	90,4%	89,3%	92,5%	84,0%
B. diazoefficiens USDA110	94,6%	95,2%	95,2%	95,4%	95,2%	95,2%	95,2%	95,2%	94,9%	94,9%	95,7%	95,7%	95,2%	94,9%	94,9%	94,9%	94,9%	94,9%	95,2%	95,2%	94,9%	ID	97,8%	97,6%	97,6%	90,4%	97,6%	90,4%	90,9%	90,9%	92,0%	91,4%	91,2%	90,1%	91,4%	90,6%	93,0%	92,8%	92,8%	92,8%	92,8%	93,3%	93,3%	93,3%	92,5%	91,4%	93,3%	91,4%	90,6%	92,0%	91,4%	92,0%	93,6%	91,7%	93,3%	92,0%	91,7%	90,9%	92,0%	89,8%	90,6%	91,2%	90,4%	90,4%	88,8%	90,9%	88,8%	91,2%	85,3%
B. diazoefficiens NK6	96,2%	96,2%	96,2%	96,5%	96,2%	96,2%	96,2%	96,2%	96,0%	96,0%	96,8%	96,8%	96,2%	96,0%	96,0%	96,0%	96,0%	96,0%	96,2%	96,2%	96,0%	97,8%	ID	99,7%	99,7%	91,4%	99,7%	91,4%	92,2%	92,2%	92,8%	92,2%	92,0%	90,9%	92,2%	91,4%	94,6%	94,4%	94,4%	94,4%	94,4%	94,1%	94,1%	94,1%	93,3%	93,6%	94,1%	93,6%	91,4%	93,0%	93,0%	93,3%	94,4%	92,0%	94,1%	92,8%	92,8%	92,2%	93,0%	91,4%	91,7%	92,8%	92,0%	92,0%	89,6%	92,0%	89,8%	92,8%	86,1%
B. diazoefficiens USDA122	96,5%	96,5%	96,5%	96,8%	96,5%	96,5%	96,5%	96,5%	96,2%	96,2%	97,0%	97,0%	96,5%	96,2%	96,2%	96,2%	96,2%	96,2%	96,5%	96,5%	96,2%	97,6%	99,7%	ID	100,0%	91,7%	100,0%	91,7%	92,5%	92,5%	93,0%	92,5%	92,2%	91,2%	92,5%	91,7%	94,4%	94,1%	94,1%	94,1%	94,1%	94,4%	94,4%	94,4%	93,6%	93,8%	94,4%	93,8%	91,7%	92,8%	92,8%	93,6%	94,1%	92,2%	94,4%	93,0%	93,0%	92,5%	93,3%	91,2%	92,0%	93,0%	92,2%	92,2%	89,8%	92,2%	90,1%	93,0%	85,8%
B. diazoefficiens SEMIA5080	96,5%	96,5%	96,5%	96,8%	96,5%	96,5%	96,5%	96,5%	96,2%	96,2%	97,0%	97,0%	96,5%	96,2%	96,2%	96,2%	96,2%	96,2%	96,5%	96,5%	96,2%	97,6%	99,7%	100,0%	ID	91,7%	100,0%	91,7%	92,5%	92,5%	93,0%	92,5%	92,2%	91,2%	92,5%	91,7%	94,4%	94,1%	94,1%	94,1%	94,1%	94,4%	94,4%	94,4%	93,6%	93,8%	94,4%	93,8%	91,7%	92,8%	92,8%	93,6%	94,1%	92,2%	94,4%	93,0%	93,0%	92,5%	93,3%	91,2%	92,0%	93,0%	92,2%	92,2%	89,8%	92,2%	90,1%	93,0%	85,8%
B. japonicum USDA124	90,1%	90,4%	90,4%	90,6%	90,4%	90,4%	90,4%	90,4%	91,2%	91,2%	90,4%	90,9%	90,9%	90,1%	90,1%	90,1%	90,6%	90,6%	90,4%	90,9%	91,2%	90,4%	91,4%	91,7%	91,7%	ID	91,7%	100,0%	91,4%	91,4%	92,5%	93,3%	93,8%	94,6%	93,3%	94,9%	90,6%	91,4%	91,4%	91,4%	91,4%	94,4%	94,4%	94,4%	93,0%	91,7%	94,4%	91,7%	94,6%	92,0%	92,5%	93,8%	89,8%	94,1%	94,4%	93,6%	90,4%	90,4%	90,1%	91,7%	90,4%	91,2%	91,4%	90,4%	89,3%	89,8%	89,3%	91,7%	86,1%
B. japonicum Is-1	96,5%	96,5%	96,5%	96,8%	96,5%	96,5%	96,5%	96,5%	96,2%	96,2%	97,0%	97,0%	96,5%	96,2%	96,2%	96,2%	96,2%	96,2%	96,5%	96,5%	96,2%	97,6%	99,7%	100,0%	100,0%	91,7%	ID	91,7%	92,5%	92,5%	93,0%	92,5%	92,2%	91,2%	92,5%	91,7%	94,4%	94,1%	94,1%	94,1%	94,1%	94,4%	94,4%	94,4%	93,6%	93,8%	94,4%	93,8%	91,7%	92,8%	92,8%	93,6%	94,1%	92,2%	94,4%	93,0%	93,0%	92,5%	93,3%	91,2%	92,0%	93,0%	92,2%	92,2%	89,8%	92,2%	90,1%	93,0%	85,8%
B. huanghuaihaiense CCBAU23303	90,1%	90,4%	90,4%	90,6%	90,4%	90,4%	90,4%	90,4%	91,2%	91,2%	90,4%	90,9%	90,9%	90,1%	90,1%	90,1%	90,6%	90,6%	90,4%	90,9%	91,2%	90,4%	91,4%	91,7%	91,7%	100,0%	91,7%	ID	91,4%	91,4%	92,5%	93,3%	93,8%	94,6%	93,3%	94,9%	90,6%	91,4%	91,4%	91,4%	91,4%	94,4%	94,4%	94,4%	93,0%	91,7%	94,4%	91,7%	94,6%	92,0%	92,5%	93,8%	89,8%	94,1%	94,4%	93,6%	90,4%	90,4%	90,1%	91,7%	90,4%	91,2%	91,4%	90,4%	89,3%	89,8%	89,3%	91,7%	86,1%
B. japonicum in8p8	91,4%	90,9%	90,9%	90,9%	90,9%	90,9%	90,9%	90,9%	90,4%	90,4%	90,1%	90,6%	90,6%	89,3%	89,3%	89,3%	90,4%	90,4%	90,9%	90,6%	90,4%	90,9%	92,2%	92,5%	92,5%	91,4%	92,5%	91,4%	ID	100,0%	93,6%	94,1%	94,4%	93,3%	94,1%	93,8%	90,6%	92,0%	92,0%	92,0%	92,0%	93,8%	93,8%	93,8%	93,0%	92,2%	93,8%	92,2%	92,5%	90,6%	91,2%	92,0%	92,0%	90,9%	92,8%	92,8%	92,0%	89,3%	92,2%	90,6%	89,0%	90,9%	90,4%	91,2%	88,0%	89,3%	88,2%	91,4%	85,8%
B. japonicum is5	91,4%	90,9%	90,9%	90,9%	90,9%	90,9%	90,9%	90,9%	90,4%	90,4%	90,1%	90,6%	90,6%	89,3%	89,3%	89,3%	90,4%	90,4%	90,9%	90,6%	90,4%	90,9%	92,2%	92,5%	92,5%	91,4%	92,5%	91,4%	100,0%	ID	93,6%	94,1%	94,4%	93,3%	94,1%	93,8%	90,6%	92,0%	92,0%	92,0%	92,0%	93,8%	93,8%	93,8%	93,0%	92,2%	93,8%	92,2%	92,5%	90,6%	91,2%	92,0%	92,0%	90,9%	92,8%	92,8%	92,0%	89,3%	92,2%	90,6%	89,0%	90,9%	90,4%	91,2%	88,0%	89,3%	88,2%	91,4%	85,8%
B. japonicum 22	92,2%	91,7%	91,7%	92,0%	91,7%	91,7%	91,7%	91,7%	92,0%	92,0%	91,2%	92,2%	92,2%	91,2%	91,2%	91,2%	92,0%	92,0%	91,7%	92,2%	92,0%	92,0%	92,8%	93,0%	93,0%	92,5%	93,0%	92,5%	93,6%	93,6%	ID	94,6%	94,6%	92,8%	94,6%	93,3%	92,2%	93,0%	93,0%	93,0%	93,0%	96,0%	96,0%	96,0%	95,7%	96,2%	96,0%	96,2%	93,3%	90,9%	92,2%	94,1%	93,3%	94,4%	95,4%	94,6%	92,5%	92,0%	92,8%	93,3%	89,6%	93,0%	91,2%	93,3%	89,0%	90,1%	89,3%	93,0%	86,4%
B. yuanmingense CCBAU10071	92,0%	91,4%	91,4%	91,4%	91,4%	91,4%	91,4%	91,4%	92,0%	92,0%	90,6%	92,2%	92,2%	90,6%	90,6%	90,6%	92,0%	92,0%	91,4%	92,2%	92,0%	91,4%	92,2%	92,5%	92,5%	93,3%	92,5%	93,3%	94,1%	94,1%	94,6%	ID	98,1%	96,2%	100,0%	96,8%	91,7%	92,5%	92,5%	92,5%	92,5%	96,0%	96,0%	96,0%	95,2%	93,3%	96,0%	93,3%	93,3%	90,4%	91,7%	94,4%	91,4%	92,8%	95,4%	94,4%	91,4%	90,9%	92,2%	92,8%	91,2%	93,0%	92,0%	93,0%	89,6%	90,4%	90,4%	93,8%	86,4%
B. yuanmingense BR3267	91,7%	91,2%	91,2%	91,2%	91,2%	91,2%	91,2%	91,2%	91,2%	91,2%	90,4%	91,4%	91,4%	90,4%	90,4%	90,4%	91,2%	91,2%	91,2%	91,4%	91,2%	91,2%	92,0%	92,2%	92,2%	93,8%	92,2%	93,8%	94,4%	94,4%	94,6%	98,1%	ID	97,0%	98,1%	96,0%	91,4%	92,2%	92,2%	92,2%	92,2%	95,7%	95,7%	95,7%	94,4%	93,3%	95,7%	93,3%	93,3%	90,1%	92,0%	94,1%	91,4%	92,8%	95,2%	94,1%	91,7%	90,9%	92,5%	92,8%	90,9%	92,2%	92,0%	93,0%	90,4%	90,6%	90,6%	93,0%	86,6%
B. yuanmingense CCBAU05623	90,4%	89,8%	89,8%	89,8%	89,8%	89,8%	89,8%	89,8%	89,8%	89,8%	89,0%	90,1%	90,1%	89,0%	89,0%	89,0%	89,8%	89,8%	89,8%	90,1%	89,8%	90,1%	90,9%	91,2%	91,2%	94,6%	91,2%	94,6%	93,3%	93,3%	92,8%	96,2%	97,0%	ID	96,2%	97,6%	90,1%	90,9%	90,9%	90,9%	90,9%	94,4%	94,4%	94,4%	93,0%	91,7%	94,4%	91,7%	93,0%	91,2%	91,2%	92,8%	89,6%	93,3%	93,6%	92,8%	89,8%	89,6%	91,2%	92,0%	89,6%	91,4%	90,6%	91,7%	88,8%	89,3%	89,0%	91,7%	85,6%
B. yuanmingense CCBAU25021	92,0%	91,4%	91,4%	91,4%	91,4%	91,4%	91,4%	91,4%	92,0%	92,0%	90,6%	92,2%	92,2%	90,6%	90,6%	90,6%	92,0%	92,0%	91,4%	92,2%	92,0%	91,4%	92,2%	92,5%	92,5%	93,3%	92,5%	93,3%	94,1%	94,1%	94,6%	100,0%	98,1%	96,2%	ID	96,8%	91,7%	92,5%	92,5%	92,5%	92,5%	96,0%	96,0%	96,0%	95,2%	93,3%	96,0%	93,3%	93,3%	90,4%	91,7%	94,4%	91,4%	92,8%	95,4%	94,4%	91,4%	90,9%	92,2%	92,8%	91,2%	93,0%	92,0%	93,0%	89,6%	90,4%	90,4%	93,8%	86,4%
B. yuanmingense CCBAU35157	90,9%	90,4%	90,4%	90,4%	90,4%	90,4%	90,4%	90,4%	90,9%	90,9%	89,6%	91,2%	91,2%	90,1%	90,1%	90,1%	90,9%	90,9%	90,4%	91,2%	90,9%	90,6%	91,4%	91,7%	91,7%	94,9%	91,7%	94,9%	93,8%	93,8%	93,3%	96,8%	96,0%	97,6%	96,8%	ID	90,9%	91,7%	91,7%	91,7%	91,7%	94,4%	94,4%	94,4%	94,1%	92,2%	94,4%	92,2%	93,8%	91,4%	91,4%	94,1%	90,1%	94,6%	94,4%	93,3%	91,2%	90,4%	91,4%	92,8%	90,4%	92,2%	91,4%	92,0%	89,0%	90,1%	89,8%	93,0%	86,9%
B. canariense BTA-1	93,8%	94,6%	94,6%	94,6%	94,6%	94,6%	94,6%	94,6%	94,4%	94,4%	94,9%	94,6%	94,4%	93,8%	93,8%	93,8%	94,6%	94,6%	94,6%	94,4%	94,4%	93,0%	94,6%	94,4%	94,4%	90,6%	94,4%	90,6%	90,6%	90,6%	92,2%	91,7%	91,4%	90,1%	91,7%	90,9%	ID	97,6%	97,6%	97,6%	97,6%	94,1%	94,1%	94,1%	93,3%	92,5%	94,1%	92,5%	90,6%	94,4%	94,9%	93,0%	92,8%	92,0%	93,6%	92,2%	92,8%	92,8%	93,6%	92,2%	92,5%	93,3%	93,3%	93,6%	90,4%	92,8%	91,2%	93,8%	86,6%
B. canariense UBMA050	93,3%	94,1%	94,1%	94,1%	94,1%	94,1%	94,1%	94,1%	94,6%	94,6%	93,3%	94,4%	93,8%	93,3%	93,3%	93,3%	94,6%	94,6%	94,1%	93,8%	94,6%	92,8%	94,4%	94,1%	94,1%	91,4%	94,1%	91,4%	92,0%	92,0%	93,0%	92,5%	92,2%	90,9%	92,5%	91,7%	97,6%	ID	100,0%	100,0%	100,0%	94,4%	94,4%	94,4%	93,6%	93,3%	94,4%	93,3%	90,9%	94,1%	96,0%	93,8%	94,1%	92,8%	94,4%	93,0%	92,5%	92,8%	93,8%	92,5%	92,2%	93,0%	92,2%	93,8%	89,8%	91,7%	90,1%	94,6%	85,6%
B. canariense UBMA195	93,3%	94,1%	94,1%	94,1%	94,1%	94,1%	94,1%	94,1%	94,6%	94,6%	93,3%	94,4%	93,8%	93,3%	93,3%	93,3%	94,6%	94,6%	94,1%	93,8%	94,6%	92,8%	94,4%	94,1%	94,1%	91,4%	94,1%	91,4%	92,0%	92,0%	93,0%	92,5%	92,2%	90,9%	92,5%	91,7%	97,6%	100,0%	ID	100,0%	100,0%	94,4%	94,4%	94,4%	93,6%	93,3%	94,4%	93,3%	90,9%	94,1%	96,0%	93,8%	94,1%	92,8%	94,4%	93,0%	92,5%	92,8%	93,8%	92,5%	92,2%	93,0%	92,2%	93,8%	89,8%	91,7%	90,1%	94,6%	85,6%
B. canariense UBMAN05	93,3%	94,1%	94,1%	94,1%	94,1%	94,1%	94,1%	94,1%	94,6%	94,6%	93,3%	94,4%	93,8%	93,3%	93,3%	93,3%	94,6%	94,6%	94,1%	93,8%	94,6%	92,8%	94,4%	94,1%	94,1%	91,4%	94,1%	91,4%	92,0%	92,0%	93,0%	92,5%	92,2%	90,9%	92,5%	91,7%	97,6%	100,0%	100,0%	ID	100,0%	94,4%	94,4%	94,4%	93,6%	93,3%	94,4%	93,3%	90,9%	94,1%	96,0%	93,8%	94,1%	92,8%	94,4%	93,0%	92,5%	92,8%	93,8%	92,5%	92,2%	93,0%	92,2%	93,8%	89,8%	91,7%	90,1%	94,6%	85,6%
B. canariense UBMA183	93,3%	94,1%	94,1%	94,1%	94,1%	94,1%	94,1%	94,1%	94,6%	94,6%	93,3%	94,4%	93,8%	93,3%	93,3%	93,3%	94,6%	94,6%	94,1%	93,8%	94,6%	92,8%	94,4%	94,1%	94,1%	91,4%	94,1%	91,4%	92,0%	92,0%	93,0%	92,5%	92,2%	90,9%	92,5%	91,7%	97,6%	100,0%	100,0%	100,0%	ID	94,4%	94,4%	94,4%	93,6%	93,3%	94,4%	93,3%	90,9%	94,1%	96,0%	93,8%	94,1%	92,8%	94,4%	93,0%	92,5%	92,8%	93,8%	92,5%	92,2%	93,0%	92,2%	93,8%	89,8%	91,7%	90,1%	94,6%	85,6%
B. liaoningense LMG18230	93,0%	93,3%	93,3%	93,3%	93,3%	93,3%	93,3%	93,3%	93,3%	93,3%	92,5%	93,6%	93,6%	92,5%	92,5%	92,5%	93,3%	93,3%	93,3%	93,6%	93,3%	93,3%	94,1%	94,4%	94,4%	94,4%	94,4%	94,4%	93,8%	93,8%	96,0%	96,0%	95,7%	94,4%	96,0%	94,4%	94,1%	94,4%	94,4%	94,4%	94,4%	ID	100,0%	100,0%	98,6%	95,2%	100,0%	95,2%	94,1%	92,5%	94,1%	94,9%	92,2%	94,4%	96,2%	95,7%	93,3%	92,0%	93,0%	93,3%	91,4%	93,8%	92,8%	94,1%	90,9%	92,2%	91,2%	93,8%	86,6%
B. liaoningense CCBAU05525	93,0%	93,3%	93,3%	93,3%	93,3%	93,3%	93,3%	93,3%	93,3%	93,3%	92,5%	93,6%	93,6%	92,5%	92,5%	92,5%	93,3%	93,3%	93,3%	93,6%	93,3%	93,3%	94,1%	94,4%	94,4%	94,4%	94,4%	94,4%	93,8%	93,8%	96,0%	96,0%	95,7%	94,4%	96,0%	94,4%	94,1%	94,4%	94,4%	94,4%	94,4%	100,0%	ID	100,0%	98,6%	95,2%	100,0%	95,2%	94,1%	92,5%	94,1%	94,9%	92,2%	94,4%	96,2%	95,7%	93,3%	92,0%	93,0%	93,3%	91,4%	93,8%	92,8%	94,1%	90,9%	92,2%	91,2%	93,8%	86,6%
B. liaoningense CCBAU83689	93,0%	93,3%	93,3%	93,3%	93,3%	93,3%	93,3%	93,3%	93,3%	93,3%	92,5%	93,6%	93,6%	92,5%	92,5%	92,5%	93,3%	93,3%	93,3%	93,6%	93,3%	93,3%	94,1%	94,4%	94,4%	94,4%	94,4%	94,4%	93,8%	93,8%	96,0%	96,0%	95,7%	94,4%	96,0%	94,4%	94,1%	94,4%	94,4%	94,4%	94,4%	100,0%	100,0%	ID	98,6%	95,2%	100,0%	95,2%	94,1%	92,5%	94,1%	94,9%	92,2%	94,4%	96,2%	95,7%	93,3%	92,0%	93,0%	93,3%	91,4%	93,8%	92,8%	94,1%	90,9%	92,2%	91,2%	93,8%	86,6%
B. liaoningense CCNWSX0360	92,2%	92,5%	92,5%	92,5%	92,5%	92,5%	92,5%	92,5%	93,0%	93,0%	91,7%	93,3%	93,3%	91,7%	91,7%	91,7%	93,0%	93,0%	92,5%	93,3%	93,0%	92,5%	93,3%	93,6%	93,6%	93,0%	93,6%	93,0%	93,0%	93,0%	95,7%	95,2%	94,4%	93,0%	95,2%	94,1%	93,3%	93,6%	93,6%	93,6%	93,6%	98,6%	98,6%	98,6%	ID	95,2%	98,6%	95,2%	92,8%	91,7%	93,3%	94,6%	92,0%	94,1%	95,4%	95,4%	92,5%	91,7%	92,2%	93,0%	90,6%	93,6%	92,0%	93,6%	90,1%	91,4%	90,4%	93,6%	85,8%
B. japonicum USDA4	93,0%	92,5%	92,5%	92,8%	92,5%	92,5%	92,5%	92,5%	92,8%	92,8%	92,0%	93,0%	93,0%	91,7%	91,7%	91,7%	92,8%	92,8%	92,5%	93,0%	92,8%	91,4%	93,6%	93,8%	93,8%	91,7%	93,8%	91,7%	92,2%	92,2%	96,2%	93,3%	93,3%	91,7%	93,3%	92,2%	92,5%	93,3%	93,3%	93,3%	93,3%	95,2%	95,2%	95,2%	95,2%	ID	95,2%	100,0%	92,2%	90,9%	94,1%	94,1%	92,8%	93,8%	94,1%	93,6%	91,7%	91,4%	92,0%	93,6%	89,0%	92,8%	90,9%	93,0%	88,8%	89,8%	89,0%	92,2%	84,5%
B. japonicum USDA135	93,0%	93,3%	93,3%	93,3%	93,3%	93,3%	93,3%	93,3%	93,3%	93,3%	92,5%	93,6%	93,6%	92,5%	92,5%	92,5%	93,3%	93,3%	93,3%	93,6%	93,3%	93,3%	94,1%	94,4%	94,4%	94,4%	94,4%	94,4%	93,8%	93,8%	96,0%	96,0%	95,7%	94,4%	96,0%	94,4%	94,1%	94,4%	94,4%	94,4%	94,4%	100,0%	100,0%	100,0%	98,6%	95,2%	ID	95,2%	94,1%	92,5%	94,1%	94,9%	92,2%	94,4%	96,2%	95,7%	93,3%	92,0%	93,0%	93,3%	91,4%	93,8%	92,8%	94,1%	90,9%	92,2%	91,2%	93,8%	86,6%
B. ottawaense OO99	93,0%	92,5%	92,5%	92,8%	92,5%	92,5%	92,5%	92,5%	92,8%	92,8%	92,0%	93,0%	93,0%	91,7%	91,7%	91,7%	92,8%	92,8%	92,5%	93,0%	92,8%	91,4%	93,6%	93,8%	93,8%	91,7%	93,8%	91,7%	92,2%	92,2%	96,2%	93,3%	93,3%	91,7%	93,3%	92,2%	92,5%	93,3%	93,3%	93,3%	93,3%	95,2%	95,2%	95,2%	95,2%	100,0%	95,2%	ID	92,2%	90,9%	94,1%	94,1%	92,8%	93,8%	94,1%	93,6%	91,7%	91,4%	92,0%	93,6%	89,0%	92,8%	90,9%	93,0%	88,8%	89,8%	89,0%	92,2%	84,5%
B. daqingense CCBAU15774	91,7%	91,2%	91,2%	91,4%	91,2%	91,2%	91,2%	91,2%	90,6%	90,6%	90,1%	90,9%	91,2%	90,1%	90,1%	90,1%	90,9%	90,9%	91,2%	91,2%	90,6%	90,6%	91,4%	91,7%	91,7%	94,6%	91,7%	94,6%	92,5%	92,5%	93,3%	93,3%	93,3%	93,0%	93,3%	93,8%	90,6%	90,9%	90,9%	90,9%	90,9%	94,1%	94,1%	94,1%	92,8%	92,2%	94,1%	92,2%	ID	91,2%	90,4%	92,8%	89,6%	93,0%	93,3%	94,4%	90,4%	89,6%	90,9%	92,0%	88,8%	90,9%	90,6%	91,4%	87,4%	89,8%	88,5%	92,5%	85,8%
B. cytisi CTAW11	92,5%	93,0%	93,0%	93,3%	93,0%	93,0%	93,0%	93,0%	92,2%	92,2%	93,0%	93,0%	92,5%	92,2%	92,2%	92,2%	92,2%	92,2%	93,0%	92,5%	92,2%	92,0%	93,0%	92,8%	92,8%	92,0%	92,8%	92,0%	90,6%	90,6%	90,9%	90,4%	90,1%	91,2%	90,4%	91,4%	94,4%	94,1%	94,1%	94,1%	94,1%	92,5%	92,5%	92,5%	91,7%	90,9%	92,5%	90,9%	91,2%	ID	93,6%	92,2%	91,4%	92,2%	92,8%	92,2%	90,4%	91,4%	91,7%	91,4%	89,8%	90,9%	91,2%	90,9%	87,7%	90,1%	88,2%	92,5%	85,3%
B. rifense CTAW71	91,7%	92,2%	92,2%	92,5%	92,2%	92,2%	92,2%	92,2%	92,5%	92,5%	91,7%	92,8%	92,2%	91,7%	91,7%	91,7%	92,5%	92,5%	92,2%	92,2%	92,5%	91,4%	93,0%	92,8%	92,8%	92,5%	92,8%	92,5%	91,2%	91,2%	92,2%	91,7%	92,0%	91,2%	91,7%	91,4%	94,9%	96,0%	96,0%	96,0%	96,0%	94,1%	94,1%	94,1%	93,3%	94,1%	94,1%	94,1%	90,4%	93,6%	ID	93,3%	92,2%	93,3%	93,8%	92,8%	91,7%	91,4%	92,0%	91,7%	90,6%	91,7%	91,7%	92,0%	89,3%	90,6%	89,0%	92,2%	84,5%
B. arachidis CCBAU051107	93,3%	93,3%	93,3%	93,6%	93,3%	93,3%	93,3%	93,3%	93,6%	93,6%	92,8%	93,8%	93,3%	93,3%	93,3%	93,3%	93,6%	93,6%	93,3%	93,3%	93,6%	92,0%	93,3%	93,6%	93,6%	93,8%	93,6%	93,8%	92,0%	92,0%	94,1%	94,4%	94,1%	92,8%	94,4%	94,1%	93,0%	93,8%	93,8%	93,8%	93,8%	94,9%	94,9%	94,9%	94,6%	94,1%	94,9%	94,1%	92,8%	92,2%	93,3%	ID	92,2%	94,4%	96,5%	95,2%	93,0%	92,0%	92,8%	93,0%	91,2%	93,0%	91,7%	92,5%	90,1%	91,2%	90,6%	94,1%	87,2%
B. ganzhouense RITF806	92,5%	93,0%	93,0%	93,3%	93,0%	93,0%	93,0%	93,0%	92,8%	92,8%	92,5%	93,6%	93,0%	92,2%	92,2%	92,2%	92,8%	92,8%	93,0%	93,0%	92,8%	93,6%	94,4%	94,1%	94,1%	89,8%	94,1%	89,8%	92,0%	92,0%	93,3%	91,4%	91,4%	89,6%	91,4%	90,1%	92,8%	94,1%	94,1%	94,1%	94,1%	92,2%	92,2%	92,2%	92,0%	92,8%	92,2%	92,8%	89,6%	91,4%	92,2%	92,2%	ID	93,3%	93,3%	92,0%	90,4%	90,6%	90,4%	90,9%	89,6%	90,4%	89,3%	90,1%	87,4%	89,6%	87,7%	90,9%	84,2%
B. manausense BR3351	91,2%	91,2%	91,2%	91,4%	91,2%	91,2%	91,2%	91,2%	91,4%	91,4%	90,6%	91,7%	91,2%	90,6%	90,6%	90,6%	91,4%	91,4%	91,2%	91,2%	91,4%	91,7%	92,0%	92,2%	92,2%	94,1%	92,2%	94,1%	90,9%	90,9%	94,4%	92,8%	92,8%	93,3%	92,8%	94,6%	92,0%	92,8%	92,8%	92,8%	92,8%	94,4%	94,4%	94,4%	94,1%	93,8%	94,4%	93,8%	93,0%	92,2%	93,3%	94,4%	93,3%	ID	95,7%	94,1%	90,9%	91,7%	91,4%	93,3%	89,6%	92,0%	91,7%	91,7%	88,8%	90,1%	89,0%	92,5%	86,4%
B. stylosanthis BR446	93,6%	93,6%	93,6%	93,8%	93,6%	93,6%	93,6%	93,6%	93,8%	93,8%	93,6%	94,1%	93,6%	93,0%	93,0%	93,0%	93,8%	93,8%	93,6%	93,6%	93,8%	93,3%	94,1%	94,4%	94,4%	94,4%	94,4%	94,4%	92,8%	92,8%	95,4%	95,4%	95,2%	93,6%	95,4%	94,4%	93,6%	94,4%	94,4%	94,4%	94,4%	96,2%	96,2%	96,2%	95,4%	94,1%	96,2%	94,1%	93,3%	92,8%	93,8%	96,5%	93,3%	95,7%	ID	95,4%	93,8%	93,0%	93,8%	93,8%	92,0%	94,9%	93,3%	93,6%	91,2%	92,2%	91,4%	94,9%	87,7%
B. neotropicale BR10247	92,8%	92,8%	92,8%	93,0%	92,8%	92,8%	92,8%	92,8%	92,5%	92,5%	91,7%	92,8%	92,8%	92,0%	92,0%	92,0%	92,5%	92,5%	92,8%	92,8%	92,5%	92,0%	92,8%	93,0%	93,0%	93,6%	93,0%	93,6%	92,8%	92,8%	94,6%	94,4%	94,1%	92,8%	94,4%	93,3%	92,2%	93,0%	93,0%	93,0%	93,0%	95,7%	95,7%	95,7%	95,4%	93,6%	95,7%	93,6%	94,4%	92,2%	92,8%	95,2%	92,0%	94,1%	95,4%	ID	90,6%	91,4%	91,2%	92,8%	89,8%	91,4%	90,9%	91,7%	89,0%	90,4%	89,6%	93,0%	86,4%
B. embrapense SEMIA6208	92,2%	92,0%	92,0%	92,0%	92,0%	92,0%	92,0%	92,0%	91,4%	91,4%	91,7%	92,2%	91,7%	91,4%	91,4%	91,4%	91,4%	91,4%	92,0%	91,7%	91,4%	91,7%	92,8%	93,0%	93,0%	90,4%	93,0%	90,4%	92,0%	92,0%	92,5%	91,4%	91,7%	89,8%	91,4%	91,2%	92,8%	92,5%	92,5%	92,5%	92,5%	93,3%	93,3%	93,3%	92,5%	91,7%	93,3%	91,7%	90,4%	90,4%	91,7%	93,0%	90,4%	90,9%	93,8%	90,6%	ID	93,0%	94,6%	92,5%	93,6%	95,2%	93,6%	94,4%	90,9%	93,3%	91,2%	94,6%	88,0%
B. valentinum LmjM3	90,9%	91,2%	91,2%	91,2%	91,2%	91,2%	91,2%	91,2%	90,9%	90,9%	91,4%	91,4%	90,9%	90,4%	90,4%	90,4%	90,9%	90,9%	91,2%	90,9%	90,9%	90,9%	92,2%	92,5%	92,5%	90,4%	92,5%	90,4%	89,3%	89,3%	92,0%	90,9%	90,9%	89,6%	90,9%	90,4%	92,8%	92,8%	92,8%	92,8%	92,8%	92,0%	92,0%	92,0%	91,7%	91,4%	92,0%	91,4%	89,6%	91,4%	91,4%	92,0%	90,6%	91,7%	93,0%	91,4%	93,0%	ID	94,1%	93,6%	93,3%	92,8%	95,2%	92,0%	92,8%	94,6%	92,2%	94,6%	87,2%
B. elkanii USDA76	92,5%	92,2%	92,2%	92,2%	92,2%	92,2%	92,2%	92,2%	92,2%	92,2%	92,0%	92,5%	92,0%	92,0%	92,0%	92,0%	92,2%	92,2%	92,2%	92,0%	92,2%	92,0%	93,0%	93,3%	93,3%	90,1%	93,3%	90,1%	92,2%	92,2%	92,8%	92,2%	92,5%	91,2%	92,2%	91,4%	93,6%	93,8%	93,8%	93,8%	93,8%	93,0%	93,0%	93,0%	92,2%	92,0%	93,0%	92,0%	90,9%	91,7%	92,0%	92,8%	90,4%	91,4%	93,8%	91,2%	94,6%	94,1%	ID	93,0%	92,8%	94,6%	93,6%	95,4%	90,9%	93,0%	90,6%	95,2%	87,2%
B. erythrophlei CCBAU53325	90,4%	90,6%	90,6%	90,6%	90,6%	90,6%	90,6%	90,6%	90,1%	90,1%	89,3%	90,4%	90,4%	89,3%	89,3%	89,3%	90,1%	90,1%	90,6%	90,4%	90,1%	89,8%	91,4%	91,2%	91,2%	91,7%	91,2%	91,7%	90,6%	90,6%	93,3%	92,8%	92,8%	92,0%	92,8%	92,8%	92,2%	92,5%	92,5%	92,5%	92,5%	93,3%	93,3%	93,3%	93,0%	93,6%	93,3%	93,6%	92,0%	91,4%	91,7%	93,0%	90,9%	93,3%	93,8%	92,8%	92,5%	93,6%	93,0%	ID	91,2%	94,1%	92,8%	93,3%	90,4%	90,9%	90,9%	95,7%	87,2%
B. retamae Ro19	91,7%	91,7%	91,7%	91,7%	91,7%	91,7%	91,7%	91,7%	90,9%	90,9%	91,4%	91,4%	91,4%	91,4%	91,4%	91,4%	91,4%	91,4%	91,7%	91,4%	91,4%	90,9%	90,6%	91,7%	92,0%	92,0%	90,4%	92,0%	90,4%	89,0%	89,0%	89,6%	91,2%	90,9%	89,6%	91,2%	90,4%	92,5%	92,2%	92,2%	92,2%	92,2%	91,4%	91,4%	91,4%	90,6%	89,0%	91,4%	89,0%	88,8%	89,8%	90,6%	91,2%	89,6%	89,6%	92,0%	89,8%	93,6%	93,3%	92,8%	91,2%	ID	93,0%	95,4%	92,0%	92,8%	94,9%	93,8%	93,6%	87,2%
B. viridifuturi SEMIA690	91,2%	91,4%	91,4%	91,4%	91,4%	91,4%	91,4%	91,4%	92,0%	92,0%	91,2%	92,2%	91,7%	90,4%	90,4%	90,4%	92,0%	92,0%	91,4%	91,7%	92,0%	91,2%	92,8%	93,0%	93,0%	91,2%	93,0%	91,2%	90,9%	90,9%	93,0%	93,0%	92,2%	91,4%	93,0%	92,2%	93,3%	93,0%	93,0%	93,0%	93,0%	93,8%	93,8%	93,8%	93,6%	92,8%	93,8%	92,8%	90,9%	90,9%	91,7%	93,0%	90,4%	92,0%	94,9%	91,4%	95,2%	92,8%	94,6%	94,1%	93,0%	ID	94,4%	95,2%	92,2%	92,2%	92,0%	96,8%	88,2%
B. lablabi CCBAU23086	90,9%	91,2%	91,2%	91,2%	91,2%	91,2%	91,2%	91,2%	91,2%	91,2%	91,4%	91,2%	90,9%	90,4%	90,4%	90,4%	91,4%	91,4%	91,2%	90,9%	91,2%	90,4%	92,0%	92,2%	92,2%	91,4%	92,2%	91,4%	90,4%	90,4%	91,2%	92,0%	92,0%	90,6%	92,0%	91,4%	93,3%	92,2%	92,2%	92,2%	92,2%	92,8%	92,8%	92,8%	92,0%	90,9%	92,8%	90,9%	90,6%	91,2%	91,7%	91,7%	89,3%	91,7%	93,3%	90,9%	93,6%	95,2%	93,6%	92,8%	95,4%	94,4%	ID	92,5%	94,4%	95,2%	94,4%	93,6%	88,2%
B. pachyrhizi LMG24246	91,4%	91,2%	91,2%	91,2%	91,2%	91,2%	91,2%	91,2%	91,4%	91,4%	90,4%	91,7%	91,2%	90,4%	90,4%	90,4%	91,4%	91,4%	91,2%	91,2%	91,4%	90,4%	92,0%	92,2%	92,2%	90,4%	92,2%	90,4%	91,2%	91,2%	93,3%	93,0%	93,0%	91,7%	93,0%	92,0%	93,6%	93,8%	93,8%	93,8%	93,8%	94,1%	94,1%	94,1%	93,6%	93,0%	94,1%	93,0%	91,4%	90,9%	92,0%	92,5%	90,1%	91,7%	93,6%	91,7%	94,4%	92,0%	95,4%	93,3%	92,0%	95,2%	92,5%	ID	89,3%	91,4%	89,6%	95,7%	87,7%
B. jicamae LMG24556	88,5%	88,8%	88,8%	88,8%	88,8%	88,8%	88,8%	88,8%	89,0%	89,0%	89,0%	89,0%	89,0%	88,0%	88,0%	88,0%	89,0%	89,0%	88,8%	89,0%	89,0%	88,8%	89,6%	89,8%	89,8%	89,3%	89,8%	89,3%	88,0%	88,0%	89,0%	89,6%	90,4%	88,8%	89,6%	89,0%	90,4%	89,8%	89,8%	89,8%	89,8%	90,9%	90,9%	90,9%	90,1%	88,8%	90,9%	88,8%	87,4%	87,7%	89,3%	90,1%	87,4%	88,8%	91,2%	89,0%	90,9%	92,8%	90,9%	90,4%	92,8%	92,2%	94,4%	89,3%	ID	92,5%	98,1%	90,9%	88,0%
B. icense LMTR13	90,6%	90,9%	90,9%	90,9%	90,9%	90,9%	90,9%	90,9%	90,4%	90,4%	91,7%	90,9%	90,6%	90,6%	90,6%	90,6%	90,6%	90,6%	90,9%	90,6%	90,4%	90,9%	92,0%	92,2%	92,2%	89,8%	92,2%	89,8%	89,3%	89,3%	90,1%	90,4%	90,6%	89,3%	90,4%	90,1%	92,8%	91,7%	91,7%	91,7%	91,7%	92,2%	92,2%	92,2%	91,4%	89,8%	92,2%	89,8%	89,8%	90,1%	90,6%	91,2%	89,6%	90,1%	92,2%	90,4%	93,3%	94,6%	93,0%	90,9%	94,9%	92,2%	95,2%	91,4%	92,5%	ID	93,3%	92,8%	87,7%
B. paxllaeri LMTR21	89,0%	89,3%	89,3%	89,3%	89,3%	89,3%	89,3%	89,3%	89,3%	89,3%	89,6%	89,3%	89,6%	88,5%	88,5%	88,5%	89,6%	89,6%	89,3%	89,6%	89,3%	88,8%	89,8%	90,1%	90,1%	89,3%	90,1%	89,3%	88,2%	88,2%	89,3%	90,4%	90,6%	89,0%	90,4%	89,8%	91,2%	90,1%	90,1%	90,1%	90,1%	91,2%	91,2%	91,2%	90,4%	89,0%	91,2%	89,0%	88,5%	88,2%	89,0%	90,6%	87,7%	89,0%	91,4%	89,6%	91,2%	92,2%	90,6%	90,9%	93,8%	92,0%	94,4%	89,6%	98,1%	93,3%	ID	91,4%	88,8%
B. mercantei SEMIA6399	92,2%	92,5%	92,5%	92,5%	92,5%	92,5%	92,5%	92,5%	92,5%	92,5%	91,2%	92,8%	92,2%	91,2%	91,2%	91,2%	92,5%	92,5%	92,5%	92,2%	92,5%	91,2%	92,8%	93,0%	93,0%	91,7%	93,0%	91,7%	91,4%	91,4%	93,0%	93,8%	93,0%	91,7%	93,8%	93,0%	93,8%	94,6%	94,6%	94,6%	94,6%	93,8%	93,8%	93,8%	93,6%	92,2%	93,8%	92,2%	92,5%	92,5%	92,2%	94,1%	90,9%	92,5%	94,9%	93,0%	94,6%	94,6%	95,2%	95,7%	93,6%	96,8%	93,6%	95,7%	90,9%	92,8%	91,4%	ID	88,5%
Rhodopseudomonas palustris CGA009	84,8%	84,5%	84,5%	84,5%	84,5%	84,5%	84,5%	84,5%	84,0%	84,0%	85,6%	84,2%	83,7%	84,0%	84,0%	84,0%	84,0%	84,0%	84,5%	83,7%	84,0%	85,3%	86,1%	85,8%	85,8%	86,1%	85,8%	86,1%	85,8%	85,8%	86,4%	86,4%	86,6%	85,6%	86,4%	86,9%	86,6%	85,6%	85,6%	85,6%	85,6%	86,6%	86,6%	86,6%	85,8%	84,5%	86,6%	84,5%	85,8%	85,3%	84,5%	87,2%	84,2%	86,4%	87,7%	86,4%	88,0%	87,2%	87,2%	87,2%	87,2%	88,2%	88,2%	87,7%	88,0%	87,7%	88,8%	88,5%	ID

Supplementary Table S5. *ftsA* gene sequence similarity between 176 bradyrhizobial and *Bosea* strains used in the study

	B. japonicum USDA6	*Bradyrhizobium* sp. JAN6	*Bradyrhizobium* sp. JAN10	*Bradyrhizobium* sp. GER1	*Bradyrhizobium* sp. GER32	*Bradyrhizobium* sp. ZAR4	*Bradyrhizobium* sp. ZAR12	*Bradyrhizobium* sp. SZCZ11	*Bradyrhizobium* sp. SZCZ20	*B. japonicum* E109	*B. japonicum* J5	*B. japonicum* SEMIA5079	*B. japonicum* CCBAU83623	*B. japonicum* FN1	*B. japonicum* UBMA197	*B. japonicum* USDA38	*B. japonicum* USDA123	*B. japonicum* CCBAU15354	*B. japonicum* CCBAU15517	*B. japonicum* CCBAU15618
B. japonicum USDA6	ID	97,6%	97,5%	97,6%	97,6%	97,6%	97,6%	98,2%	98,2%	100,0%	98,0%	99,5%	98,0%	100,0%	97,6%	98,7%	98,0%	98,0%	98,0%	98,9%
Bradyrhizobium sp. JAN6	97,6%	ID	97,9%	100,0%	100,0%	100,0%	100,0%	98,9%	98,9%	97,6%	98,5%	97,8%	98,3%	97,6%	100,0%	98,3%	98,3%	98,3%	98,3%	97,4%
Bradyrhizobium sp. JAN10	97,5%	97,9%	ID	97,9%	97,9%	97,9%	97,9%	98,2%	98,2%	97,5%	98,0%	97,4%	97,6%	97,5%	97,9%	97,9%	97,6%	97,6%	97,6%	97,0%
Bradyrhizobium sp. GER1	97,6%	100,0%	97,9%	ID	100,0%	100,0%	100,0%	98,9%	98,9%	97,6%	98,5%	97,8%	98,3%	97,6%	100,0%	98,3%	98,3%	98,3%	98,3%	97,4%
Bradyrhizobium sp. GER32	97,6%	100,0%	97,9%	100,0%	ID	100,0%	100,0%	98,9%	98,9%	97,6%	98,5%	97,8%	98,3%	97,6%	100,0%	98,3%	98,3%	98,3%	98,3%	97,4%
Bradyrhizobium sp. ZAR4	97,6%	100,0%	97,9%	100,0%	100,0%	ID	100,0%	98,9%	98,9%	97,6%	98,5%	97,8%	98,3%	97,6%	100,0%	98,3%	98,3%	98,3%	98,3%	97,4%
Bradyrhizobium sp. ZAR12	97,6%	100,0%	97,9%	100,0%	100,0%	100,0%	ID	98,9%	98,9%	97,6%	98,5%	97,8%	98,3%	97,6%	100,0%	98,3%	98,3%	98,3%	98,3%	97,4%
Bradyrhizobium sp. SZCZ11	98,2%	98,9%	98,2%	98,9%	98,9%	98,9%	98,9%	ID	100,0%	98,2%	98,7%	97,8%	98,7%	98,2%	98,9%	98,6%	98,7%	98,7%	98,7%	97,4%
Bradyrhizobium sp. SZCZ20	98,2%	98,9%	98,2%	98,9%	98,9%	98,9%	98,9%	100,0%	ID	98,2%	98,7%	97,8%	98,7%	98,2%	98,9%	98,6%	98,7%	98,7%	98,7%	97,4%
B. japonicum E109	100,0%	97,6%	97,5%	97,6%	97,6%	97,6%	97,6%	98,2%	98,2%	ID	98,0%	99,5%	98,0%	100,0%	97,6%	98,7%	98,0%	98,0%	98,0%	98,9%
B. japonicum J5	98,0%	98,5%	98,0%	98,5%	98,5%	98,5%	98,5%	98,7%	98,7%	98,0%	ID	97,9%	98,5%	98,0%	98,5%	98,5%	98,5%	98,5%	98,5%	97,5%
B. japonicum SEMIA5079	99,5%	97,8%	97,4%	97,8%	97,8%	97,8%	97,8%	97,8%	97,8%	99,5%	97,9%	ID	97,8%	99,5%	97,8%	98,6%	97,8%	97,8%	97,8%	99,3%
B. japonicum CCBAU83623	98,0%	98,3%	97,6%	98,3%	98,3%	98,3%	98,3%	98,7%	98,7%	98,0%	98,5%	97,8%	ID	98,0%	98,3%	98,6%	100,0%	100,0%	100,0%	97,4%
B. japonicum FN1	100,0%	97,6%	97,5%	97,6%	97,6%	97,6%	97,6%	98,2%	98,2%	100,0%	98,0%	99,5%	98,0%	ID	97,6%	98,7%	98,0%	98,0%	98,0%	98,9%
B. japonicum UBMA197	97,6%	100,0%	97,9%	100,0%	100,0%	100,0%	100,0%	98,9%	98,9%	97,6%	98,5%	97,8%	98,3%	97,6%	ID	98,3%	98,3%	98,3%	98,3%	97,4%
B. japonicum USDA38	98,7%	98,3%	97,9%	98,3%	98,3%	98,3%	98,3%	98,6%	98,6%	98,7%	98,5%	98,6%	98,6%	98,7%	98,3%	ID	98,6%	98,6%	98,6%	98,2%
B. japonicum USDA123	98,0%	98,3%	97,6%	98,3%	98,3%	98,3%	98,3%	98,7%	98,7%	98,0%	98,5%	97,8%	100,0%	98,0%	98,3%	98,6%	ID	100,0%	100,0%	97,4%
B. japonicum CCBAU15354	98,0%	98,3%	97,6%	98,3%	98,3%	98,3%	98,3%	98,7%	98,7%	98,0%	98,5%	97,8%	100,0%	98,0%	98,3%	98,6%	100,0%	ID	100,0%	97,4%
B. japonicum CCBAU15517	98,0%	98,3%	97,6%	98,3%	98,3%	98,3%	98,3%	98,7%	98,7%	98,0%	98,5%	97,8%	100,0%	98,0%	98,3%	98,6%	100,0%	100,0%	ID	97,4%
B. japonicum CCBAU15618	98,9%	97,4%	97,0%	97,4%	97,4%	97,4%	97,4%	97,4%	97,4%	98,9%	97,5%	99,3%	97,4%	98,9%	97,4%	98,2%	97,4%	97,4%	97,4%	ID
B. japonicum CCBAU25435	99,1%	97,4%	97,2%	97,4%	97,4%	97,4%	97,4%	97,6%	97,6%	99,1%	97,8%	99,0%	97,6%	99,1%	97,4%	98,5%	97,6%	97,6%	97,6%	99,4%
B. japonicum Is-34	98,7%	98,3%	97,9%	98,3%	98,3%	98,3%	98,3%	98,6%	98,6%	98,7%	98,5%	98,6%	98,6%	98,7%	98,3%	99,4%	98,6%	98,6%	98,6%	98,2%
B. japonicum Blup-MR1	97,5%	97,9%	100,0%	97,9%	97,9%	97,9%	97,9%	98,2%	98,2%	97,5%	98,0%	97,4%	97,6%	97,5%	97,9%	97,9%	97,6%	97,6%	97,6%	97,0%

[illegible]
